# Seasonal dynamics and molecular regulation of flavonoid biosynthesis in *Cyclocarya paliurus* (Batal.) Iljinsk

**DOI:** 10.3389/fpls.2025.1525226

**Published:** 2025-03-04

**Authors:** Duo Chen, Yixin Xiao, Xuehai Zheng, Huamiao Sun, Cifeng Zhang, Jinmao Zhu, Ting Xue

**Affiliations:** The Public Service Platform for Industrialization Development Technology of Marine Biological Medicine and Products of the Department of Natural Resources, Fujian Key Laboratory of Special Marine Bioresource Sustainable Utilization, Southern Institute of Oceanography, College of Life Sciences, Fujian Normal University, Fuzhou, China

**Keywords:** *Cyclocarya paliurus*, flavonoid biosynthesis, seasonal variation, transcriptomics, metabolomics

## Abstract

**Introduction:**

*Cyclocarya paliurus*, an economically important species known for its high flavonoid content, has potential for industrial applications. Understanding the seasonal dynamics and molecular regulation of flavonoid biosynthesis in this species is crucial for optimizing its production.

**Methods:**

We conducted an integrated analysis of transcriptomic and metabolomic data to identify key genes involved in flavonoid biosynthesis and regulation. Seasonal variation in flavonoid content and gene expression was examined, with a focus on the genes involved in the flavonoid synthesis pathway and their correlation with flavonoid levels.

**Results:**

Flavonoid content peaked in August and declined towards November, with quercetin and kaempferol glycosides being the most abundant compounds. Pearson correlation analysis revealed significant relationships between the functional genes of the flavonoid synthesis pathway and flavonoid content. Seasonal variations in the expression of key biosynthetic genes (*CHS, CHI, F3H, DFR, FLS*) and regulatory transcription factors (*MYB11, MYB12, MYB111, MYB75, MYB90, bHLH, WD40*) were strongly correlated with flavonoid levels, particularly under environmental stress.

**Discussion:**

These findings provide insights into the genetic regulation of flavonoid biosynthesis in *C. paliurus* and highlight the importance of seasonal and environmental factors. This knowledge has practical implications for industrial breeding and biotechnological applications, particularly in enhancing the functional properties of *C. paliurus* for industrial use. Our study establishes a foundation for future research aimed at optimizing flavonoid production in this species and exploring its potential for bioactive compound production.

## 1 Introduction

*Cyclocarya paliurus* (Batal.) Iljinsk., also known as the wheel wingnut or golden leaf tree, is a deciduous species in the Juglandaceae family, native to central and southern China. This tree can grow up to 30 meters tall and features a broad canopy with pinnate leaves that turn golden-yellow in autumn, enhancing its ornamental value (Shang et al., 2015; Zou et al., 2018; Zhao, 2020; Zheng et al., 2020). *C. paliurus* produces small monoecious flowers and winged nuts for wind dispersal, thriving in warm, humid climates and mixed forests at elevations of 500-2000 meters (Sun et al., 2021). Its ecological roles include providing wildlife habitat and aiding in soil stabilization.

The leaves of *C. paliurus* are rich in flavonoids, bioactive compounds known for their antioxidant, anti-inflammatory, and hypoglycemic properties. These flavonoids contribute to the plant ecological adaptability and have significant medicinal potential (Xie et al., 2015). In traditional Chinese medicine, the leaves are used for their health benefits, including anti-inflammatory and hypoglycemic effects (Zhou et al., 2019). Despite not being endangered, *C. paliurus* faces threats from habitat loss and overharvesting, making conservation efforts necessary (Chen et al., 2022).

*C. paliurus* shows seasonal variations in leaf production, which is most abundant during summer and autumn, particularly from August to November. This period is critical for studying the leaf biochemical and physiological properties, as production decreases in spring and winter. Our research focuses on differences in the metabolome and transcriptome of leaves collected from August to November, providing insights into the biosynthetic pathways regulating flavonoid production during peak growth (Fu et al., 2015).

Flavonoids, present in many fruits and vegetables, are essential for plant physiology, aiding in UV filtration, nitrogen fixation, and floral pigmentation to attract pollinators (Kumar and Pandey, 2013). They also protect plants from oxidative stress and act as defense agents against herbivores and pathogens (Liu et al., 2021). Additionally, flavonoids are involved in signal transduction, regulating various developmental pathways and stress responses (Falcone Ferreyra et al., 2012). In humans, flavonoids help neutralize free radicals, reducing the risk of chronic diseases like cancer and cardiovascular conditions, while supporting metabolic health (Liu et al., 2018; Hussain et al., 2020).

While previous studies, such as Sheng et al., provided insights into the flavonoid biosynthesis in *C. paliurus*, they did not examine how this process varies across different seasons (Sheng et al., 2021). Our study aims to fill this gap by investigating the temporal regulation of flavonoid biosynthesis from August to November, offering new insights into seasonal variations and regulatory mechanisms. By integrating transcriptomic, metabolomic, and seasonal data, we seek to optimize the harvesting periods for flavonoids, a novel perspective not explored in earlier studies.

Additionally, we previously reported the genome assembly of *C. paliurus* (~634.90 Mb), providing a foundation for further research on its nutrient metabolites (Zheng et al., 2021). This study aims to investigate the seasonal variation in flavonoid content from August to November, compare the transcriptomic and metabolomic profiles involved in flavonoid synthesis, and identify key changes in flavonoid compounds and the genes responsible for their synthesis. These insights are crucial for agriculture and medicine by enhancing our understanding of flavonoid biosynthesis regulation and contributing to the sustainable use of *C. paliurus*.

## 2 Materials and methods

### 2.1 Plant material collection

The study site for collecting *C. paliurus* leaves is located in Taozhou Township, Anxi County, Fujian Province, China (E117°45′42′, N25°22′48′). Samples were collected from August to November 2021, with 4 to 6 plants of similar height and morphology selected from the site. Midsections of leaves (5-10 cm in length) from 10 to 30 leaves per plant were harvested and pooled to form experimental samples. After collection, the samples were immediately placed in zip-lock bags, frozen in liquid nitrogen, and stored at -80°C (Zheng et al., 2021). The leaf samples were divided into three biological replicates for the determination of total flavonoid content. Leaf samples from August, September, October, and November were divided into three biological replicates (8M-ML1/2/3, 9M-ML1/2/3, 10M-ML1/2/3, 11M-ML1/2/3) for metabolomics and transcriptomics analyses. Prior to detection, the samples were freeze-dried.

### 2.2 Flavonoid content and metabolite analysis for *C. paliurus*

#### 2.2.1 Sample preparation and extraction

Biological samples were freeze-dried using a vacuum freeze-dryer (Scientz-100F). The freeze-dried samples were then crushed using a mixer mill (MM 400, Retsch) with a zirconia bead for 1.5 minutes at 30 Hz. A 100 mg portion of the lyophilized powder was dissolved in 1.2 ml of 70% methanol solution. The mixture was vortexed for 30 seconds every 30 minutes, repeated six times, and stored at 4°C overnight. After centrifugation at 12,000 rpm for 10 minutes, the extracts were filtered through a 0.22 μm pore size filter (SCAA-104; ANPEL, Shanghai, China) before undergoing UPLC-MS/MS analysis (Luo et al., 2013).

#### 2.2.2 Determination of flavonoid content

For the rutin standard solution, 0.05 g of rutin was dissolved in anhydrous ethanol in a 50 mL volumetric flask. Aluminum nitrate solution was prepared by dissolving 17.60 g of aluminum nitrate nonahydrate in water, and potassium acetate solution was prepared by dissolving 9.814 g of potassium acetate in water, with both solutions adjusted to 100 mL. Calibration was performed by placing 1.0 to 5.0 mL of the rutin solution in separate 50 mL volumetric flasks and filling them to 15 mL with ethanol. Subsequently, 1.00 mL each of the aluminum nitrate and potassium acetate solutions was added, and the final volume was adjusted with water. After standing for 1 hour, absorbance was measured at 420 nm. *C. paliurus* leaves were freeze-dried for 24 hours, and then 1.50 g of the dried sample was ground. The sample was extracted with 30 mL of ethanol at 40°C using ultrasound for 1 hour, with shaking every 20 minutes. The solution was then filtered and brought to a final volume of 50 mL with ethanol. For analysis, 1.0 mL of this extract was diluted to 15.0 mL with ethanol, and the flavonoid content was measured using the standard curve (Liu et al., 2016).

#### 2.2.3 Metabolite analysis for *C. paliurus*

The sample extracts were analyzed using a UPLC-ESI-MS/MS system (UPLC, SHIMADZU Nexera X2; MS, Applied Biosystems 4500 Q TRAP). The UPLC utilized an Agilent SB-C18 column (1.8 µm, 2.1 mm x 100 mm). The mobile phase consisted of solvent A (pure water with 0.1% formic acid) and solvent B (acetonitrile with 0.1% formic acid). The gradient program started with 95% A and 5% B, transitioning linearly to 5% A and 95% B within 9 minutes, maintaining this ratio for 1 minute. The program then reverted to 95% A and 5% B over 1.1 minutes, held for 2.9 minutes. The flow rate was 0.35 ml/min, the column oven was set to 40°C, and the injection volume was 4 μl. The effluent was connected to an ESI-triple quadrupole-linear ion trap (QTRAP)-MS. LIT and triple quadrupole (QQQ) scans were acquired using an AB4500 Q TRAP UPLC/MS/MS System with an ESI Turbo Ion-Spray interface, operating in both positive and negative ion modes, controlled by Analyst 1.6.3 software (AB Sciex). The ESI source parameters were: source temperature 550°C, ion spray voltage 5500 V (positive)/-4500 V (negative), ion source gas I and II at 50 and 60 psi, respectively, curtain gas at 25 psi, and high collision-activated dissociation (CAD). Instrument tuning and mass calibration were performed with 10 and 100 μmol/L polypropylene glycol solutions in QQQ and LIT modes. QQQ scans were acquired as MRM experiments with nitrogen collision gas set to medium. Specific MRM transitions were monitored for each period based on the eluted metabolites (Zheng et al., 2022).

### 2.3 Transcriptomics Analysis *C. paliurus*

Total RNA was extracted using the RNeasy Plant Mini Kit (Tiangen Bio, Beijing, China). For library construction, both rRNA-depleted stranded RNA-seq and small RNA sequencing were performed using the TruSeq Stranded Total RNA Prep Kit (Illumina, San Diego, USA). Sequence alignment to the reference genome was conducted using HISAT2, and FPKM values for mRNAs were calculated with StringTie (version 1.3.1). Differential expression analysis between the two conditions/groups was carried out using the DESeq R package (version 1.10.1). DESeq provides statistical routines for determining differential expression in digital gene expression data, utilizing a model based on the negative binomial distribution. The resulting p-values were adjusted for multiple testing using the Benjamini and Hochberg approach to control the false discovery rate. Genes with an adjusted p-value of <0.01 and an absolute log2(fold change) >1, as identified by DESeq, were designated as differentially expressed. Gene function annotation was performed using several databases, including Nr (NCBI non-redundant protein sequences), Pfam (Protein family), KOG/COG (Clusters of Orthologous Groups of proteins), Swiss-Prot (a manually annotated and reviewed protein sequence database), and KEGG (Kyoto Encyclopedia of Genes and Genomes). Gene Ontology (GO) enrichment analysis of the differentially expressed genes (*DEGs*) was conducted using the topGO R package. Additionally, statistical enrichment of differentially expressed genes in KEGG pathways was tested using KOBAS software (Mao et al., 2005; Trapnell et al., 2009).

### 2.4 Pearson correlation analysis of gene-flavonoid relationships

We selected 39 flavonoid substances with high content from 144 flavonoid substances, which accounted for more than 80% of the total flavonoid substances. Pearson correlation analysis was carried out between the seasonal variation of the contents of 39 flavonoids and the seasonal variation of the expression dose of 66 functional genes related with the flavonoid synthesis pathway and 11 transcription factors. Pearson correlation coefficient and pvalue matrix are generated. When the Pearson correlation coefficient of a pair of flavonoid content and gene expression dose data was greater than 0.8, and pvalue was less than 0.05, a significant correlation was determined.

## 3 Results

### 3.1 Determination of total flavonoid content in *C. paliurus* during different months

**Figure 1:**
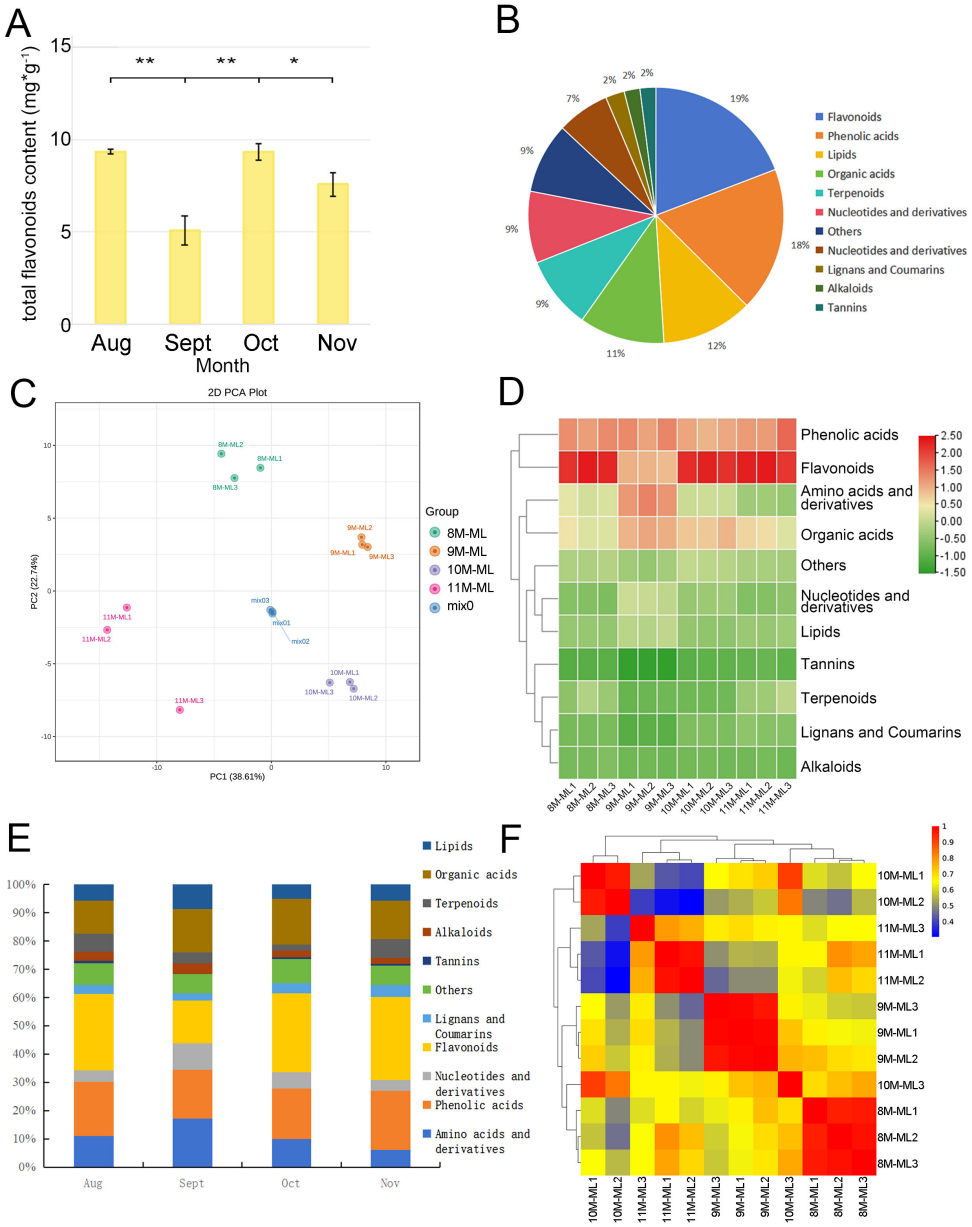
Characteristics of flavonoids in *C*. *paliurus*. **(A)** Total flavonoid content in *C*. *paliurus* leaves across different months. **(B)** Distribution of various metabolites in *C. paliurus* leaves. **(C)** PCA score plot showing the clustering of different leaf samples. **(D)** Heatmap clustering of the identified compounds. **(E)** Trends in the relative abundance of different compound classes from August to November. **(F)** Correlation matrix among leaf samples based on their metabolite profiles.

Based on **Figure 1A**, the total flavonoid content in *C. paliurus* leaves shows significant seasonal variation from August to November. In August, the flavonoid content is at its peak, reaching approximately 10 mg/g. This high level decreases sharply in September, dropping to around half of the August value. However, in October, the flavonoid content increases again to a level comparable to that in August, indicating a partial recovery. By November, the flavonoid level declines slightly from October, reaching about 80% of the October content. Statistical analysis reveals significant differences between these months, as indicated by the asterisks (**p < 0.01, *p < 0.05), suggesting that environmental factors and seasonal cues may influence flavonoid biosynthesis in *C. paliurus* leaves across these months. This pattern highlights a dynamic adjustment in flavonoid production, possibly linked to the plant’s adaptive response to seasonal changes.

### 3.2 Flavonoid metabolic differences of *C. paliurus* leaves during different months

The analysis revealed the presence of 751 secondary metabolites in *C. paliurus*, including amino acids and their derivatives, phenolic acids, nucleotides and their derivatives, flavonoids, lignans, coumarins, tannins, alkaloids, terpenes, organic acids, lipids, and other metabolites. **Figure 1B** illustrates the distribution of various metabolite categories in *C. paliurus* leaves, with flavonoids and nucleotides dominating the distribution, totaling 144 and 137 metabolites, respectively, accounting for more than 19.17% of the total metabolites. Other significant categories include terpenoids (87), amino acids (81), and lipids (69).

The PCA score plots for each sampling time (**Figure 1C**) show clear separation between samples collected during different months, indicating significant variation in substance accumulation at the metabolomic level. The data of flavonoid metabolites in *C. paliurus* leaves from different harvesting periods were analyzed by PCA, and the results are presented in **Figure 1C**, where each point represents one sample. The results indicate that the samples from the four harvesting periods were distinctly separated, highlighting the differences in flavonoid components across these periods. The groups from August (8M-ML) and September (9M-ML) were closely positioned on the score map, suggesting that the flavonoid components in the leaves from these two months were similar. Although PCA and the Pearson correlation coefficient algorithm are different, both methods reveal significant differences in terpenoid composition from August to November.

In our study, the data were normalized, and the accumulation patterns of metabolites in different samples were analyzed through clustering. **Figure 1D** presents the clustering results, which show good intra-group repeatability for the leaf samples from the four harvesting periods. A significant difference in the distribution of leaf components in September was observed compared to the other months, while the distribution in the other three months was relatively similar. The metabolites during the four harvesting stages were primarily concentrated in phenolic acids, flavonoids, amino acids, and organic acids.

All flavonoids in the metabolome of *C. paliurus* from August to November were selected for further analysis. The bar accumulation diagram in **Figure 1E** illustrates the seasonal variation in the proportion of flavonoids in *C. paliurus* leaves across the four harvesting periods: August, September, October, and November. In August, flavonoids represented approximately 26%∼28% of the total metabolites, reflecting peak biosynthetic activity during the summer, when environmental conditions were optimal for flavonoid production. As the season transitioned into September, the proportion of flavonoids slightly decreased to around 15%, likely due to cooler weather and shorter daylight hours, which may have shifted the plant’s metabolic focus. In October, the flavonoid content increased again, reaching 27%∼29%, and in November, the flavonoid content reached the highest level, at 27%∼31% (**Supplementary Table S1**). This trend highlights *C. paliurus*’ adaptive strategy to modulate flavonoid biosynthesis in response to seasonal changes, optimizing its metabolic processes to ensure survival and sustainability under varying environmental conditions.

By calculating the Pearson correlation coefficient, as shown in **Figure 1F**, the heatmap visually represents the correlation between leaf samples of *C. paliurus* collected from August to November, with the color gradient indicating the strength of these correlations (red for high, blue for low). Samples collected within the same month, such as those from August, September, October, and November, exhibited strong correlations, indicated by red areas, suggesting consistency in their metabolic profiles during each respective period. In contrast, samples from different months, particularly between August and November, showed lower correlations, depicted by blue and green areas, indicating significant seasonal variations in metabolite composition. Interestingly, the samples from September and October displayed moderate correlations (yellow and orange areas), suggesting some similarity in their metabolic profiles during these transitional months. Overall, the heatmap effectively captures both the internal consistency of each sampling period and the dynamic shifts in metabolite composition as the seasons progress.

**Figure 2:**
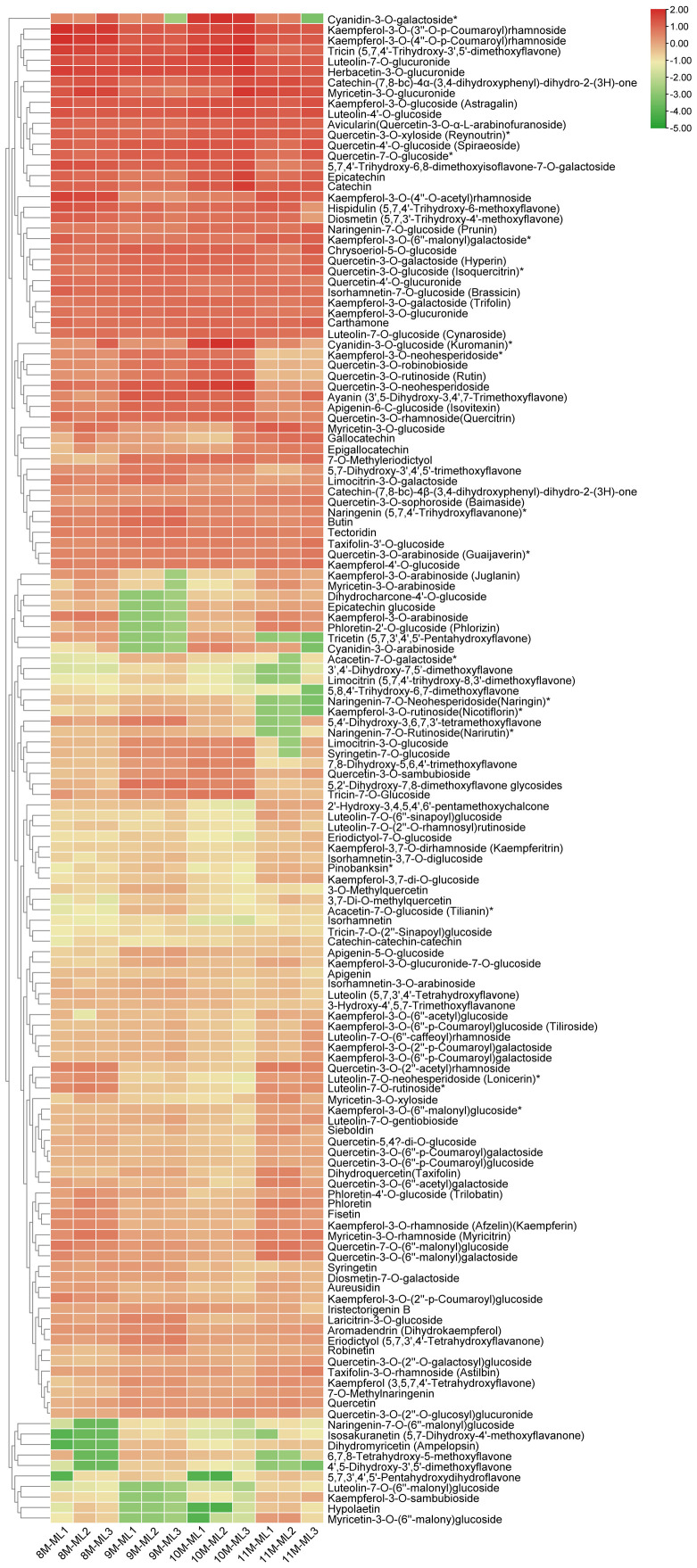
Clustering of 144 flavonoid compounds identified in *C. paliurus* leaves.

**Figure 2** shows the content distribution of 144 flavonoid metabolites in each sample across different groups. The clustering results indicate that the proportion of metabolites increased with the progression of months, with the highest accumulation of flavonoids observed in November. The accumulation of kaempferol and its glycosides was concentrated across the four different harvesting periods. For example, the relative contents of kaempferol-3-O-(3”-O-p-coumaryl) rhamnoside, kaempferol-3-O-(4”-O-p-coumaryl) rhamnoside, and kaempferol-3-O-glucoside (Astragalin) were high across all four harvesting periods (**Figure 2**, **Supplementary Table S1**).

The analysis of flavonoid metabolism in *C. paliurus* leaves revealed significant seasonal variations in the concentrations of flavonoid metabolites from August to November. In August, the concentrations of several flavonoid metabolites peaked. Notably, quercetin-3-O-glucoside and quercetin-3-O-rutinoside (rutin) exhibited high levels, driven by optimal conditions for biosynthesis. These compounds are crucial for antioxidant defense, aiding the plant in coping with oxidative stress. Similarly, kaempferol-3-O-glucoside and kaempferol-3-O-rutinoside were abundant, reflecting their roles in UV protection and antioxidative activities (Lin et al., 2020). As the season transitioned into September, a noticeable decline in some metabolites was observed, for example, kaempferol-3-O-arabinoside, phloretin-2’-O-glucoside and tricetin. The reduced levels of these metabolites suggest a shift in metabolic activity, possibly due to lower environmental stress and the onset of dormancy preparation (Zhang et al., 2020). quercetin-3-O-glucoside (Isoquercitrin) and kaempferol-3-O-glucoside (astragalin) always maintain a high level. By October, the concentrations of these flavonoids continued to increase, reaching a peak. This trend was consistent for quercetin-3-O-neohesperidoside, tricin, as well as tricin-7-O-glucoside. and luteolin-7-O-glucuronide, known for their anti-inflammatory properties, also showed significant reductions from their peak levels in August. The content level of catechin and epicatechin, important for antioxidative protection, remained consistently high, especially in October, reaching a peak. By November, flavonoid concentrations were at their low level, reflecting the plant’s transition to dormancy and significantly slowed metabolic activity. Flavonoids such as quercetin-3-O-rutinoside (rutin), cyanidin-3-O-galactoside and cyanidin-3-O-glucoside showed minimal levels in November, consistent with the overall downregulation of flavonoid biosynthesis. (**Figure 2**, **Supplementary Tables S2**-**S4**). As the season progresses, *C. paliurus* reduces flavonoid synthesis, conserving resources and preparing for dormancy. This adaptive strategy underscores the dynamic regulation of flavonoid metabolism in response to environmental changes, highlighting the ecological and physiological importance of these compounds (Mohammadi et al., 2014; Sun et al., 2024).

### 3.3 Transcriptomics analysis for *C. paliurus* leaves in different months

#### 3.3.1 Screening and analysis of differentially expressed genes

**Figure 3:**
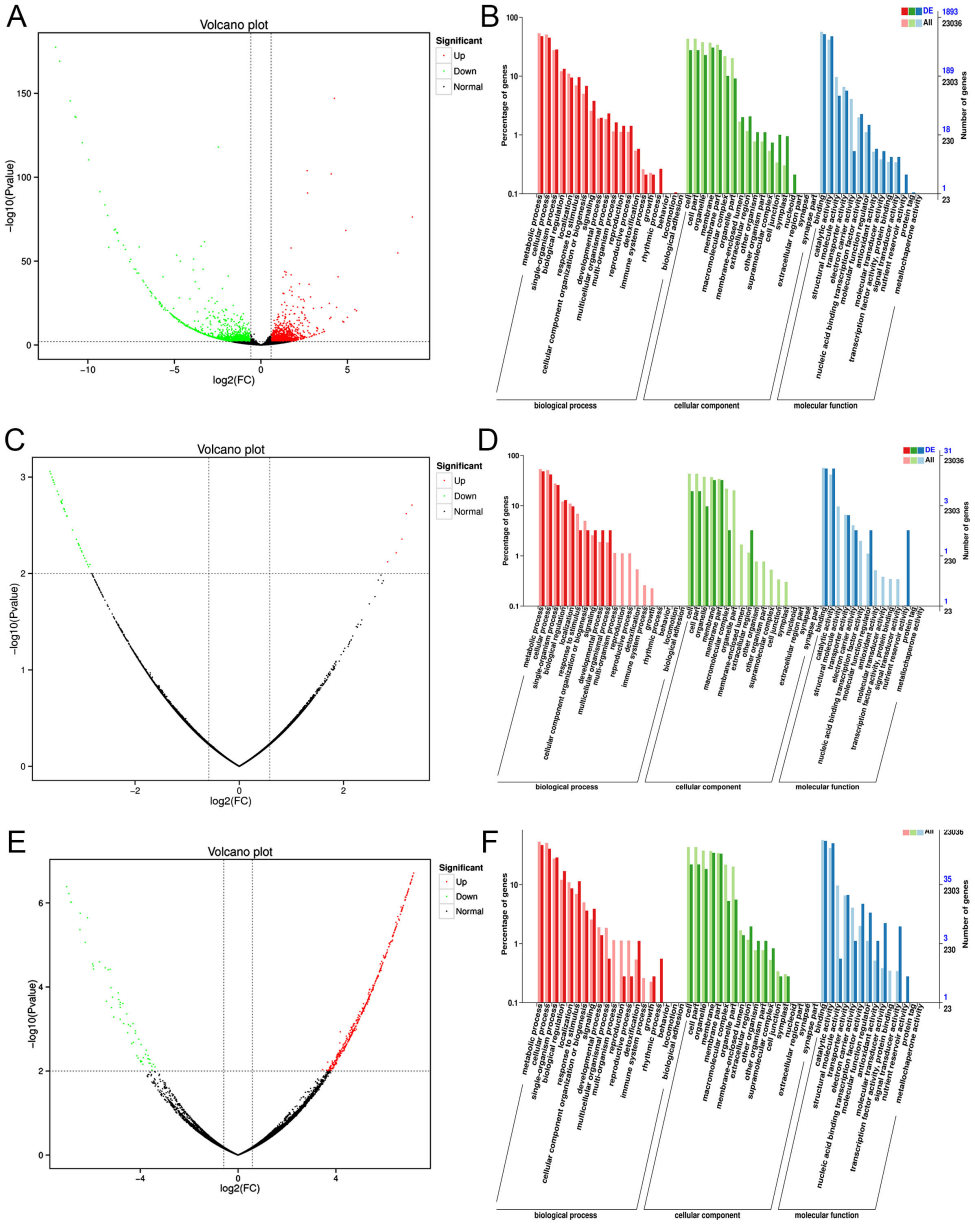
Volcano plots and GO enrichment analysis for differentially expressed genes (*DEGs*) in *C*. *paliurus*. **(A)** Volcano plot of *DEGs* between 8M-ML and 9M-ML samples. **(B)** GO enrichment analysis of *DEGs* between 8M-ML and 9M-ML samples. **(C)** Volcano plot of *DEGs* between 9M-ML and 10M-ML samples. **(D)** GO enrichment analysis of *DEGs* between 9M-ML and 10M-ML samples. **(E)** Volcano plot of *DEGs* between 10M-ML and 11M-ML samples. **(F)** GO enrichment analysis of *DEGs* between 10M-ML and 11M-ML samples.

In the transcriptome data collected across four different harvesting periods from August to November, we focused on the expression analysis of differentially expressed genes (*DEGs*) in three specific comparisons: 8MML_vs_9MML, 9MML_vs_10MML, and 10MML_vs_11MML. DEGs were identified by comparing expression levels between adjacent months using DEGseq software, with the screening criteria set to a p-value of less than 0.01 and a log2 fold change greater than 1.5. The screening results are presented in the form of volcano plots, as shown in **Figures 3A, C, E**. The log2 fold change values on the horizontal axis indicate upregulated genes (greater than 0) and downregulated genes (less than 0), while the vertical axis represents the significance of differential gene expression between the two sample groups. Specifically, in the 8MML_vs_9MML group, we identified 1,226 upregulated and 1,184 downregulated genes (**Figure 3A**). In the 9MML_vs_10MML group, 5 genes were upregulated and 38 genes were downregulated (**Figure 3C**). In the 10MML_vs_11MML comparison, 413 genes were upregulated and 59 genes were downregulated (**Figure 3E**).

Further analysis was performed using Gene Ontology (GO) enrichment for the *DEGs* identified in the 8MML_vs_9MML, 9MML_vs_10MML, and 10MML_vs_11MML comparisons, with the results illustrated in **Figures 3B, D, F**. The GO enrichment analysis highlighted significant biological processes, with most differentially expressed genes (*DEGs*) annotated to processes primarily involved in metabolic and cellular functions. This underscores the essential role of these processes in regulating flavonoid biosynthesis and related metabolic pathways across the different seasonal stages. In the biological process category, *DEGs* were predominantly enriched in metabolic and cellular processes, suggesting that these genes are crucial in regulating various metabolic pathways during the seasonal fluctuations in flavonoid production. The cellular component category showed a high concentration of *DEGs* associated with the cell membrane, particularly in processes related to the formation of cell membrane components. This finding indicates the importance of cellular structure and membrane dynamics in maintaining metabolic functions during the seasonal transitions. Moreover, in the molecular function category, *DEGs* were most enriched in binding and catalytic activities, further emphasizing the role of these genes in driving key biochemical reactions involved in flavonoid biosynthesis. These results not only highlight the key molecular functions associated with flavonoid metabolism but also provide a deeper understanding of the regulatory mechanisms underlying the seasonal changes observed in *C. paliurus*. The GO enrichment analysis thus reveals important insights into the molecular framework governing the plant’s adaptive responses, reinforcing the seasonal modulation of flavonoid biosynthesis.

Subsequently, KEGG pathway enrichment analysis was conducted for the *DEGs* identified in the 8MML_vs_9MML, 9MML_vs_10MML, and 10MML_vs_11MML comparisons, with the results depicted in **Supplementary Figures S1A–C**. In the 8MML_vs_9MML group, a total of 569 *DEGs* were enriched, with a significant concentration in metabolism-related pathways, including plant-pathogen interaction, starch and sucrose metabolism, and notably, 9 *DEGs* related to the flavonoid biosynthesis pathway (**Supplementary Tables S6**), including *CYP90A1*, cinnamoyl-CoA reductase, *VSR6*, Flavonoid 3’-monooxygenase, *CYP714C2, CYP71AN24*, Chalcone and stilbene synthases, *CYP87A3* and *CYP86A22*. In contrast, in the 9MML_vs_10MML group, 38 *DEGs* were enriched, with the majority being related to plant-pathogen interaction, and 2 *DEGs* were annotated to the flavonoid biosynthesis pathway (**Supplementary Tables S7**), including *CYP71D9* and shikimate O-hydroxycinnamoyltransferase. Similarly, in the 10MML_vs_11MML group, 420 *DEGs* were enriched, with a strong emphasis on metabolic and organismal systems pathways, including 4 *DEGs* related to the flavonoid biosynthesis pathway (**Supplementary Tables S8**), including stemmadenine O-acetyltransferase, flavonoid 3’-monooxygenase, Isoflavone reductase and trans-cinnamate 4-monooxygenase.

The comprehensive analysis of *DEGs* in *C. paliurus* leaves across the critical periods from August to November identified numerous genes implicated in both the upstream biosynthesis and downstream metabolism of flavonoids. Key enzymes in the flavonoid biosynthesis pathway, such as Chalcone Synthase (*CHS*), Chalcone Isomerase (*CHI*), and Flavanone 3-Hydroxylase (*F3H*), were prominently upregulated during the transition from August to September, suggesting an early surge in flavonoid production. Additionally, Dihydroflavonol 4-Reductase (*DFR*) and Anthocyanidin Synthase (*ANS*) were significantly upregulated from September to October, correlating with increased production of anthocyanins and other flavonoid derivatives. Notably, Flavonol Synthase (*FLS*), which facilitates the conversion of dihydroflavonols to flavonols, was also upregulated in this period, indicating a diversification of flavonoid types. In the later months, from October to November, genes such as Leucoanthocyanidin Reductase (*LAR*) and Flavonoid 3’-Hydroxylase (*F3’H*) were downregulated, indicating a decrease in the synthesis of proanthocyanidins and other flavonoid metabolites as the leaves transitioned into winter. Additionally, genes involved in the regulation and modification of flavonoids, including UDP-glucose-3-O-glucosyltransferase (*UFGT*) and Cytochrome P450 enzymes like *CYP75B1*, which are critical for the hydroxylation and glycosylation of flavonoids, exhibited varying expression patterns, underscoring the complex regulation of flavonoid metabolism. This dynamic expression of flavonoid-related genes highlights the intricate balance between biosynthesis and metabolism that is modulated in response to seasonal changes, offering valuable insights into the optimal harvesting period and potential genetic targets for enhancing flavonoid content in *C. paliurus* leaves (**Supplementary Tables S6**-**S8**).

#### 3.3.2 Integrated weighted gene co-expression network analysis of transcriptomic and metabolomic data

**Figure 4:**
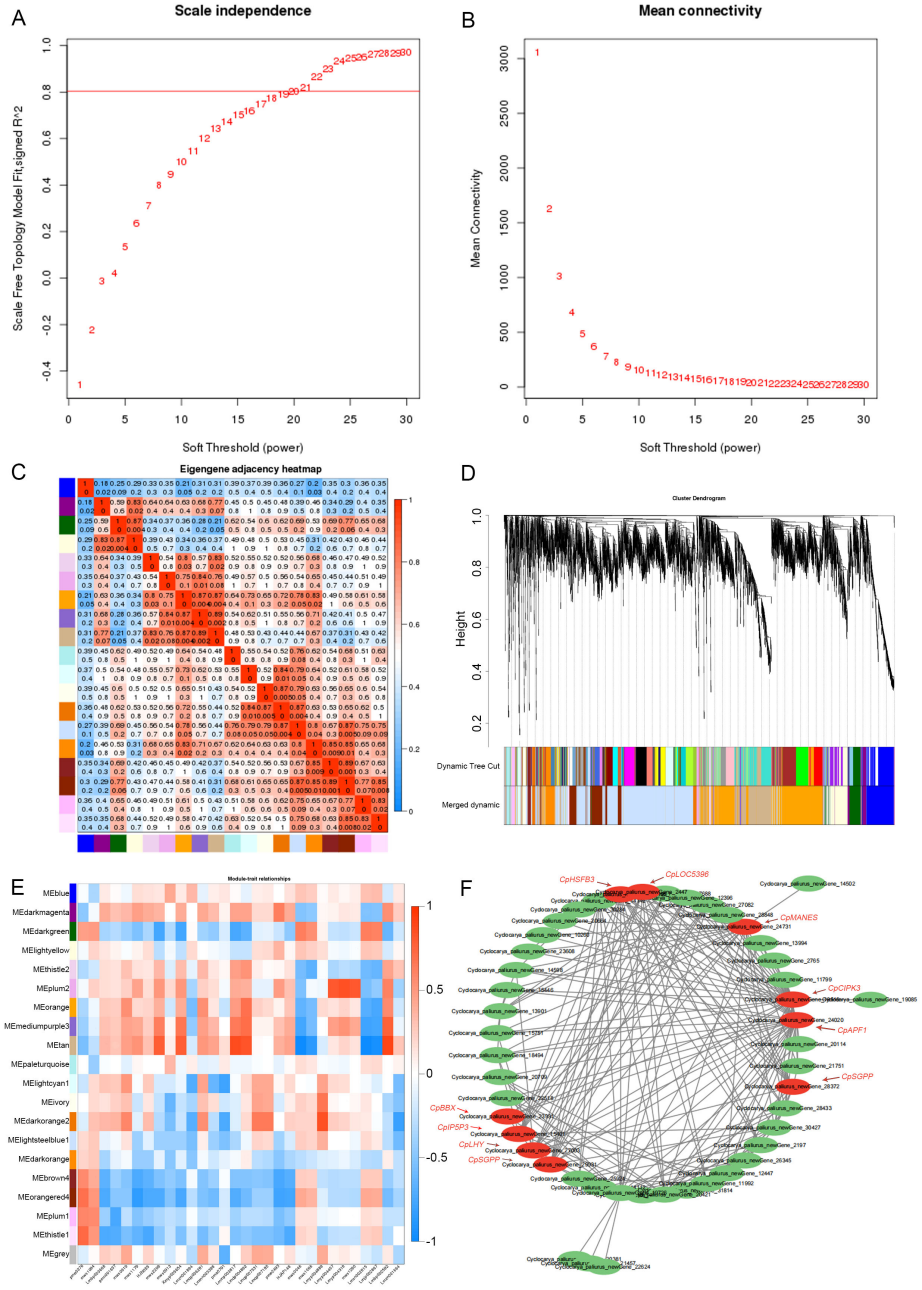
Gene co-expression network analysis. **(A)** Scale independence as a function of soft-thresholding power. **(B)** Mean connectivity across soft-thresholding powers. **(C)** Heatmap showing correlations between gene expression modules. **(D)** Clustering dendrogram of expressed genes, with modules indicated by different colors. **(E)** Heatmap illustrating correlations between gene modules and various traits. **(F)** Co-expression network of genes in a selected module, highlighting hub genes.

To analyze gene-metabolite correlations related to flavonoid biosynthesis, we conducted Weighted Gene Co-expression Network Analysis (WGCNA) using flavonoid-related metabolites and transcriptomic data from *C. paliurus* leaves collected between August and November. We selected an optimal soft threshold of β=19, ensuring a scale-free network with a fitting index above 0.8 (Langfelder and Horvath, 2008). This threshold allowed us to construct a robust co-expression network (**Figures 4A, B**).

We identified 20 distinct gene modules, each visualized by a unique color in **Figure 4C**. Highly correlated genes were grouped within the same module, while the gray module contained genes without significant correlation patterns. The lightsteelblue1 module had the most genes (2,479), while the thistle1 module had the fewest (41). **Figure 4C** illustrates the correlations between modules, with blue shades indicating weaker correlations and red indicating stronger ones. As shown in **Figures 4D, E**, we examined correlations between gene modules and specific flavonoids detected in the metabolomic analysis, including compounds like naringin, geranolin, kaempferol-3-O-arabinoside, kaempferol-3-O-rhamnoside, and naringin-7-O-glucoside. The tan module demonstrated a high correlation (correlation coefficient > 0.6) with key flavonoids, such as kaempferol-3-O-glucoside, kaempferol-4’-O-glucoside, and kaempferol-3,7-di-O-glucoside. Other modules, including thistle1 and plum2, were associated with naringenin and specific kaempferol derivatives, such as kaempferol-3-O-(2”-p-coumaryl) galactoside and kaempferol-3-O-(6”-p-coumaryl) galactoside (Xie et al., 2015).

To further investigate regulatory genes, we constructed an interaction network of genes within the tan module using Cytoscape, focusing on the top 200 genes with the highest co-expression weights. As visualized in **Figure 4F**, we identified ten key genes, *CpBBX* (newGene_23931), *CpIP5P3* (newGene_15401), *CpLHY* (newGene_27003), *CpSGPP* (newGene_29931), *CpCIPK3* (newGene_19518), *CpAPF1* (newGene_24020), *CpHSFB3* (newGene_10195), *CpLOC5396* (newGene_2447), *CpMANES* (newGene_24731), and *CpLOC7593* (newGene_28372), as central regulators within this network (Shannon et al., 2003). Their high connectivity suggests pivotal roles in regulating flavonoid biosynthesis. For example, Cyclocarya_paliurus_newGene_27003 and newGene_29931 were notably active in October, with corresponding upregulation of metabolites like quercetin-3-O-(2”-acetyl) rhamnoside and luteinin-7-O-rutinoside in November. This pattern suggests that these genes may contribute to the synthesis of flavonoids as seasonal conditions change.

#### 3.3.3 Identification of key genes in the flavonoid biosynthesis pathway

**Figure 5:**
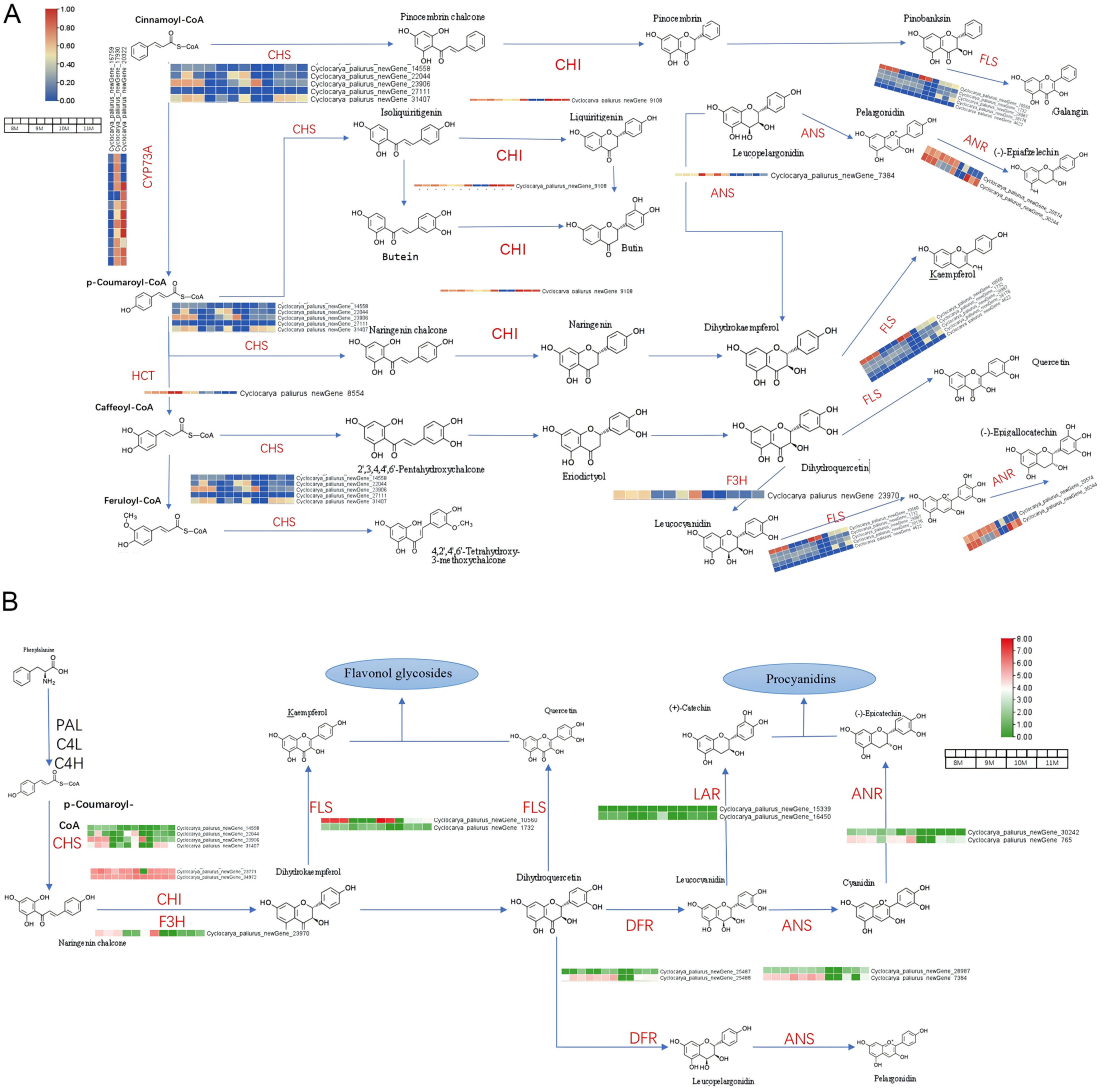
Transcriptional expression profiles of key functional genes involved in flavonoid biosynthesis. **(A)** Expression profiles of genes involved in the transformation from precursor molecules to flavonoids. **(B)** Expression profiles of genes responsible for flavonoid biosynthesis.

Comprehensive differential expression analysis revealed several key genes involved in the flavonoid biosynthesis pathway in *C. paliurus*, with notable seasonal variations across the studied months. Chalcone Synthase (*CHS*), a critical enzyme responsible for initiating flavonoid biosynthesis, exhibited peak expression in August, aligning with the highest flavonoid content observed during this period. This suggests that *CHS* serves as a major regulatory enzyme driving flavonoid production in response to favorable environmental conditions. Similarly, Chalcone Isomerase (*CHI*), which converts naringenin chalcone to naringenin, displayed elevated expression in August and September, ensuring an adequate supply of naringenin as a precursor for downstream flavonoid biosynthesis (Wan et al., 2024). Flavanone 3-Hydroxylase *(F3’H*), critical for diversifying flavonoid structures, peaked in September, while Dihydroflavonol 4-Reductase (*DFR*) showed heightened expression in both August and September, supporting anthocyanin biosynthesis during this period. Flavonol Synthase (*FLS*), which catalyzes the conversion of dihydroflavonols into flavonols such as quercetin and kaempferol, also exhibited peak expression in August, coinciding with the accumulation of these antioxidant compounds (**Figures 5A, B**) (Jaakola, 2013).

**Figure 5A** illustrates the flavonoid biosynthetic pathway in *C. paliurus*, mapping the transformation of precursors like cinnamoyl-CoA and p-coumaroyl-CoA into diverse flavonoids through the activity of key enzymes, including *CHS, CHI, F3’H, FLS*, Anthocyanidin Synthase (*ANS*), and Anthocyanidin Reductase (*ANR*) (Winkel-Shirley, 2001; Hichri et al., 2010). The heat maps integrated into this pathway visually represent the differential expression of these genes, with color gradients highlighting the upregulation and downregulation patterns in response to developmental and environmental stimuli (Falcone Ferreyra et al., 2012). These expression patterns underline the dynamic regulation of flavonoid biosynthesis, driven by the plant’s adaptive responses to oxidative stress, UV radiation, and other environmental challenges, while simultaneously contributing to human health benefits through antioxidant and anti-inflammatory properties (Wang et al., 2024). Genes associated with the biosynthesis of procyanidins, such as Leucoanthocyanidin Reductase (*LAR*), *DFR, ANS*, and *ANR*, were also dynamically expressed. These enzymes play essential roles in synthesizing catechins and epicatechins, as shown in **Figure 5B**, which emphasizes the significance of flavonol glycosides and procyanidins as key flavonoid classes with diverse biological functions (Xie et al., 2015; Xu et al., 2024). Notably, the heat maps reveal seasonal changes in gene expression, with green and red denoting down- and upregulation, respectively, reflecting the plant capacity to modulate flavonoid synthesis in response to environmental or developmental cues (Hichri et al., 2010).

From August to November, dynamic shifts in the expression of flavonoid-related genes were observed. Genes such as CHS, CHI, and F3’H exhibited prominent upregulation during the transition from August to September, corresponding to an early surge in flavonoid production. In September and October, elevated expression levels of *DFR* and *ANS* coincided with increased production of anthocyanins, while FLS activity diversified flavonoid profiles by facilitating the synthesis of flavonols. By late autumn (October to November), genes such as LAR and F3’H were downregulated, marking a decline in proanthocyanidin synthesis as the plant transitioned into dormancy. Additionally, genes involved in flavonoid regulation and modification, including UDP-glucose-3-O-glucosyltransferase (*UFGT*) and Cytochrome P450 enzymes like *CYP75B1*, exhibited fluctuating expression patterns, underscoring the complexity of flavonoid metabolism.

The interaction of 57 genes linked to 16 enzymes, as noted in the KEGG pathway (ko00941), highlights the extensive regulatory control underlying flavonoid biosynthesis. These regulatory networks enable *C. paliurus* to adapt to seasonal changes, bolstering its resilience to environmental stressors while optimizing flavonoid production for both plant defense and human health benefits (Falcone Ferreyra et al., 2012). The findings presented in **Figures 5A, B**; **Supplementary Tables S6**-**S8** not only emphasize the intricate balance between flavonoid biosynthesis and metabolism but also provide valuable insights for optimizing the harvesting period and identifying potential genetic targets for enhancing flavonoid content in *C. paliurus*.

#### 3.3.4 Analysis of gene-flavonoid relationships and their seasonal variations

**Figure 6:**
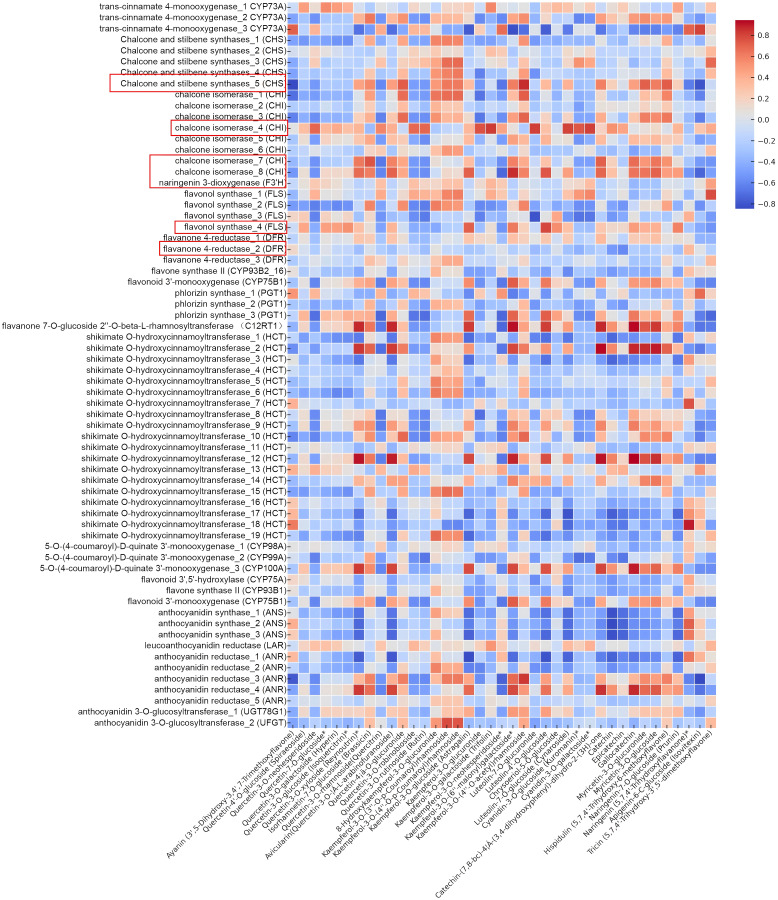
Heatmap showing correlations between functional genes and flavonoids in *C. paliurus*.

**Figure 7:**
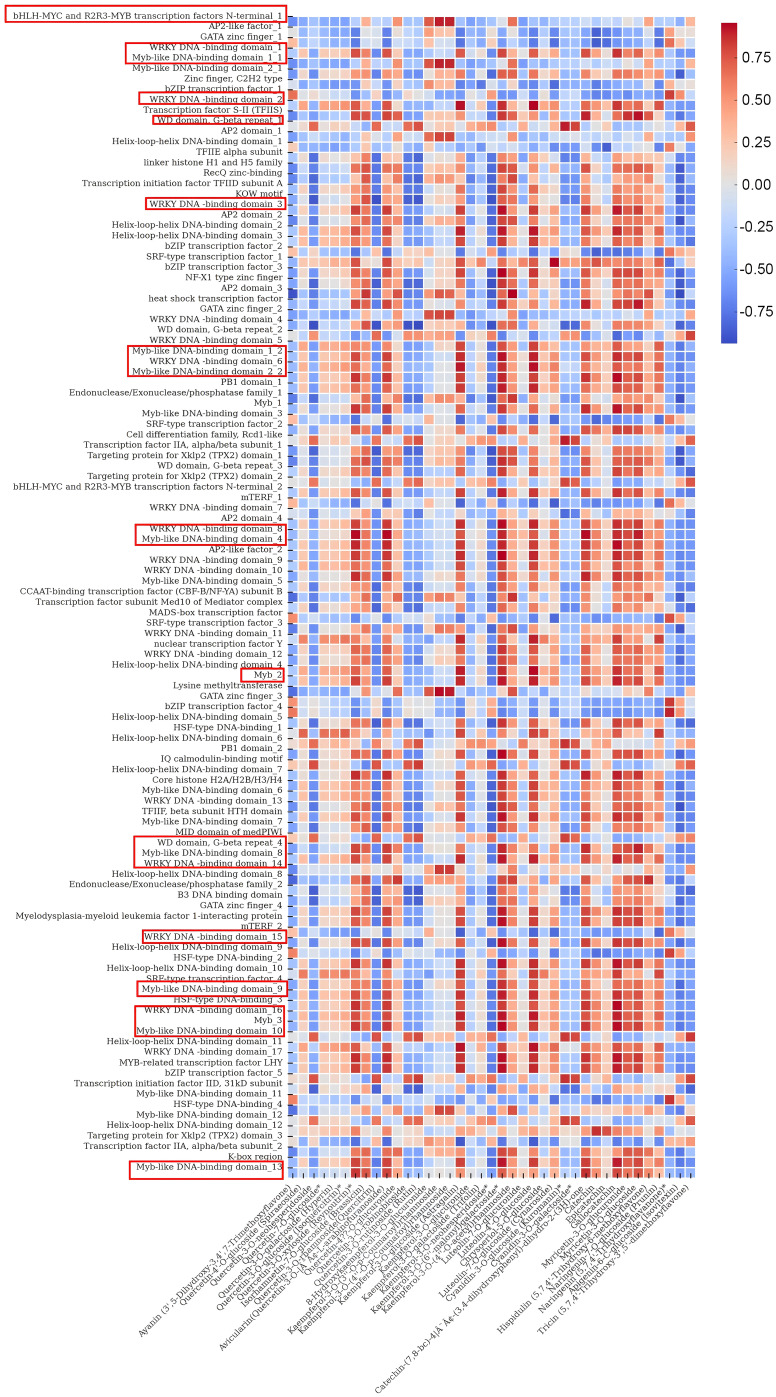
Heatmap illustrating the correlations between transcription factors and flavonoids in *C. paliurus.*.

The total number of flavonoid substances in the leaves of *C. paliurus* was 144, among which 39 major flavonoid substances with high content accounted for more than 80% of the total flavonoid content from August to November (**Table 1**). Therefore, we selected these 39 flavonoid substances for the analysis of gene-flavonoid relationships and seasonal variations, Pearson correlation studies were carried out between them and the function genes of the flavonoid synthesis pathway (**Supplementary Table S9**) and the significantly different transcription factors in *DEGs* (**Supplementary Table S10**). The regulation of flavonoid biosynthesis in *C. paliurus* was thoroughly examined through a comprehensive Pearson correlation analysis, which linked the expression of functional biosynthetic genes and regulatory transcription factors to seasonal variations in flavonoid content. The data from **Figures 6**, **7** provide critical insights into how these genes exhibit coordinated fluctuations across different months, significantly influencing the production of key flavonoids such as quercetin and kaempferol glycosides. This seasonal regulation sheds light on the plants intricate molecular responses to environmental stressors and climatic changes (**Supplementary Tables S11**-**S12**).

**Table 1:** The content of the main flavonoids in *C. paliuru* leaves.

Compounds	8M		Ratio	9M		Ratio	10M		Ratio		11M		Ratio
	Average	STDEV	(%)	Average	STDEV	(%)	Average	STDEV	(%)	Average		STDEV	(%)
Ayanin (3',5-Dihydroxy-3,4',7-Trimethoxyflavone)	7.56E+05	3.27E+05	0.22	7.87E+06	3.61E+05	3.95	5.63E+06	5.71E+05	1.33	2.41E+06		2.77E+06	0.59
Quercetin-4'-O-glucoside (Spiraeoside)	5.43E+06	5.85E+05	1.55	7.58E+06	5.28E+05	3.81	9.98E+06	1.68E+06	2.36	9.62E+06		5.92E+06	2.35
Quercetin-3-O-neohesperidoside	2.77E+06	2.82E+05	0.79	7.03E+06	1.37E+06	3.53	2.30E+07	3.10E+06	5.44	4.28E+05		4.21E+04	0.1
Quercetin-7-O-glucoside*	4.40E+06	4.58E+05	1.26	6.10E+06	4.26E+05	3.06	9.16E+06	1.97E+06	2.17	8.36E+06		5.63E+06	2.04
Quercetin-3-O-galactoside (Hyperin)	2.79E+06	3.35E+05	0.8	3.38E+06	2.96E+05	1.7	4.92E+06	5.83E+05	1.16	4.50E+06		2.06E+06	1.1
Quercetin-3-O-glucoside (Isoquercitrin)*	2.37E+06	3.96E+05	0.68	3.35E+06	1.93E+05	1.68	4.44E+06	7.11E+05	1.05	4.56E+06		2.49E+06	1.12
Quercetin-3-O-xyloside (Reynoutrin)*	5.68E+06	2.02E+05	1.62	3.31E+06	4.17E+05	1.66	7.63E+06	4.39E+05	1.81	1.37E+07		4.64E+06	3.34
Isorhamnetin-7-O-glucoside (Brassicin)	4.24E+06	6.92E+05	1.21	3.14E+06	2.12E+05	1.58	2.63E+06	3.68E+05	0.62	4.47E+06		1.18E+06	1.09
Quercetin-3-O-rhamnoside(Quercitrin)	2.46E+06	9.32E+05	0.7	3.01E+06	2.96E+05	1.51	5.55E+06	4.61E+05	1.31	1.23E+06		4.01E+05	0.3
Avicularin(Quercetin-3-O-α-L-arabinofuranoside)	4.88E+06	2.32E+05	1.4	2.88E+06	2.70E+05	1.45	6.58E+06	3.12E+05	1.56	1.18E+07		3.67E+06	2.89
Quercetin-4′-O-glucuronide	3.30E+06	1.42E+05	0.94	2.42E+06	1.28E+05	1.22	2.51E+06	1.19E+05	0.59	3.04E+06		8.90E+05	0.74
Quercetin-3-O-robinobioside	7.59E+05	1.08E+05	0.22	1.81E+06	7.09E+05	0.91	5.72E+06	8.31E+05	1.35	1.07E+05		1.45E+04	0.03
Quercetin-3-O-rutinoside (Rutin)	5.99E+05	6.71E+04	0.17	1.38E+06	3.94E+05	0.69	5.11E+06	1.25E+06	1.21	7.71E+04		1.23E+04	0.02
8-Hydroxykaempferol(Herbacetin)-3-O-glucuronide	2.31E+07	1.68E+06	6.59	1.84E+07	1.13E+06	9.23	1.80E+07	7.00E+05	4.27	1.76E+07		4.14E+06	4.29
Kaempferol-3-O-(3''-O-p-Coumaroyl)rhamnoside	4.27E+07	2.90E+06	12.2	8.38E+06	1.72E+06	4.21	2.01E+07	8.26E+05	4.76	1.96E+07		5.19E+06	4.79
Kaempferol-3-O-(4''-O-p-Coumaroyl)rhamnoside	3.93E+07	7.97E+05	11.21	8.25E+06	1.77E+06	4.14	2.00E+07	1.03E+06	4.73	1.82E+07		5.01E+06	4.46
Kaempferol-3-O-glucoside (Astragalin)	8.53E+06	9.96E+05	2.44	7.17E+06	2.63E+05	3.6	1.13E+07	1.95E+06	2.67	1.76E+07		2.27E+06	4.31
Kaempferol-3-O-glucuronide	2.44E+06	3.76E+05	0.7	2.89E+06	4.39E+05	1.45	3.60E+06	5.53E+04	0.85	2.78E+06		2.99E+05	0.68
Kaempferol-3-O-galactoside (Trifolin)	2.37E+06	5.15E+05	0.68	2.47E+06	2.72E+05	1.24	4.32E+06	8.49E+05	1.02	3.25E+06		6.03E+05	0.79
Kaempferol-3-O-neohesperidoside*	7.94E+05	1.45E+05	0.23	2.06E+06	1.84E+05	1.03	2.92E+06	3.22E+05	0.69	4.99E+04		2.48E+04	0.01
Kaempferol-3-O-(6''-malonyl)galactoside*	4.40E+06	9.64E+05	1.26	1.41E+06	1.25E+05	0.71	2.53E+06	4.84E+05	0.6	1.05E+07		1.74E+06	2.57
Kaempferol-3-O-(4''-O-acetyl)rhamnoside	1.96E+07	1.52E+06	5.59	2.88E+05	3.55E+04	0.14	1.84E+06	3.34E+05	0.44	1.60E+07		6.57E+06	3.91
Luteolin-7-O-glucuronide	1.26E+07	2.34E+05	3.6	1.51E+07	1.26E+06	7.59	1.95E+07	1.65E+06	4.62	1.66E+07		5.28E+06	4.06
Luteolin-4'-O-glucoside	9.03E+06	7.74E+05	2.58	7.62E+06	4.12E+05	3.83	1.15E+07	1.37E+06	2.73	1.88E+07		2.90E+06	4.6
Chrysoeriol-5-O-glucoside	3.11E+06	1.95E+05	0.89	5.63E+06	3.43E+05	2.83	5.39E+06	6.47E+05	1.28	6.23E+06		5.69E+06	1.52
Luteolin-7-O-glucoside (Cynaroside)	3.24E+06	6.37E+05	0.93	2.30E+06	4.65E+05	1.15	4.93E+06	9.97E+05	1.17	5.06E+06		6.13E+05	1.24
Cyanidin-3-O-glucoside (Kuromanin)*	2.69E+06	3.62E+06	0.77	3.60E+05	1.24E+05	0.18	2.93E+07	1.76E+07	6.93	8.75E+05		5.97E+05	0.21
Cyanidin-3-O-galactoside*	3.81E+06	5.06E+06	1.09	3.92E+05	3.66E+05	0.2	3.54E+07	1.29E+07	8.38	1.10E+06		9.64E+05	0.27
Catechin-(7,8-bc)-4α-(3,4-dihydroxyphenyl)-dihydro-2-(3H)-one	1.05E+07	3.07E+06	3	2.91E+06	4.70E+05	1.46	8.22E+06	2.29E+06	1.95	2.98E+07		1.61E+07	7.28
Catechin	5.64E+06	9.66E+05	1.61	1.94E+06	1.44E+05	0.98	1.22E+07	5.02E+06	2.88	1.30E+07		3.53E+06	3.17
Epicatechin	4.69E+06	3.52E+05	1.34	1.16E+06	1.48E+05	0.58	9.35E+06	4.10E+06	2.21	6.91E+06		2.97E+06	1.69
Gallocatechin	8.70E+05	9.50E+05	0.25	1.40E+05	7.32E+04	0.07	6.18E+05	9.08E+05	0.15	4.94E+06		1.70E+06	1.21
Myricetin-3-O-glucuronide	1.97E+07	1.03E+07	5.63	2.34E+06	1.17E+06	1.17	1.03E+07	1.07E+07	2.44	3.95E+07		1.78E+07	9.65
Myricetin-3-O-glucoside	2.50E+06	1.66E+06	0.71	2.08E+05	6.68E+04	0.1	9.43E+05	1.11E+06	0.22	1.09E+07		6.90E+06	2.66
Hispidulin (5,7,4'-Trihydroxy-6-methoxyflavone)	9.73E+06	1.92E+06	2.78	2.70E+06	1.20E+06	1.36	4.32E+06	4.31E+04	1.02	9.04E+06		7.37E+06	2.21
Naringenin-7-O-glucoside (Prunin)	2.02E+06	1.56E+05	0.58	7.79E+05	1.15E+05	0.39	3.80E+06	1.94E+05	0.9	5.20E+06		2.50E+06	1.27
Naringenin (5,7,4'-Trihydroxyflavanone)*	1.14E+06	7.91E+04	0.32	2.24E+06	4.57E+05	1.13	1.20E+06	2.73E+04	0.28	9.43E+05		2.66E+05	0.23
Apigenin-6-C-glucoside (Isovitexin)	1.14E+06	2.55E+05	0.33	3.56E+06	3.99E+05	1.79	3.97E+06	2.69E+05	0.94	6.68E+05		2.29E+05	0.16
Tricin (5,7,4'-Trihydroxy-3',5'-dimethoxyflavone)	2.06E+07	3.35E+06	5.89	9.23E+06	1.55E+06	4.64	2.65E+07	2.62E+06	6.28	6.07E+06		8.91E+05	1.48
**Total Ratio (%)**			**84.76**			**81.95**			**86.37**				**84.43**

Beginning with the analysis of functional genes as illustrated in **Figure 6**, it is evident that several core biosynthetic genes play pivotal roles in driving the flavonoid biosynthesis pathway. Among these, chalcone synthase (*CHS*) stands out as the key enzyme catalyzing the initial step in flavonoid biosynthesis, converting malonyl-CoA and p-coumaroyl-CoA into chalcones. The strong positive correlation between *CHS* expression and flavonoid accumulation, particularly in late summer, suggests that this gene is crucial for the overall regulation of flavonoid biosynthesis in *C. paliurus*. This observation is consistent with previous studies conducted in white apruce, where it was revealed that accumulation of *CHS* mRNA in needle tissue following mechanical wounding, or application of signal molecules, such as jasmonic acid or methyl jasmonate (Richard et al., 2000).

Similarly, the role of chalcone isomerase (*CHI*), which converts chalcones into flavanones, is highlighted by its high expression during the peak flavonoid accumulation period. This gene plays a vital role in facilitating downstream biosynthesis, particularly in the synthesis of flavonols. The expression patterns observed in *C. paliurus* align with findings in *Glycine max*, where *CHI* activity increased under environmental stress, enabling the plant to boost flavonoid production as a means of stress adaptation (Seehaus and Tenhaken, 1998). Furthermore, flavanone 3’-hydroxylase (*F3’H*), which catalyzes the conversion of flavanones into dihydroflavonols, demonstrated a strong correlation with both quercetin and kaempferol derivatives. This gene is integral to the overall balance of flavonol types produced, and its seasonal expression pattern mirrors those observed in other species such as *Malus domestica*, where *F3’H* plays a key role in regulating flavonoid biosynthesis during fruit development (Torres et al., 2020).

Moving further down the biosynthetic pathway, dihydroflavonol 4-reductase (*DFR*) exhibited significant correlations with anthocyanin content, particularly with cyanidin-3-O-glucoside. This suggests that *DFR* plays a vital role in anthocyanin biosynthesis during the late summer months, likely as part of the plant’s adaptive response to high light conditions. Such findings are corroborated by studies in *Vitis vinifera* (grape), where ubiquitin ligase *VvWRKY24* could directly interact with the promoters of dihydroflavonol-4-reductase (*DFR*) to inhibit proanthocyanidins biosynthesis (Zhao et al., 2024). In parallel, flavonol synthase (*FLS*), which catalyzes the final conversion of dihydroflavonols into flavonols such as quercetin and kaempferol, also demonstrated strong seasonal expression patterns, correlating with the high levels of flavonols observed in late summer. This observation is consistent with research on *Arabidopsis*, where *FLS* was shown to be upregulated in response to environmental stimuli, enhancing the synthesis of flavonols for drought inducing (Ma et al., 2024).

Turning to the analysis of transcription factors in **Figure 7**, it is apparent that the regulatory machinery governing flavonoid biosynthesis is equally complex and dynamic. Members of the *MYB* family, including *MYB11, MYB12*, and *MYB111*, exhibited strong positive correlations with quercetin and kaempferol glycosides, indicating their central roles in regulating flavonol synthesis. These transcription factors have been widely documented for their involvement in controlling flavonoid biosynthesis, particularly in response to environmental stresses such as UV radiation. In *Arabidopsis thaliana*, for instance, similar *MYB* transcription factors have been shown to directly regulate the expression of flavonol biosynthetic genes, enhancing flavonol accumulation under high-light conditions (Stracke et al., 2007).

*MYB75* (*PAP1*) and *MYB90* (*PAP2*) were closely associated with anthocyanin production, particularly cyanidin-3-O-glucoside. These transcription factors are well-known for their roles in regulating anthocyanin biosynthesis in a wide range of plant species. For example, in *Vitis vinifera*, in ‘Malbec’ grape skins, *VviMyb4a* and *VviMyb4*b likely regulate the early genes in the anthocyanin biosynthesis pathway, while *VviMybC2-L2* and *VviMyb4-lik*e are co-expressed at later stages of the process. Additionally, *VviMybC2-L1* and *VviMybC2-L3* are activated during two distinct phases of berry development. Their initial induction occurs towards the end of the green stage, suggesting a potential cooperative role in repressing proanthocyanidin synthesis. These genes are then reactivated during maturation, potentially inhibiting anthocyanin production at this later stage (Muñoz et al., 2019). Furthermore, the role of *bHLH* and *WD40* transcription factors, which often act as co-regulators in the *MYB-bHLH-WD40* complex, is also highlighted in this study. *TT8*, a member of the *bHLH* family, was strongly correlated with proanthocyanidin accumulation, further supporting its function in regulating later stages of the flavonoid biosynthetic pathway, particularly in response to seasonal changes. Studies in *Arabidopsis* have shown that *TT8* plays a critical role in regulating seed coat pigmentation and proanthocyanidin biosynthesis, further validating its importance in flavonoid biosynthesis (Baudry et al., 2004). Another significant finding is the negative correlation between *MYB4* and flavonoid content, particularly in late summer. This suggests that *MYB4* acts as a repressor of flavonoid biosynthesis, likely through the downregulation of *CHS* expression to prevent overaccumulation of flavonoids under high-light conditions. This regulatory function has been well-documented in other species, such as *Arabidopsis*, where *MYB4* negatively regulates flavonoid biosynthesis by repressing the expression of early biosynthetic genes (Jin et al., 2000).

Additionally, transcription factors such as *WRKY33* and *NAC* demonstrated significant correlations with flavonoid levels, particularly kaempferol glycosides. *WRKY33* is known to play a critical role in regulating plant responses to biotic and abiotic stresses, and its positive correlation with flavonoid levels suggests that it may upregulate flavonoid biosynthesis as part of the plant’s defense mechanisms. Studies in *Oryza sativa* have similarly highlighted the role of *WRKY33* in enhancing flavonoid production under stress conditions, further supporting its function in *C. paliurus* (Jan et al., 2022). The correlation between *EIN3, ABI5*, and anthocyanin content suggests that hormonal signaling pathways, particularly ethylene and abscisic acid (ABA), are also involved in the regulation of flavonoid biosynthesis in *C. paliurus*. The positive correlations between these transcription factors and flavonoid content in late summer and early autumn indicate that they may play a role in modulating the plant transition to dormancy by regulating flavonoid production. Similar findings have been reported in *Glycine max*, where ethylene and ABA signaling pathways were shown to regulate flavonoid accumulation in response to environmental stresses (Jan et al., 2022).

## 4 Discussion

Our study on the seasonal dynamics of flavonoid biosynthesis in *C. paliurus* provides several new insights that advance the understanding of secondary metabolism in this species and contribute to the broader field of plant biology. While previous studies have documented the presence and seasonal variation of flavonoids in other species, our research uniquely combines metabolomic and transcriptomic analyses to elucidate the molecular mechanisms underlying these fluctuations in *C. paliurus*. This comprehensive approach has allowed us to identify not only the seasonal patterns in flavonoid accumulation but also the regulatory networks that drive these changes, thus offering a more complete picture of the plant’s adaptive responses. The regulation of flavonoid biosynthesis in *C. paliurus* is intricately controlled by transcription factors, particularly those belonging to the *MYB* and *bHLH* families. These transcription factors are known to interact synergistically, often forming the *MYB-bHLH-WD40* (*MBW*) complex, which plays a crucial role in activating the expression of key structural genes in the flavonoid biosynthetic pathway, such as *DFR* and *ANS* (Xu et al., 2015; Hichri et al., 2010). The *MYB* factors provide specificity to the target genes, while *bHLH* factors enhance the transcriptional activation, ensuring robust flavonoid production in response to environmental and developmental cues. In our study, the upregulation of specific *MYB* and *bHLH* genes during periods of high flavonoid accumulation suggests that these transcription factors are central to the plant adaptive response to seasonal stressors, such as increased UV radiation and oxidative stress in late summer (Dubos et al., 2010). This regulatory mechanism is consistent with findings in other plant species, where the *MYB-bHLH* interaction has been shown to be critical for the production of flavonoids, anthocyanins, and other secondary metabolites. Understanding the specific roles of these transcription factors not only provides insight into the molecular control of flavonoid biosynthesis in *C. paliurus* but also highlights potential targets for genetic manipulation to enhance flavonoid content for agricultural and medicinal purposes.

Understanding the specific roles of these transcription factors not only provides insight into the molecular control of flavonoid biosynthesis in *C. paliurus* but also highlights potential targets for genetic manipulation to enhance flavonoid content for agricultural and medicinal purposes. In line with this, recent studies on *C. paliurus* have contributed to the growing body of knowledge on flavonoid regulation. For instance, the study by Caowen et al. (2023) explored the regulation of flavonoid biosynthesis for leaf coloring, identifying key genes involved in response to environmental factors. This study complements our findings by further illustrating how flavonoid biosynthesis is influenced by environmental conditions such as UV stress, highlighting its adaptive role in *C. paliurus* (Caowen et al., 2023). The whole-genome duplication event in *C. paliurus* also plays a significant role in its adaptive evolution, impacting the regulatory networks governing flavonoid biosynthesis. As Qu et al. (2023) noted, the duplication reshaped the genome, enhancing the plant ability to produce secondary metabolites like flavonoids. This genomic evidence supports our findings, suggesting that genetic evolution has contributed to the plant ability to modulate flavonoid production in response to seasonal changes (Qu et al., 2023). Environmental conditions and genetic similarity were shown to jointly influence the flavonoid variation patterns in *C. paliurus* leaves, as demonstrated by Sun et al. (2024). Their study confirmed that both genetic factors and environmental conditions contribute to flavonoid variation, and their findings align with our results, which highlight the importance of seasonal fluctuations in flavonoid accumulation (Sun et al., 2024).

An innovative aspect of our study is the application of co-expression network analysis to link specific gene modules with the biosynthesis of flavonoid compounds. This approach, which has been used in other plant systems such as *Arabidopsis thaliana*, has allowed us to identify gene networks that are strongly associated with the production of key flavonoids like kaempferol derivatives (Soubeyrand et al., 2021; Langfelder and Horvath, 2008). The tan module, in particular, was found to be correlated with the biosynthesis of these compounds, suggesting that targeted manipulation of this module could enhance flavonoid production in *C. paliurus*. The strong correlations observed between the expression of specific genes and the seasonal variation in flavonoid content in *C. paliurus* highlight the pivotal roles these genes play in the biosynthesis and regulation of flavonoids. The genes identified, particularly those coding for chalcone synthase (*CHS*), cytochrome P450 (*CYP73A100*), and other enzymes involved in flavonoid modification, demonstrate consistent expression patterns that align with the seasonal peaks in flavonoid accumulation. This regularity suggests that these genes are not only integral to the biosynthesis of key flavonoids but also responsive to environmental cues that influence their production. As such, these genes represent valuable targets for molecular breeding programs aimed at enhancing flavonoid content in *C. paliurus*, particularly for the development of varieties with improved medicinal and nutritional properties. By leveraging these genes as molecular markers, it may be possible to select and cultivate *C. paliurus* plants that consistently produce high levels of beneficial flavonoids across different growing seasons, thereby optimizing their potential use in nutraceutical and therapeutic applications. This finding not only advances our understanding of the genetic regulation of flavonoid biosynthesis but also opens up new possibilities for breeding or engineering plants with enhanced levels of these valuable metabolites. The broader implications of our findings are significant for both basic plant science and applied research. The detailed characterization of flavonoid biosynthesis in *C. paliurus* provides a valuable resource for the development of functional foods and medicinal products. Flavonoids are well-known for their health-promoting properties, including antioxidant, anti-inflammatory, and anticancer activities (Gould and Lister, 2006). By identifying the specific genes and regulatory networks that control flavonoid production, our research lays the groundwork for future efforts to enhance these compounds in *C. paliurus* and other economically important plants. Additionally, understanding the seasonal dynamics of flavonoid production could inform optimal harvesting strategies to maximize the yield of these bioactive compounds.

Despite the significant contributions of our study, there are several areas that warrant further investigation. For instance, while we have identified key regulatory genes involved in flavonoid biosynthesis, the specific environmental triggers that modulate their expression remain to be fully understood. Future research could explore the roles of factors such as light intensity, temperature, and water availability in regulating these biosynthetic pathways (Falcone Ferreyra et al., 2012). Moreover, it would be beneficial to extend this research to other parts of the *C. paliurus* plant, such as the bark and roots, to gain a more comprehensive understanding of flavonoid biosynthesis throughout the plant. Finally, given the potential applications of our findings in agriculture and medicine, further studies could explore the feasibility of manipulating the identified gene modules to enhance flavonoid production in other plant species. Our research provides a detailed and comprehensive analysis of the seasonal dynamics of flavonoid biosynthesis in *C. paliurus*. By linking gene expression patterns with flavonoid production, our study not only advances our understanding of plant secondary metabolism but also offers new strategies for enhancing the nutritional and medicinal value of this and other plant species. The innovative methodologies employed, particularly the use of co-expression network analysis, could serve as a model for future studies aimed at unraveling the complex regulatory networks that govern secondary metabolite biosynthesis in plants.

In this study, the Pearson correlation analysis between transcription factor expression and flavonoid content across different months revealed key insights into the role of various transcription factors in the regulation of flavonoid biosynthesis. This analysis underscores the significant seasonal variation in transcription factor activity and its direct impact on flavonoid accumulation, providing a molecular basis for understanding how *C. paliurus* adapts its secondary metabolism to environmental changes. Firstly, members of the *MYB* family, particularly *MYB11, MYB12*, and *MYB111*, exhibited strong positive correlations with flavonols such as quercetin-3-O-glucoside and kaempferol-3-O-glucoside, especially in the late summer and early autumn months. This suggests that these transcription factors play crucial roles in regulating flavonol synthesis during periods of environmental stress, such as increased UV radiation. These findings align with previous research on Arabidopsis thaliana, where these *MYB* transcription factors are known to promote flavonol production under UV light exposure (Stracke et al., 2007). Additionally, *MYB75* (*PAP1*) and *MYB90* (*PAP2*) demonstrated strong correlations with anthocyanin content, particularly cyanidin-3-O-glucoside, suggesting their involvement in anthocyanin biosynthesis during periods of high light intensity, consistent with findings in *Vitis vinifera* (Kobayashi et al., 2004). Furthermore, *bHLH* and *WD40* transcription factors showed strong positive correlations with both flavonols and anthocyanins, indicating their likely involvement in the formation of the *MYB-bHLH-WD40* complex, which regulates the entire flavonoid biosynthesis pathway. This complex has been well-characterized in other species, such as *Petunia hybrida*, where it plays a critical role in anthocyanin biosynthesis (Quattrocchio et al., 2006)). Similarly, *TT8*, a *bHLH* transcription factor, showed a significant positive correlation with proanthocyanidin content during the early autumn period, consistent with its role in *Arabidopsis* seed development, where it regulates proanthocyanidin synthesis (Baudry et al., 2004). Interestingly, *MYB4* exhibited a negative correlation with flavonoid content, particularly in late summer, suggesting that it may act as a repressor of flavonoid biosynthesis by downregulating the expression of chalcone synthase (*CHS*). This aligns with previous studies in *Arabidopsis*, where *MYB4* has been shown to repress *CHS* expression when flavonoid levels are sufficiently high (Jin et al., 2000). Additionally, *MYC2*, a *bHLH* transcription factor involved in stress response, was positively correlated with quercetin glycosides, indicating that it plays a role in enhancing flavonoid biosynthesis as part of the plant defense against environmental stress. Similar regulatory functions have been observed in *Arabidopsis*, where *MYC2* controls jasmonate-induced flavonoid biosynthesis under stress conditions (Dombrecht et al., 2007). *WRKY33*, a *WRKY* transcription factor, showed a strong positive correlation with kaempferol glycosides, especially during periods of environmental stress, indicating its role in upregulating flavonoid biosynthesis as part of the plant defense response. This is consistent with studies in *Oryza sativa*, where *WRKY* has been implicated in stress-induced flavonoid production (Jan et al., 2022). Additionally, members of the *NAC* family of transcription factors were moderately correlated with flavonoid content, suggesting they may coordinate developmental and metabolic processes during seasonal transitions, similar to their role in regulating flavonoid biosynthesis in *Camellia sinensis* (Song et al., 2024). Lastly, *EIN3* (Ethylene-Insensitive 3) and *ABI5* (Abscisic Acid Insensitive 5) demonstrated positive correlations with flavonoid content, particularly with anthocyanins, during late summer and early autumn. This suggests that ethylene and abscisic acid (ABA) signaling pathways are important regulators of flavonoid biosynthesis as the plant transitions towards dormancy. Similar functions for *EIN3* and *ABI5* have been observed in *Cucumic sativus* (Wu et al., 2022) and *Vitis vinifera* (Dong et al., 2020), where these transcription factors regulate flavonoid accumulation in response to environmental stress and developmental cues. These results not only validate previous research but also provide novel insights into the seasonal regulation of flavonoid biosynthesis in *C. paliurus*.

By integrating transcriptomic and metabolomic data, this study presents a comprehensive view of the molecular mechanisms governing flavonoid production in *C. paliurus*, highlighting the critical role of transcription factors in adapting to seasonal changes. These findings provide a valuable foundation for future genetic manipulation and breeding strategies aimed at enhancing flavonoid content in *C. paliurus*, and they extend our understanding of flavonoid regulation beyond model organisms to a commercially and medicinally important species. However, we acknowledge a key limitation in our study, it is that while we identified correlations between the expression of transcription factors and flavonoid levels, we did not perform direct validation of their roles. This gap represents an important area for future investigation. Despite this limitation, our study lays the groundwork for future research that can integrate more robust validation techniques. By exploring additional omics approaches and validating the transcriptional regulation of key genes, future studies will be able to refine our understanding of the molecular networks controlling flavonoid production in *C. paliurus*. This will be crucial for advancing genetic and breeding efforts to optimize flavonoid yields and enhance the medicinal potential of this species.

## 5 Conclusion

This study explored the seasonal dynamics of flavonoid biosynthesis in *C. paliurus*, integrating transcriptomic and metabolomic analyses to uncover the molecular mechanisms driving these variations. Our findings show that flavonoid content peaks in late summer, driven by the upregulation of key biosynthetic genes and transcription factors, particularly *MYB* and *bHLH* families, which regulate flavonoid production in response to environmental stress. Co-expression network analysis identified gene modules linked to specific flavonoids, such as kaempferol derivatives, highlighting potential targets for genetic enhancement of flavonoid content. The Pearson correlation analysis further confirmed the central role of transcription factors like *MYB11, MYB12, MYB111, MYB75*, and *bHLH* in regulating flavonol and anthocyanin biosynthesis. Seasonal changes in these factors underscore their role in the plant’s response to environmental changes, enhancing our understanding of the genetic control mechanisms behind flavonoid production. By integrating transcriptomic and metabolomic data, this study provides a comprehensive view of flavonoid biosynthesis regulation across seasons. These insights not only broaden our understanding of plant secondary metabolism but also suggest new opportunities for optimizing flavonoid production through targeted genetic approaches. This research lays a foundation for future efforts to enhance flavonoid content in *C. paliurus* and other economically and medicinally important plants, with potential applications in functional foods, medicine, agriculture, and biotechnology.

## Data Availability

Raw sequencing data for RNA-seq have been deposited and is available in the BIG Sub system under BioProject accession number CRA005839 (https://ngdc.cncb.ac.cn/gsa).
