# Supplementary material for: Seasonal dynamics and molecular regulation of flavonoid biosynthesis in Cyclocarya paliurus (Batal.) Iljinsk
Source: Front Plant Sci. 2025 Mar 4;16:1525226. doi: 10.3389/fpls.2025.1525226 (PMC11917369; doi:10.3389/fpls.2025.1525226)
Supplement: Supplementary file 1 [file Table1.docx]

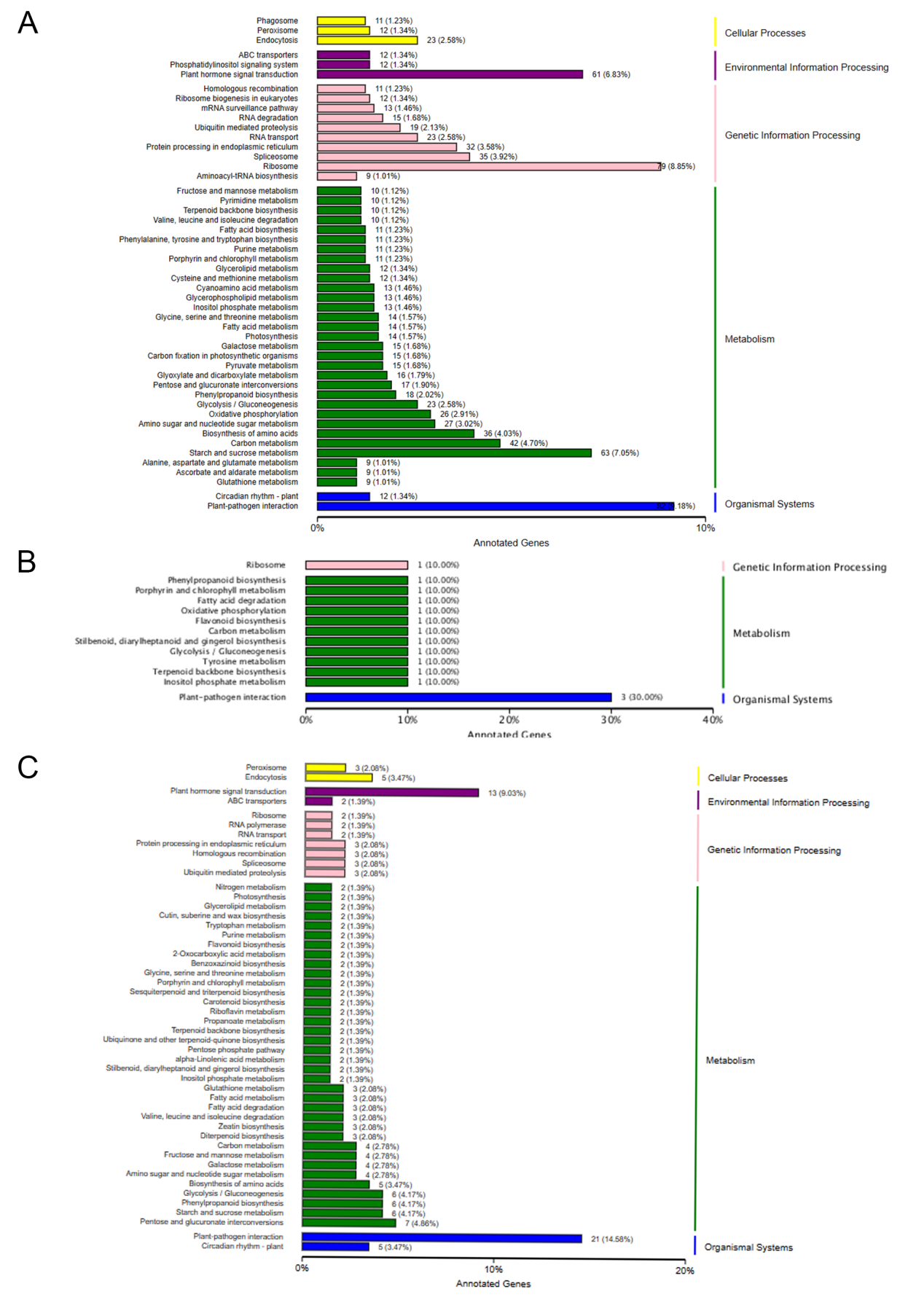


**Figure S1** KEGG enrichment analysis for DEGs in C. paliurus. **A:** KEGG pathway enrichment analysis for DEGs between 8M-ML and 9M-ML samples. **B:** KEGG pathway enrichment analysis for DEGs between 9M-ML and 10M-ML samples. **C:** KEGG pathway enrichment analysis for DEGs between 10M-ML and 11M-ML samples.
