# Supplementary material for: Seasonal dynamics and molecular regulation of flavonoid biosynthesis in Cyclocarya paliurus (Batal.) Iljinsk
Source: Front Plant Sci. 2025 Mar 4;16:1525226. doi: 10.3389/fpls.2025.1525226 (PMC11917369; doi:10.3389/fpls.2025.1525226)
Supplement: Supplementary file 2 [file Table2.docx]

**Supplementary tables**

**Table S1 The data for metabolite compound classification proportion Heatmap**

| Classification of substances | 8M-ML1 | 8M-ML2 | 8M-ML3 | 9M-ML1 | 9M-ML2 | 9M-ML3 | 10M-ML1 | 10M-ML2 | 10M-ML3 | 11M-ML1 | 11M-ML2 | 11M-ML3 |
| --- | --- | --- | --- | --- | --- | --- | --- | --- | --- | --- | --- | --- |
| Amino acids and their derivatives | 1.49E+08 | 1.36E+08 | 1.45E+08 | 2.13E+08 | 2.54E+08 | 2.17E+08 | 1.45E+08 | 1.57E+08 | 1.50E+08 | 8.37E+07 | 9.03E+07 | 7.48E+07 |
| Phenolic acid | 2.43E+08 | 2.37E+08 | 2.63E+08 | 2.24E+08 | 2.27E+08 | 2.29E+08 | 2.68E+08 | 2.70E+08 | 2.72E+08 | 2.78E+08 | 2.93E+08 | 3.01E+08 |
| Nucleotides and their derivatives | 5.71E+07 | 5.55E+07 | 4.51E+07 | 1.25E+08 | 1.33E+08 | 1.14E+08 | 8.07E+07 | 9.21E+07 | 9.39E+07 | 4.62E+07 | 5.06E+07 | 6.30E+07 |
| flavone | 3.27E+08 | 3.62E+08 | 3.62E+08 | 1.95E+08 | 2.12E+08 | 1.90E+08 | 4.05E+08 | 4.54E+08 | 4.09E+08 | 4.23E+08 | 4.54E+08 | 3.50E+08 |
| Lignans and coumarins | 4.03E+07 | 4.24E+07 | 4.17E+07 | 3.05E+07 | 3.61E+07 | 3.43E+07 | 5.16E+07 | 5.33E+07 | 5.35E+07 | 6.19E+07 | 5.31E+07 | 5.49E+07 |
| Other categories | 9.62E+07 | 9.79E+07 | 1.05E+08 | 8.98E+07 | 8.74E+07 | 9.49E+07 | 1.34E+08 | 1.33E+08 | 1.25E+08 | 9.68E+07 | 9.92E+07 | 8.86E+07 |
| tannin | 7.20E+06 | 1.03E+07 | 8.69E+06 | 1.82E+06 | 1.87E+06 | 1.56E+06 | 7.16E+06 | 6.64E+06 | 1.38E+07 | 7.84E+06 | 1.16E+07 | 1.08E+07 |
| alkaloid | 4.13E+07 | 4.09E+07 | 4.83E+07 | 4.99E+07 | 5.51E+07 | 4.82E+07 | 3.68E+07 | 3.63E+07 | 3.72E+07 | 2.38E+07 | 3.27E+07 | 2.77E+07 |
| terpene | 6.23E+07 | 1.02E+08 | 7.69E+07 | 4.37E+07 | 4.93E+07 | 5.15E+07 | 3.34E+07 | 2.96E+07 | 3.68E+07 | 7.99E+07 | 8.53E+07 | 1.13E+08 |
| Organic acid | 1.67E+08 | 1.46E+08 | 1.48E+08 | 1.93E+08 | 2.24E+08 | 1.99E+08 | 2.30E+08 | 2.46E+08 | 2.59E+08 | 2.11E+08 | 2.11E+08 | 1.49E+08 |
| lipin | 7.32E+07 | 7.60E+07 | 7.07E+07 | 1.10E+08 | 1.16E+08 | 1.16E+08 | 7.81E+07 | 7.54E+07 | 7.44E+07 | 7.68E+07 | 7.56E+07 | 8.15E+07 |
| Sum total | 1.26E+09 | 1.31E+09 | 1.31E+09 | 1.28E+09 | 1.40E+09 | 1.30E+09 | 1.47E+09 | 1.55E+09 | 1.52E+09 | 1.39E+09 | 1.46E+09 | 1.31E+09 |
| The proportion of flavonoids | 26% | 28% | 28% | 15% | 15% | 15% | 28% | 29% | 27% | 30% | 31% | 27% |

**Table S2 The flavonoid compounds identified in *C. paliurus* leaves**

| **Compounds** | **Molecular Weight (Da)** | **Formula** | **Ionization model** | **Class I** | **Class II** | **CAS** |
| --- | --- | --- | --- | --- | --- | --- |
| Cyanidin-3-O-galactoside* | 4.49E+02 | C21H21O11+ | [M]+ | Flavonoids | Anthocyanins | 142506-26-1 |
| Tricin (5,7,4'-Trihydroxy-3',5'-dimethoxyflavone) | 3.30E+02 | C17H14O7 | [M+H]+ | Flavonoids | Flavonoid | 520-32-1 |
| Cyanidin-3-O-glucoside (Kuromanin)* | 4.49E+02 | C21H21O11+ | [M]+ | Flavonoids | Anthocyanins | 47705-70-4 |
| Quercetin-3-O-neohesperidoside | 6.10E+02 | C27H30O16 | [M+H]+ | Flavonoids | Flavonols | 117611-67-3 |
| Kaempferol-3-O-(3''-O-p-Coumaroyl)rhamnoside | 5.78E+02 | C30H26O12 | [M+H]+ | Flavonoids | Flavonoid | - |
| Kaempferol-3-O-(4''-O-p-Coumaroyl)rhamnoside | 5.78E+02 | C30H26O12 | [M+H]+ | Flavonoids | Flavonoid | - |
| Luteolin-7-O-glucuronide | 4.62E+02 | C21H18O12 | [M+H]+ | Flavonoids | Flavonoid | 29741-10-4 |
| Herbacetin-3-O-glucuronide | 4.78E+02 | C21H18O13 | [M+H]+ | Flavonoids | Flavonols | - |
| Luteolin-4'-O-glucoside | 4.48E+02 | C21H20O11 | [M+H]+ | Flavonoids | Flavonoid | 6920-38-3 |
| Kaempferol-3-O-glucoside (Astragalin) | 4.48E+02 | C21H20O11 | [M+H]+ | Flavonoids | Flavonols | 480-10-4 |
| Quercetin-4'-O-glucoside (Spiraeoside) | 4.64E+02 | C21H20O12 | [M-H]- | Flavonoids | Flavonols | 20229-56-5 |
| Quercetin-7-O-glucoside* | 4.64E+02 | C21H20O12 | [M-H]- | Flavonoids | Flavonols | 491-50-9 |
| Catechin | 2.90E+02 | C15H14O6 | [M-H]- | Flavonoids | Flavanols | 154-23-4 |
| 5,7,4'-Trihydroxy-6,8-dimethoxyisoflavone-7-O-galactoside | 4.92E+02 | C23H24O12 | [M+H]+ | Flavonoids | Isoflavones | - |
| Catechin-(7,8-bc)-4α-(3,4-dihydroxyphenyl)-dihydro-2-(3H)-one | 4.52E+02 | C24H20O9 | [M-H]- | Flavonoids | Flavanols | - |
| Quercetin-3-O-xyloside (Reynoutrin)* | 4.34E+02 | C20H18O11 | [M+H]+ | Flavonoids | Flavonols | 549-32-6 |
| Epicatechin | 2.90E+02 | C15H14O6 | [M+H]+ | Flavonoids | Flavanols | 490-46-0 |
| Avicularin(Quercetin-3-O-α-L-arabinofuranoside) | 4.34E+02 | C20H18O11 | [M+H]+ | Flavonoids | Flavonols | 572-30-5 |
| Quercetin-3-O-robinobioside | 6.10E+02 | C27H30O16 | [M-H]- | Flavonoids | Flavonols | 52525-35-6 |
| Ayanin (3',5-Dihydroxy-3,4',7-Trimethoxyflavone) | 3.44E+02 | C18H16O7 | [M+H]+ | Flavonoids | Flavonoid | 572-32-7 |
| Chrysoeriol-5-O-glucoside | 4.62E+02 | C22H22O11 | [M-H]- | Flavonoids | Flavonoid | - |
| Quercetin-3-O-galactoside (Hyperin) | 4.64E+02 | C21H20O12 | [M-H]- | Flavonoids | Flavonols | 482-36-0 |
| Quercetin-3-O-rhamnoside(Quercitrin) | 4.48E+02 | C21H20O11 | [M+H]+ | Flavonoids | Flavonols | 522-12-3 |
| Luteolin-7-O-glucoside (Cynaroside) | 4.48E+02 | C21H20O11 | [M+H]+ | Flavonoids | Flavonoid | 5373-11-5 |
| Carthamone | 4.48E+02 | C21H20O11 | [M-H]- | Flavonoids | Chalcones | 86579-00-2 |
| Kaempferol-3-O-galactoside (Trifolin) | 4.48E+02 | C21H20O11 | [M-H]- | Flavonoids | Flavonols | 23627-87-4 |
| Quercetin-3-O-glucoside (Isoquercitrin)* | 4.64E+02 | C21H20O12 | [M-H]- | Flavonoids | Flavonols | 482-35-9 |
| Quercetin-3-O-rutinoside (Rutin) | 6.10E+02 | C27H30O16 | [M-H]- | Flavonoids | Flavonols | 153-18-4 |
| Hispidulin (5,7,4'-Trihydroxy-6-methoxyflavone) | 3.00E+02 | C16H12O6 | [M+H]+ | Flavonoids | Flavonoid | 1447-88-7 |
| Naringenin-7-O-glucoside (Prunin) | 4.34E+02 | C21H22O10 | [M-H]- | Flavonoids | Dihydroflavone | 529-55-5 |
| Myricetin-3-O-glucuronide | 4.94E+02 | C21H18O14 | [M+H]+ | Flavonoids | Flavonols | 77363-65-6 |
| Apigenin-6-C-glucoside (Isovitexin) | 4.32E+02 | C21H20O10 | [M-H]- | Flavonoids | Flavonoid carbonoside | 29702-25-8 |
| 7-O-Methyleriodictyol | 3.02E+02 | C16H14O6 | [M+H]+ | Flavonoids | Flavanols | 51857-11-5 |
| Kaempferol-3-O-glucuronide | 4.62E+02 | C21H18O12 | [M-H]- | Flavonoids | Flavonols | 22688-78-4 |
| Diosmetin (5,7,3'-Trihydroxy-4'-methoxyflavone) | 3.00E+02 | C16H12O6 | [M-H]- | Flavonoids | Flavonoid | 520-34-3 |
| Kaempferol-3-O-neohesperidoside* | 5.94E+02 | C27H30O15 | [M+H]+ | Flavonoids | Flavonols | 32602-81-6 |
| Kaempferol-3-O-(6''-malonyl)galactoside* | 5.34E+02 | C24H22O14 | [M+H]+ | Flavonoids | Flavonols | - |
| Isorhamnetin-7-O-glucoside (Brassicin) | 4.78E+02 | C22H22O12 | [M+H]+ | Flavonoids | Flavonols | 6743-96-0 |
| Tricin-7-O-Glucoside | 4.92E+02 | C23H24O12 | [M+H]+ | Flavonoids | Flavonoid | 32769-01-0 |
| Quercetin-4′-O-glucuronide | 4.78E+02 | C21H18O13 | [M-H]- | Flavonoids | Flavonols | 201463-36-7 |
| Tectoridin | 4.62E+02 | C22H22O11 | [M+H]+ | Flavonoids | Isoflavones | 611-40-5 |
| Taxifolin-3'-O-glucoside | 4.66E+02 | C21H22O12 | [M-H]- | Flavonoids | Flavonoid | 31106-05-5 |
| Kaempferol-4'-O-glucoside | 4.48E+02 | C21H20O11 | [M+H]+ | Flavonoids | Flavonoid | - |
| Butin | 2.72E+02 | C15H12O5 | [M+H]+ | Flavonoids | Dihydroflavone | 492-14-8 |
| Kaempferol-3-O-(4''-O-acetyl)rhamnoside | 4.74E+02 | C23H22O11 | [M+H]+ | Flavonoids | Flavonoid | - |
| 7,8-Dihydroxy-5,6,4'-trimethoxyflavone | 3.44E+02 | C18H16O7 | [M+H]+ | Flavonoids | Flavonoid | - |
| Quercetin-3-O-arabinoside (Guaijaverin)* | 4.34E+02 | C20H18O11 | [M-H]- | Flavonoids | Flavonols | 22255-13-6 |
| 5,2'-Dihydroxy-7,8-dimethoxyflavone glycosides | 4.76E+02 | C23H24O11 | [M+H]+ | Flavonoids | Flavonoid | - |
| Quercetin-3-O-sambubioside | 5.96E+02 | C26H28O16 | [M+H]+ | Flavonoids | Flavonols | 83048-35-5 |
| Quercetin-3-O-sophoroside (Baimaside) | 6.26E+02 | C27H30O17 | [M+H]+ | Flavonoids | Flavonols | 18609-17-1 |
| Cyanidin-3-O-arabinoside | 4.19E+02 | C20H19O10+ | [M]+ | Flavonoids | Anthocyanins | 27214-72-8 |
| Naringenin (5,7,4'-Trihydroxyflavanone)* | 2.72E+02 | C15H12O5 | [M-H]- | Flavonoids | Dihydroflavone | 480-41-1 |
| Syringetin-7-O-glucoside | 5.08E+02 | C23H24O13 | [M+H]+ | Flavonoids | Flavonoid | - |
| Limocitrin-3-O-glucoside | 5.08E+02 | C23H24O13 | [M+H]+ | Flavonoids | Flavonoid | - |
| 5,7-Dihydroxy-3',4',5'-trimethoxyflavone | 3.44E+02 | C18H16O7 | [M+H]+ | Flavonoids | Flavonoid | 18103-42-9 |
| Limocitrin-3-O-galactoside | 5.08E+02 | C23H24O13 | [M+H]+ | Flavonoids | Flavonoid | - |
| Quercetin-3-O-(2''-O-glucosyl)glucuronide | 6.40E+02 | C27H28O18 | [M+H]+ | Flavonoids | Flavonols | - |
| Iristectorigenin B | 3.30E+02 | C17H14O7 | [M+H]+ | Flavonoids | Isoflavones | 39012-01-6 |
| Catechin-(7,8-bc)-4β-(3,4-dihydroxyphenyl)-dihydro-2-(3H)-one | 4.52E+02 | C24H20O9 | [M-H]- | Flavonoids | Flavanols | - |
| Taxifolin-3-O-rhamnoside (Astilbin) | 4.50E+02 | C21H22O11 | [M-H]- | Flavonoids | Dihydroflavonol | 29838-67-3 |
| Quercetin | 3.02E+02 | C15H10O7 | [M+H]+ | Flavonoids | Flavonols | 117-39-5 |
| Eriodictyol (5,7,3',4'-Tetrahydroxyflavanone) | 2.88E+02 | C15H12O6 | [M-H]- | Flavonoids | Dihydroflavone | 552-58-9 |
| Tricetin (5,7,3',4',5'-Pentahydroxyflavone) | 3.02E+02 | C15H10O7 | [M-H]- | Flavonoids | Flavonoid | 520-31-0 |
| Quercetin-7-O-(6''-malonyl)glucoside | 5.50E+02 | C24H22O15 | [M+H]+ | Flavonoids | Flavonols | - |
| 7-O-Methylnaringenin | 2.86E+02 | C16H14O5 | [M+H]+ | Flavonoids | Flavonoid | - |
| Epigallocatechin | 3.06E+02 | C15H14O7 | [M-H]- | Flavonoids | Flavanols | 970-74-1 |
| Quercetin-3-O-(6''-malonyl)galactoside | 5.50E+02 | C24H22O15 | [M-H]- | Flavonoids | Flavonols | - |
| Myricetin-3-O-glucoside | 4.80E+02 | C21H20O13 | [M-H]- | Flavonoids | Flavonols | 19833-12-6 |
| Aromadendrin (Dihydrokaempferol) | 2.88E+02 | C15H12O6 | [M-H]- | Flavonoids | Dihydroflavonol | 480-20-6 |
| Quercetin-3-O-(2''-O-galactosyl)glucoside | 6.26E+02 | C27H30O17 | [M+H]+ | Flavonoids | Flavonols | 56782-99-1 |
| Kaempferol (3,5,7,4'-Tetrahydroxyflavone) | 2.86E+02 | C15H10O6 | [M-H]- | Flavonoids | Flavonols | 520-18-3 |
| Robinetin | 3.02E+02 | C15H10O7 | [M+H]+ | Flavonoids | Flavonoid | 490-31-3 |
| Epicatechin glucoside | 4.52E+02 | C21H24O11 | [M-H]- | Flavonoids | Flavanols | - |
| Myricetin-3-O-rhamnoside (Myricitrin) | 4.64E+02 | C21H20O12 | [M-H]- | Flavonoids | Flavonols | 17912-87-7 |
| Fisetin | 2.86E+02 | C15H10O6 | [M+H]+ | Flavonoids | Flavonols | 528-48-3 |
| Apigenin-5-O-glucoside | 4.32E+02 | C21H20O10 | [M+H]+ | Flavonoids | Flavonoid | 28757-27-9 |
| 5,4′-Dihydroxy-3,6,7,3′-tetramethoxyflavone | 3.74E+02 | C19H18O8 | [M+H]+ | Flavonoids | Flavonoid | 130252-52-7 |
| Diosmetin-7-O-galactoside | 4.62E+02 | C22H22O11 | [M+H]+ | Flavonoids | Flavonoid | - |
| Kaempferol-3-O-arabinoside | 4.18E+02 | C20H18O10 | [M+H]+ | Flavonoids | Flavonoid | - |
| Laricitrin-3-O-glucoside | 4.94E+02 | C22H22O13 | [M+H]+ | Flavonoids | Flavonoid | 39986-90-8 |
| Kaempferol-3-O-(2''-p-Coumaroyl)glucoside | 5.94E+02 | C30H26O13 | [M+H]+ | Flavonoids | Flavonoid | 137018-32-7 |
| Luteolin (5,7,3',4'-Tetrahydroxyflavone) | 2.86E+02 | C15H10O6 | [M-H]- | Flavonoids | Flavonoid | 491-70-3 |
| Kaempferol-3-O-rhamnoside (Afzelin)(Kaempferin) | 4.32E+02 | C21H20O10 | [M-H]- | Flavonoids | Flavonols | 482-39-3 |
| Quercetin-3-O-(6''-acetyl)galactoside | 5.06E+02 | C23H22O13 | [M-H]- | Flavonoids | Flavonols | - |
| Kaempferol-3-O-rutinoside(Nicotiflorin)* | 5.94E+02 | C27H30O15 | [M-H]- | Flavonoids | Flavonoid | 17650-84-9 |
| 3-Hydroxy-4',5,7-Trimethoxyflavanone | 3.30E+02 | C18H18O6 | [M+H]+ | Flavonoids | Flavonoid | 76792-94-4 |
| Phloretin-2'-O-glucoside (Phlorizin) | 4.36E+02 | C21H24O10 | [M-H]- | Flavonoids | Chalcones | 60-81-1 |
| Naringenin-7-O-Rutinoside(Narirutin)* | 5.80E+02 | C27H32O14 | [M-H]- | Flavonoids | Dihydroflavone | 14259-46-2 |
| Apigenin | 2.70E+02 | C15H10O5 | [M+H]+ | Flavonoids | Flavonoid | 520-36-5 |
| Kaempferol-3-O-(6''-malonyl)glucoside* | 5.34E+02 | C24H22O14 | [M+H]+ | Flavonoids | Flavonols | - |
| 6,7,8-Tetrahydroxy-5-methoxyflavone | 3.00E+02 | C16H12O6 | [M+H]+ | Flavonoids | Flavonoid | - |
| Naringenin-7-O-Neohesperidoside(Naringin)* | 5.80E+02 | C27H32O14 | [M-H]- | Flavonoids | Dihydroflavone | 10236-47-2 |
| Kaempferol-3-O-glucuronide-7-O-glucoside | 6.24E+02 | C27H28O17 | [M-H]- | Flavonoids | Flavonols | - |
| Luteolin-7-O-gentiobioside | 6.10E+02 | C27H30O16 | [M+H]+ | Flavonoids | Flavonoid | - |
| Phloretin | 2.74E+02 | C15H14O5 | [M-H]- | Flavonoids | Chalcones | 60-82-2 |
| Isorhamnetin-3-O-arabinoside | 4.48E+02 | C21H20O11 | [M+H]+ | Flavonoids | Flavonols | - |
| Luteolin-7-O-(6''-caffeoyl)rhamnoside | 5.94E+02 | C30H26O13 | [M-H]- | Flavonoids | Flavonoid | - |
| Quercetin-3-O-(6''-p-Coumaroyl)glucoside | 6.10E+02 | C30H26O14 | [M+H]+ | Flavonoids | Flavonols | - |
| Quercetin-3-O-(6''-p-Coumaroyl)galactoside | 6.10E+02 | C30H26O14 | [M+H]+ | Flavonoids | Flavonols | - |
| Syringetin | 3.46E+02 | C17H14O8 | [M-H]- | Flavonoids | Flavonoid | 4423-37-4 |
| Sieboldin | 4.52E+02 | C21H24O11 | [M-H]- | Flavonoids | Chalcones | 18777-73-6 |
| Kaempferol-3-O-(2''-p-Coumaroyl)galactoside | 5.94E+02 | C30H26O13 | [M+H]+ | Flavonoids | Flavonoid | - |
| Dihydroquercetin(Taxifolin) | 3.04E+02 | C15H12O7 | [M-H]- | Flavonoids | Dihydroflavonol | 480-18-2 |
| Quercetin-5,4ʹ-di-O-glucoside | 6.26E+02 | C27H30O17 | [M+H]+ | Flavonoids | Flavonoid | - |
| Kaempferol-3-O-(6''-p-Coumaroyl)galactoside | 5.94E+02 | C30H26O13 | [M+H]+ | Flavonoids | Flavonoid | - |
| Aureusidin | 2.86E+02 | C15H10O6 | [M+H]+ | Flavonoids | Sinensetin | 38216-54-5 |
| Kaempferol-3-O-(6''-p-Coumaroyl)glucoside (Tiliroside) | 5.94E+02 | C30H26O13 | [M-H]- | Flavonoids | Flavonols | 20316-62-5 |
| Gallocatechin | 3.06E+02 | C15H14O7 | [M+H]+ | Flavonoids | Flavanols | 970-73-0 |
| Quercetin-3-O-(2''-acetyl)rhamnoside | 4.90E+02 | C23H22O12 | [M+H]+ | Flavonoids | Flavonols | - |
| Dihydrocharcone-4'-O-glucoside | 4.36E+02 | C21H24O10 | [M-H]- | Flavonoids | Chalcones | - |
| Kaempferol-3-O-(6''-acetyl)glucoside | 4.90E+02 | C23H22O12 | [M-H]- | Flavonoids | Flavonols | - |
| Phloretin-4'-O-glucoside (Trilobatin) | 4.36E+02 | C21H24O10 | [M-H]- | Flavonoids | Chalcones | 4192-90-9 |
| 3,7-Di-O-methylquercetin | 3.30E+02 | C17H14O7 | [M-H]- | Flavonoids | Flavonols | 2068-02-2 |
| 3-O-Methylquercetin | 3.16E+02 | C16H12O7 | [M-H]- | Flavonoids | Flavonols | 1486-70-0 |
| Kaempferol-3-O-arabinoside (Juglanin) | 4.18E+02 | C20H18O10 | [M-H]- | Flavonoids | Flavonols | 5041-67-8 |
| Isorhamnetin-3,7-O-diglucoside | 6.40E+02 | C28H32O17 | [M+H]+ | Flavonoids | Flavonols | 6758-51-6 |
| Kaempferol-3,7-di-O-glucoside | 6.10E+02 | C27H30O16 | [M+H]+ | Flavonoids | Flavonoid | - |
| Tricin-7-O-(2''-Sinapoyl)glucoside | 6.98E+02 | C34H34O16 | [M+H]+ | Flavonoids | Flavonoid | - |
| Acacetin-7-O-galactoside* | 4.46E+02 | C22H22O10 | [M+H]+ | Flavonoids | Flavonoid | - |
| Luteolin-7-O-rutinoside* | 5.94E+02 | C27H30O15 | [M+H]+ | Flavonoids | Flavonoid | 3563-98-2 |
| Acacetin-7-O-glucoside (Tilianin)* | 4.46E+02 | C22H22O10 | [M+H]+ | Flavonoids | Flavonoid | 4291-60-5 |
| Catechin-catechin-catechin | 8.66E+02 | C45H38O18 | [M-H]- | Flavonoids | Flavanols | - |
| Kaempferol-3,7-O-dirhamnoside (Kaempferitrin) | 5.78E+02 | C27H30O14 | [M+H]+ | Flavonoids | Flavonols | 482-38-2 |
| Luteolin-7-O-(2''-O-rhamnosyl)rutinoside | 7.40E+02 | C33H40O19 | [M+H]+ | Flavonoids | Flavonoid | - |
| Myricetin-3-O-xyloside | 4.50E+02 | C20H18O12 | [M-H]- | Flavonoids | Flavonols | - |
| Luteolin-7-O-neohesperidoside (Lonicerin)* | 5.94E+02 | C27H30O15 | [M+H]+ | Flavonoids | Flavonoid | 25694-72-8 |
| Kaempferol-3-O-sambubioside | 5.80E+02 | C26H28O15 | [M+H]+ | Flavonoids | Flavonoid | 27661-51-4 |
| 4',5-Dihydroxy-3',5'-dimethoxyflavone | 3.14E+02 | C17H14O6 | [M+H]+ | Flavonoids | Flavonoid | - |
| 2'-Hydroxy-3,4,5,4',6'-pentamethoxychalcone | 3.74E+02 | C20H22O7 | [M+H]+ | Flavonoids | Chalcones | - |
| Dihydromyricetin (Ampelopsin) | 3.20E+02 | C15H12O8 | [M-H]- | Flavonoids | Dihydroflavonol | 27200-12-0 |
| Luteolin-7-O-(6''-sinapoyl)glucoside | 6.54E+02 | C32H30O15 | [M+H]+ | Flavonoids | Flavonoid | - |
| Myricetin-3-O-arabinoside | 4.50E+02 | C20H18O12 | [M-H]- | Flavonoids | Flavonols | 132679-85-7 |
| Limocitrin (5,7,4'-trihydroxy-8,3'-dimethoxyflavone) | 3.46E+02 | C17H14O8 | [M+H]+ | Flavonoids | Flavonoid | 489-33-8 |
| 5,8,4'-Trihydroxy-6,7-dimethoxyflavone | 3.30E+02 | C17H14O7 | [M+H]+ | Flavonoids | Flavonoid | 98755-25-0 |
| Eriodictyol-7-O-glucoside | 4.50E+02 | C21H22O11 | [M+H]+ | Flavonoids | Dihydroflavone | 38965-51-4 |
| Pinobanksin* | 2.72E+02 | C15H12O5 | [M-H]- | Flavonoids | Dihydroflavonol | 548-82-3 |
| Naringenin-7-O-(6''-malonyl)glucoside | 5.20E+02 | C24H24O13 | [M+H]+ | Flavonoids | Flavanols | - |
| 3’,4’-Dihydroxy-7,5’-dimethoxyflavone | 3.14E+02 | C17H14O6 | [M+H]+ | Flavonoids | Flavonoid | - |
| Isorhamnetin | 3.16E+02 | C16H12O7 | [M-H]- | Flavonoids | Flavonols | 480-19-3 |
| Luteolin-7-O-(6''-malonyl)glucoside | 5.34E+02 | C24H22O14 | [M+H]+ | Flavonoids | Flavonoid | - |
| Isosakuranetin (5,7-Dihydroxy-4'-methoxyflavanone) | 2.86E+02 | C16H14O5 | [M+H]+ | Flavonoids | Dihydroflavone | 480-43-3 |
| Hypolaetin | 3.02E+02 | C15H10O7 | [M-H]- | Flavonoids | Flavonoid | 27696-41-9 |
| 5,7,3',4',5'-Pentahydroxydihydroflavone | 3.04E+02 | C15H12O7 | [M-H]- | Flavonoids | Dihydroflavone | - |
| Myricetin-3-O-(6''-malony)glucoside | 5.66E+02 | C24H22O16 | [M+H]+ | Flavonoids | Flavonols | - |

**Table S3 The differental flavonoid metabolites of 8M-ML_vs_9M-ML**

| Compounds | Class II | 8M-ML1 | 8M-ML2 | 8M-ML3 | 9M-ML1 | 9M-ML2 | 9M-ML3 | Fold_Change | Log2FC | Type |
| --- | --- | --- | --- | --- | --- | --- | --- | --- | --- | --- |
| Dihydromyricetin (Ampelopsin) | Dihydroflavonol | 9.00E+00 | 9.00E+00 | 9.00E+00 | 3.77E+04 | 5.00E+04 | 3.30E+04 | 4.47E+03 | 1.21E+01 | up |
| Isosakuranetin (5,7-Dihydroxy-4'-methoxyflavanone) | Dihydroflavone | 9.00E+00 | 9.00E+00 | 9.00E+00 | 1.32E+04 | 8.30E+03 | 1.17E+04 | 1.23E+03 | 1.03E+01 | up |
| 5,2'-Dihydroxy-7,8-dimethoxyflavone glycosides | Flavonoid | 1.76E+05 | 1.59E+05 | 6.00E+04 | 2.06E+06 | 1.66E+06 | 2.38E+06 | 1.55E+01 | 3.95E+00 | up |
| 7-O-Methyleriodictyol | Flavanols | 1.41E+05 | 7.46E+04 | 1.37E+05 | 2.21E+06 | 1.35E+06 | 1.78E+06 | 1.51E+01 | 3.92E+00 | up |
| Ayanin (3',5-Dihydroxy-3,4',7-Trimethoxyflavone) | Flavonoid | 1.04E+06 | 3.97E+05 | 8.32E+05 | 8.15E+06 | 7.46E+06 | 7.99E+06 | 1.04E+01 | 3.38E+00 | up |
| Acacetin-7-O-galactoside* | Flavonoid | 6.86E+03 | 6.83E+03 | 8.16E+03 | 6.56E+04 | 5.71E+04 | 6.37E+04 | 8.53E+00 | 3.09E+00 | up |
| Acacetin-7-O-glucoside (Tilianin)* | Flavonoid | 8.66E+03 | 1.38E+04 | 7.51E+03 | 5.07E+04 | 5.18E+04 | 6.08E+04 | 5.45E+00 | 2.45E+00 | up |
| Quercetin-3-O-sambubioside | Flavonols | 8.63E+04 | 9.99E+04 | 6.25E+04 | 3.21E+05 | 4.23E+05 | 3.33E+05 | 4.33E+00 | 2.11E+00 | up |
| Syringetin-7-O-glucoside | Flavonoid | 1.40E+05 | 9.40E+04 | 1.04E+05 | 4.86E+05 | 4.37E+05 | 4.86E+05 | 4.17E+00 | 2.06E+00 | up |
| Limocitrin-3-O-glucoside | Flavonoid | 1.28E+05 | 1.24E+05 | 8.44E+04 | 4.83E+05 | 4.93E+05 | 3.99E+05 | 4.09E+00 | 2.03E+00 | up |
| Apigenin-5-O-glucoside | Flavonoid | 4.56E+04 | 5.88E+04 | 3.09E+04 | 1.62E+05 | 1.31E+05 | 1.68E+05 | 3.41E+00 | 1.77E+00 | up |
| 5,4′-Dihydroxy-3,6,7,3′-tetramethoxyflavone | Flavonoid | 4.45E+05 | 2.50E+05 | 4.01E+05 | 1.15E+06 | 1.19E+06 | 1.11E+06 | 3.15E+00 | 1.65E+00 | up |
| Apigenin-6-C-glucoside (Isovitexin) | Flavonoid carbonoside | 1.34E+06 | 1.23E+06 | 8.52E+05 | 3.89E+06 | 3.67E+06 | 3.11E+06 | 3.12E+00 | 1.64E+00 | up |
| 7,8-Dihydroxy-5,6,4'-trimethoxyflavone | Flavonoid | 1.00E+05 | 8.49E+04 | 6.75E+04 | 2.38E+05 | 2.74E+05 | 2.50E+05 | 3.02E+00 | 1.59E+00 | up |
| Robinetin | Flavonoid | 1.49E+05 | 8.63E+04 | 9.76E+04 | 2.84E+05 | 2.55E+05 | 3.35E+05 | 2.62E+00 | 1.39E+00 | up |
| Kaempferol-3-O-neohesperidoside* | Flavonols | 7.41E+05 | 9.58E+05 | 6.83E+05 | 1.91E+06 | 2.26E+06 | 2.00E+06 | 2.59E+00 | 1.37E+00 | up |
| Quercetin-3-O-neohesperidoside | Flavonols | 2.90E+06 | 2.96E+06 | 2.44E+06 | 6.85E+06 | 8.47E+06 | 5.75E+06 | 2.54E+00 | 1.34E+00 | up |
| Kaempferol-3-O-glucuronide-7-O-glucoside | Flavonols | 5.29E+04 | 3.71E+04 | 4.66E+04 | 8.50E+04 | 1.36E+05 | 1.10E+05 | 2.43E+00 | 1.28E+00 | up |
| Quercetin-3-O-robinobioside | Flavonols | 7.95E+05 | 8.45E+05 | 6.37E+05 | 1.66E+06 | 2.59E+06 | 1.19E+06 | 2.39E+00 | 1.26E+00 | up |
| Quercetin-3-O-rutinoside (Rutin) | Flavonols | 6.40E+05 | 6.35E+05 | 5.21E+05 | 1.12E+06 | 1.83E+06 | 1.19E+06 | 2.30E+00 | 1.20E+00 | up |
| 3’,4’-Dihydroxy-7,5’-dimethoxyflavone | Flavonoid | 5.04E+03 | 4.53E+03 | 3.20E+03 | 8.53E+03 | 8.14E+03 | 1.24E+04 | 2.28E+00 | 1.19E+00 | up |
| 7-O-Methylnaringenin | Flavonoid | 1.06E+05 | 1.11E+05 | 9.59E+04 | 2.55E+05 | 2.00E+05 | 2.33E+05 | 2.20E+00 | 1.14E+00 | up |
| Kaempferol-3-O-rutinoside(Nicotiflorin)* | Flavonoid | 3.17E+04 | 5.40E+04 | 2.50E+04 | 7.11E+04 | 9.00E+04 | 7.93E+04 | 2.17E+00 | 1.12E+00 | up |
| Butin | Dihydroflavone | 1.43E+06 | 1.24E+06 | 1.49E+06 | 2.66E+06 | 2.80E+06 | 3.53E+06 | 2.16E+00 | 1.11E+00 | up |
| 5,7-Dihydroxy-3',4',5'-trimethoxyflavone | Flavonoid | 6.68E+05 | 7.52E+05 | 5.28E+05 | 1.36E+06 | 1.54E+06 | 1.30E+06 | 2.16E+00 | 1.11E+00 | up |
| 3-O-Methylquercetin | Flavonols | 6.41E+04 | 3.12E+04 | 3.57E+04 | 9.31E+04 | 1.03E+05 | 8.51E+04 | 2.15E+00 | 1.10E+00 | up |
| Eriodictyol (5,7,3',4'-Tetrahydroxyflavanone) | Dihydroflavone | 4.46E+05 | 3.23E+05 | 4.53E+05 | 7.93E+05 | 8.05E+05 | 9.74E+05 | 2.10E+00 | 1.07E+00 | up |
| Laricitrin-3-O-glucoside | Flavonoid | 3.84E+05 | 2.76E+05 | 4.38E+05 | 8.13E+05 | 7.84E+05 | 6.64E+05 | 2.06E+00 | 1.04E+00 | up |
| Luteolin (5,7,3',4'-Tetrahydroxyflavone) | Flavonoid | 1.91E+05 | 2.14E+05 | 1.35E+05 | 8.53E+04 | 8.93E+04 | 8.70E+04 | 4.85E-01 | -1.05E+00 | down |
| Kaempferol-3,7-O-dirhamnoside (Kaempferitrin) | Flavonols | 3.88E+04 | 5.10E+04 | 4.31E+04 | 1.64E+04 | 2.97E+04 | 1.53E+04 | 4.62E-01 | -1.11E+00 | down |
| Luteolin-7-O-(6''-caffeoyl)rhamnoside | Flavonoid | 1.14E+05 | 1.35E+05 | 1.20E+05 | 5.86E+04 | 5.58E+04 | 5.43E+04 | 4.58E-01 | -1.13E+00 | down |
| Diosmetin-7-O-galactoside | Flavonoid | 3.95E+05 | 3.77E+05 | 3.46E+05 | 1.58E+05 | 1.64E+05 | 1.89E+05 | 4.57E-01 | -1.13E+00 | down |
| Tricin (5,7,4'-Trihydroxy-3',5'-dimethoxyflavone) | Flavonoid | 2.17E+07 | 2.33E+07 | 1.69E+07 | 8.90E+06 | 1.09E+07 | 7.88E+06 | 4.48E-01 | -1.16E+00 | down |
| 3-Hydroxy-4',5,7-Trimethoxyflavanone | Flavonoid | 1.38E+05 | 1.47E+05 | 1.20E+05 | 5.22E+04 | 7.29E+04 | 4.96E+04 | 4.32E-01 | -1.21E+00 | down |
| 5,7,4'-Trihydroxy-6,8-dimethoxyisoflavone-7-O-galactoside | Isoflavones | 1.07E+07 | 1.28E+07 | 9.56E+06 | 4.26E+06 | 5.87E+06 | 3.40E+06 | 4.09E-01 | -1.29E+00 | down |
| Naringenin-7-O-glucoside (Prunin) | Dihydroflavone | 1.87E+06 | 2.19E+06 | 2.01E+06 | 6.79E+05 | 9.04E+05 | 7.53E+05 | 3.85E-01 | -1.38E+00 | down |
| Luteolin-7-O-(6''-sinapoyl)glucoside | Flavonoid | 3.55E+04 | 3.39E+04 | 2.62E+04 | 1.01E+04 | 7.45E+03 | 1.82E+04 | 3.74E-01 | -1.42E+00 | down |
| Quercetin-3-O-(6''-p-Coumaroyl)galactoside | Flavonols | 1.45E+05 | 1.46E+05 | 1.70E+05 | 4.18E+04 | 6.54E+04 | 5.32E+04 | 3.47E-01 | -1.53E+00 | down |
| Catechin | Flavanols | 4.68E+06 | 6.61E+06 | 5.65E+06 | 1.89E+06 | 2.11E+06 | 1.84E+06 | 3.45E-01 | -1.54E+00 | down |
| Catechin-(7,8-bc)-4β-(3,4-dihydroxyphenyl)-dihydro-2-(3H)-one | Flavanols | 8.37E+05 | 7.17E+05 | 1.02E+06 | 3.00E+05 | 3.16E+05 | 2.60E+05 | 3.40E-01 | -1.55E+00 | down |
| Diosmetin (5,7,3'-Trihydroxy-4'-methoxyflavone) | Flavonoid | 6.04E+06 | 7.01E+06 | 4.42E+06 | 1.75E+06 | 2.67E+06 | 1.18E+06 | 3.20E-01 | -1.64E+00 | down |
| Quercetin-3-O-(6''-p-Coumaroyl)glucoside | Flavonols | 1.52E+05 | 1.59E+05 | 1.38E+05 | 4.76E+04 | 5.31E+04 | 4.30E+04 | 3.20E-01 | -1.64E+00 | down |
| Kaempferol-3-O-(6''-malonyl)galactoside* | Flavonols | 5.48E+06 | 3.62E+06 | 4.10E+06 | 1.30E+06 | 1.55E+06 | 1.38E+06 | 3.20E-01 | -1.64E+00 | down |
| Sieboldin | Chalcones | 1.12E+05 | 2.17E+05 | 1.76E+05 | 5.58E+04 | 3.65E+04 | 6.46E+04 | 3.11E-01 | -1.69E+00 | down |
| Apigenin | Flavonoid | 9.99E+04 | 1.14E+05 | 6.74E+04 | 2.53E+04 | 3.01E+04 | 2.76E+04 | 2.95E-01 | -1.76E+00 | down |
| Hispidulin (5,7,4'-Trihydroxy-6-methoxyflavone) | Flavonoid | 1.02E+07 | 1.14E+07 | 7.64E+06 | 2.61E+06 | 3.95E+06 | 1.55E+06 | 2.78E-01 | -1.85E+00 | down |
| Catechin-(7,8-bc)-4α-(3,4-dihydroxyphenyl)-dihydro-2-(3H)-one | Flavanols | 6.99E+06 | 1.27E+07 | 1.18E+07 | 2.75E+06 | 3.44E+06 | 2.54E+06 | 2.77E-01 | -1.85E+00 | down |
| Epicatechin | Flavanols | 4.95E+06 | 4.29E+06 | 4.84E+06 | 1.22E+06 | 1.28E+06 | 9.95E+05 | 2.48E-01 | -2.01E+00 | down |
| Quercetin-3-O-(6''-malonyl)galactoside | Flavonols | 9.22E+05 | 5.71E+05 | 5.46E+05 | 1.68E+05 | 2.12E+05 | 1.26E+05 | 2.48E-01 | -2.01E+00 | down |
| Phloretin-4'-O-glucoside (Trilobatin) | Chalcones | 3.42E+05 | 8.82E+05 | 5.26E+05 | 1.39E+05 | 1.68E+05 | 9.45E+04 | 2.29E-01 | -2.13E+00 | down |
| Kaempferol-3-O-(4''-O-p-Coumaroyl)rhamnoside | Flavonoid | 3.93E+07 | 3.84E+07 | 4.00E+07 | 7.52E+06 | 1.03E+07 | 6.96E+06 | 2.10E-01 | -2.25E+00 | down |
| Kaempferol-3-O-(3''-O-p-Coumaroyl)rhamnoside | Flavonoid | 4.29E+07 | 3.97E+07 | 4.55E+07 | 7.78E+06 | 1.03E+07 | 7.04E+06 | 1.96E-01 | -2.35E+00 | down |
| Quercetin-7-O-(6''-malonyl)glucoside | Flavonols | 1.77E+06 | 1.20E+06 | 9.89E+05 | 2.42E+05 | 3.12E+05 | 2.21E+05 | 1.96E-01 | -2.35E+00 | down |
| Quercetin-3-O-(6''-acetyl)galactoside | Flavonols | 3.51E+05 | 2.00E+05 | 2.72E+05 | 4.34E+04 | 6.81E+04 | 4.94E+04 | 1.96E-01 | -2.35E+00 | down |
| Luteolin-7-O-(2''-O-rhamnosyl)rutinoside | Flavonoid | 4.19E+04 | 6.63E+04 | 6.94E+04 | 7.71E+03 | 1.10E+04 | 1.30E+04 | 1.79E-01 | -2.48E+00 | down |
| Quercetin-5,4ʹ-di-O-glucoside | Flavonoid | 1.95E+05 | 1.85E+05 | 2.15E+05 | 3.25E+04 | 4.21E+04 | 2.96E+04 | 1.75E-01 | -2.51E+00 | down |
| Catechin-catechin-catechin | Flavanols | 1.20E+04 | 3.84E+04 | 3.59E+04 | 4.16E+03 | 5.33E+03 | 4.20E+03 | 1.59E-01 | -2.66E+00 | down |
| Aureusidin | Sinensetin | 3.62E+05 | 4.60E+05 | 3.71E+05 | 5.26E+04 | 6.31E+04 | 6.12E+04 | 1.48E-01 | -2.75E+00 | down |
| Myricetin-3-O-glucuronide | Flavonols | 8.19E+06 | 2.80E+07 | 2.29E+07 | 3.47E+06 | 2.40E+06 | 1.14E+06 | 1.19E-01 | -3.08E+00 | down |
| Fisetin | Flavonols | 7.99E+05 | 9.99E+05 | 7.95E+05 | 7.97E+04 | 9.65E+04 | 5.33E+04 | 8.85E-02 | -3.50E+00 | down |
| Kaempferol-3-O-(2''-p-Coumaroyl)glucoside | Flavonoid | 1.37E+06 | 6.96E+05 | 7.81E+05 | 8.35E+04 | 8.18E+04 | 7.93E+04 | 8.58E-02 | -3.54E+00 | down |
| Myricetin-3-O-rhamnoside (Myricitrin) | Flavonols | 7.22E+05 | 2.39E+06 | 1.93E+06 | 1.64E+05 | 1.65E+05 | 9.49E+04 | 8.39E-02 | -3.57E+00 | down |
| Myricetin-3-O-glucoside | Flavonols | 7.10E+05 | 3.98E+06 | 2.82E+06 | 2.40E+05 | 2.53E+05 | 1.31E+05 | 8.32E-02 | -3.59E+00 | down |
| Phloretin | Chalcones | 3.62E+05 | 1.33E+06 | 7.54E+05 | 6.33E+04 | 9.04E+04 | 4.82E+04 | 8.26E-02 | -3.60E+00 | down |
| Kaempferol-3-O-rhamnoside (Afzelin)(Kaempferin) | Flavonols | 8.34E+05 | 1.13E+06 | 8.95E+05 | 6.20E+04 | 7.91E+04 | 5.85E+04 | 6.97E-02 | -3.84E+00 | down |
| Myricetin-3-O-xyloside | Flavonols | 5.37E+04 | 2.86E+05 | 1.89E+05 | 1.63E+04 | 1.32E+04 | 5.77E+03 | 6.67E-02 | -3.91E+00 | down |
| Luteolin-7-O-neohesperidoside (Lonicerin)* | Flavonoid | 4.97E+05 | 1.02E+06 | 7.65E+05 | 1.49E+04 | 1.11E+04 | 1.75E+04 | 1.90E-02 | -5.71E+00 | down |
| Quercetin-3-O-(2''-acetyl)rhamnoside | Flavonols | 1.06E+06 | 1.47E+06 | 1.27E+06 | 2.31E+04 | 2.57E+04 | 2.14E+04 | 1.85E-02 | -5.75E+00 | down |
| Luteolin-7-O-rutinoside* | Flavonoid | 8.32E+05 | 1.69E+06 | 1.17E+06 | 2.01E+04 | 2.69E+04 | 1.89E+04 | 1.78E-02 | -5.81E+00 | down |
| Kaempferol-3-O-(4''-O-acetyl)rhamnoside | Flavonoid | 1.81E+07 | 2.12E+07 | 1.94E+07 | 2.92E+05 | 3.22E+05 | 2.51E+05 | 1.47E-02 | -6.09E+00 | down |
| Luteolin-7-O-(6''-malonyl)glucoside | Flavonoid | 5.09E+03 | 5.63E+03 | 9.04E+03 | 9.00E+00 | 9.00E+00 | 9.00E+00 | 1.37E-03 | -9.52E+00 | down |
| Kaempferol-3-O-sambubioside | Flavonoid | 3.83E+04 | 2.28E+04 | 1.76E+04 | 9.00E+00 | 9.00E+00 | 9.00E+00 | 3.43E-04 | -1.15E+01 | down |
| Hypolaetin | Flavonoid | 7.45E+03 | 7.12E+04 | 3.64E+04 | 9.00E+00 | 9.00E+00 | 9.00E+00 | 2.35E-04 | -1.21E+01 | down |
| Myricetin-3-O-(6''-malony)glucoside | Flavonols | 1.78E+04 | 7.98E+04 | 4.23E+04 | 9.00E+00 | 9.00E+00 | 9.00E+00 | 1.93E-04 | -1.23E+01 | down |
| Cyanidin-3-O-arabinoside | Anthocyanins | 2.37E+04 | 2.85E+04 | 1.76E+05 | 9.00E+00 | 9.00E+00 | 9.00E+00 | 1.18E-04 | -1.30E+01 | down |
| Epicatechin glucoside | Flavanols | 7.67E+04 | 1.42E+05 | 1.10E+05 | 9.00E+00 | 9.00E+00 | 9.00E+00 | 8.21E-05 | -1.36E+01 | down |
| Dihydrocharcone-4'-O-glucoside | Chalcones | 1.73E+05 | 3.82E+05 | 2.22E+05 | 9.00E+00 | 9.00E+00 | 9.00E+00 | 3.47E-05 | -1.48E+01 | down |
| Phloretin-2'-O-glucoside (Phlorizin) | Chalcones | 1.16E+05 | 4.11E+05 | 3.53E+05 | 9.00E+00 | 9.00E+00 | 9.00E+00 | 3.07E-05 | -1.50E+01 | down |
| Tricetin (5,7,3',4',5'-Pentahydroxyflavone) | Flavonoid | 4.28E+05 | 3.54E+05 | 4.95E+05 | 9.00E+00 | 9.00E+00 | 9.00E+00 | 2.12E-05 | -1.55E+01 | down |
| Kaempferol-3-O-arabinoside | Flavonoid | 1.40E+06 | 2.30E+06 | 1.80E+06 | 9.00E+00 | 9.00E+00 | 9.00E+00 | 4.91E-06 | -1.76E+01 | down |

**Table S4 The differental flavonoid metabolites of 9M-ML_vs_10M-ML**

| Compounds | Class II | 9M-ML1 | 9M-ML2 | 9M-ML3 | 10M-ML1 | 10M-ML2 | 10M-ML3 | Fold_Change | Log2FC | Type |
| --- | --- | --- | --- | --- | --- | --- | --- | --- | --- | --- |
| Cyanidin-3-O-arabinoside | Anthocyanins | 9.00E+00 | 9.00E+00 | 9.00E+00 | 1.29E+06 | 1.66E+06 | 7.43E+05 | 1.37E+05 | 1.71E+01 | up |
| Tricetin (5,7,3',4',5'-Pentahydroxyflavone) | Flavonoid | 9.00E+00 | 9.00E+00 | 9.00E+00 | 4.63E+05 | 5.24E+05 | 3.22E+05 | 4.85E+04 | 1.56E+01 | up |
| Epicatechin glucoside | Flavanols | 9.00E+00 | 9.00E+00 | 9.00E+00 | 2.41E+05 | 1.78E+05 | 5.18E+05 | 3.47E+04 | 1.51E+01 | up |
| Kaempferol-3-O-arabinoside | Flavonoid | 9.00E+00 | 9.00E+00 | 9.00E+00 | 2.06E+05 | 2.25E+05 | 3.19E+05 | 2.78E+04 | 1.48E+01 | up |
| Phloretin-2'-O-glucoside (Phlorizin) | Chalcones | 9.00E+00 | 9.00E+00 | 9.00E+00 | 1.59E+05 | 1.21E+05 | 2.10E+05 | 1.81E+04 | 1.41E+01 | up |
| Dihydrocharcone-4'-O-glucoside | Chalcones | 9.00E+00 | 9.00E+00 | 9.00E+00 | 7.59E+04 | 6.63E+04 | 1.12E+05 | 9.41E+03 | 1.32E+01 | up |
| Kaempferol-3-O-sambubioside | Flavonoid | 9.00E+00 | 9.00E+00 | 9.00E+00 | 3.07E+04 | 2.34E+04 | 1.09E+04 | 2.41E+03 | 1.12E+01 | up |
| Luteolin-7-O-(6''-malonyl)glucoside | Flavonoid | 9.00E+00 | 9.00E+00 | 9.00E+00 | 7.01E+03 | 5.13E+03 | 3.39E+03 | 5.75E+02 | 9.17E+00 | up |
| Cyanidin-3-O-glucoside (Kuromanin)* | Anthocyanins | 3.16E+05 | 5.00E+05 | 2.64E+05 | 2.31E+07 | 4.92E+07 | 1.56E+07 | 8.14E+01 | 6.35E+00 | up |
| Catechin-catechin-catechin | Flavanols | 4.16E+03 | 5.33E+03 | 4.20E+03 | 4.72E+04 | 5.01E+04 | 1.38E+05 | 1.72E+01 | 4.10E+00 | up |
| Epicatechin | Flavanols | 1.22E+06 | 1.28E+06 | 9.95E+05 | 6.90E+06 | 7.07E+06 | 1.41E+07 | 8.04E+00 | 3.01E+00 | up |
| Tricin-7-O-(2''-Sinapoyl)glucoside | Flavonoid | 1.26E+04 | 6.62E+03 | 1.25E+04 | 5.76E+04 | 5.74E+04 | 1.21E+05 | 7.45E+00 | 2.90E+00 | up |
| Kaempferol-3-O-(4''-O-acetyl)rhamnoside | Flavonoid | 2.92E+05 | 3.22E+05 | 2.51E+05 | 1.60E+06 | 1.70E+06 | 2.22E+06 | 6.39E+00 | 2.68E+00 | up |
| Catechin | Flavanols | 1.89E+06 | 2.11E+06 | 1.84E+06 | 9.70E+06 | 8.82E+06 | 1.79E+07 | 6.25E+00 | 2.64E+00 | up |
| 7,8-Dihydroxy-5,6,4'-trimethoxyflavone | Flavonoid | 2.38E+05 | 2.74E+05 | 2.50E+05 | 1.59E+06 | 1.34E+06 | 1.61E+06 | 5.97E+00 | 2.58E+00 | up |
| Apigenin | Flavonoid | 2.53E+04 | 3.01E+04 | 2.76E+04 | 1.41E+05 | 1.56E+05 | 1.65E+05 | 5.56E+00 | 2.48E+00 | up |
| Tricin-7-O-Glucoside | Flavonoid | 5.71E+05 | 4.22E+05 | 6.42E+05 | 2.60E+06 | 2.57E+06 | 2.92E+06 | 4.95E+00 | 2.31E+00 | up |
| Naringenin-7-O-glucoside (Prunin) | Dihydroflavone | 6.79E+05 | 9.04E+05 | 7.53E+05 | 4.02E+06 | 3.71E+06 | 3.66E+06 | 4.87E+00 | 2.28E+00 | up |
| Quercetin-3-O-(2''-acetyl)rhamnoside | Flavonols | 2.31E+04 | 2.57E+04 | 2.14E+04 | 9.28E+04 | 8.51E+04 | 1.15E+05 | 4.17E+00 | 2.06E+00 | up |
| Luteolin-7-O-(2''-O-rhamnosyl)rutinoside | Flavonoid | 7.71E+03 | 1.10E+04 | 1.30E+04 | 3.83E+04 | 3.46E+04 | 5.17E+04 | 3.93E+00 | 1.97E+00 | up |
| Quercetin-3-O-rutinoside (Rutin) | Flavonols | 1.12E+06 | 1.83E+06 | 1.19E+06 | 4.36E+06 | 6.55E+06 | 4.42E+06 | 3.70E+00 | 1.89E+00 | up |
| Quercetin-3-O-sambubioside | Flavonols | 3.21E+05 | 4.23E+05 | 3.33E+05 | 1.37E+06 | 1.57E+06 | 1.02E+06 | 3.68E+00 | 1.88E+00 | up |
| 5,8,4'-Trihydroxy-6,7-dimethoxyflavone | Flavonoid | 7.41E+03 | 8.47E+03 | 4.19E+03 | 2.07E+04 | 2.24E+04 | 2.62E+04 | 3.45E+00 | 1.79E+00 | up |
| Quercetin-3-O-(2''-O-galactosyl)glucoside | Flavonols | 8.82E+04 | 8.76E+04 | 9.57E+04 | 3.12E+05 | 3.07E+05 | 2.96E+05 | 3.37E+00 | 1.75E+00 | up |
| Quercetin-3-O-neohesperidoside | Flavonols | 6.85E+06 | 8.47E+06 | 5.75E+06 | 2.23E+07 | 2.64E+07 | 2.03E+07 | 3.27E+00 | 1.71E+00 | up |
| Quercetin-3-O-robinobioside | Flavonols | 1.66E+06 | 2.59E+06 | 1.19E+06 | 5.98E+06 | 6.39E+06 | 4.79E+06 | 3.15E+00 | 1.66E+00 | up |
| Fisetin | Flavonols | 7.97E+04 | 9.65E+04 | 5.33E+04 | 2.31E+05 | 2.14E+05 | 2.77E+05 | 3.15E+00 | 1.66E+00 | up |
| Kaempferol-3,7-O-dirhamnoside (Kaempferitrin) | Flavonols | 1.64E+04 | 2.97E+04 | 1.53E+04 | 4.31E+04 | 4.43E+04 | 1.00E+05 | 3.06E+00 | 1.61E+00 | up |
| Quercetin-3-O-sophoroside (Baimaside) | Flavonols | 4.04E+05 | 4.85E+05 | 4.26E+05 | 1.32E+06 | 1.49E+06 | 1.16E+06 | 3.02E+00 | 1.60E+00 | up |
| Phloretin | Chalcones | 6.33E+04 | 9.04E+04 | 4.82E+04 | 1.29E+05 | 1.64E+05 | 3.17E+05 | 3.02E+00 | 1.59E+00 | up |
| 3-Hydroxy-4',5,7-Trimethoxyflavanone | Flavonoid | 5.22E+04 | 7.29E+04 | 4.96E+04 | 1.60E+05 | 2.04E+05 | 1.53E+05 | 2.96E+00 | 1.56E+00 | up |
| Kaempferol-3-O-rhamnoside (Afzelin)(Kaempferin) | Flavonols | 6.20E+04 | 7.91E+04 | 5.85E+04 | 1.81E+05 | 2.16E+05 | 1.89E+05 | 2.94E+00 | 1.55E+00 | up |
| Tricin (5,7,4'-Trihydroxy-3',5'-dimethoxyflavone) | Flavonoid | 8.90E+06 | 1.09E+07 | 7.88E+06 | 2.62E+07 | 2.93E+07 | 2.41E+07 | 2.87E+00 | 1.52E+00 | up |
| Quercetin-5,4ʹ-di-O-glucoside | Flavonoid | 3.25E+04 | 4.21E+04 | 2.96E+04 | 1.01E+05 | 9.78E+04 | 9.74E+04 | 2.85E+00 | 1.51E+00 | up |
| Catechin-(7,8-bc)-4α-(3,4-dihydroxyphenyl)-dihydro-2-(3H)-one | Flavanols | 2.75E+06 | 3.44E+06 | 2.54E+06 | 7.74E+06 | 6.21E+06 | 1.07E+07 | 2.83E+00 | 1.50E+00 | up |
| Limocitrin-3-O-glucoside | Flavonoid | 4.83E+05 | 4.93E+05 | 3.99E+05 | 1.10E+06 | 1.10E+06 | 1.57E+06 | 2.74E+00 | 1.45E+00 | up |
| Syringetin-7-O-glucoside | Flavonoid | 4.86E+05 | 4.37E+05 | 4.86E+05 | 1.12E+06 | 1.31E+06 | 1.41E+06 | 2.73E+00 | 1.45E+00 | up |
| Quercetin-3-O-(6''-p-Coumaroyl)glucoside | Flavonols | 4.76E+04 | 5.31E+04 | 4.30E+04 | 1.19E+05 | 1.35E+05 | 1.34E+05 | 2.69E+00 | 1.43E+00 | up |
| Quercetin-3-O-(2''-O-glucosyl)glucuronide | Flavonols | 2.78E+05 | 2.99E+05 | 3.01E+05 | 7.43E+05 | 6.80E+05 | 8.82E+05 | 2.63E+00 | 1.39E+00 | up |
| Kaempferol-3-O-(6''-acetyl)glucoside | Flavonols | 3.88E+04 | 3.40E+04 | 4.03E+04 | 7.40E+04 | 1.11E+05 | 1.03E+05 | 2.54E+00 | 1.35E+00 | up |
| Kaempferol-3-O-(4''-O-p-Coumaroyl)rhamnoside | Flavonoid | 7.52E+06 | 1.03E+07 | 6.96E+06 | 1.88E+07 | 2.06E+07 | 2.06E+07 | 2.43E+00 | 1.28E+00 | up |
| Luteolin-7-O-(6''-sinapoyl)glucoside | Flavonoid | 1.01E+04 | 7.45E+03 | 1.82E+04 | 2.61E+04 | 2.80E+04 | 3.23E+04 | 2.42E+00 | 1.28E+00 | up |
| Catechin-(7,8-bc)-4β-(3,4-dihydroxyphenyl)-dihydro-2-(3H)-one | Flavanols | 3.00E+05 | 3.16E+05 | 2.60E+05 | 6.92E+05 | 7.74E+05 | 6.54E+05 | 2.42E+00 | 1.27E+00 | up |
| Iristectorigenin B | Isoflavones | 2.57E+05 | 2.65E+05 | 2.50E+05 | 7.20E+05 | 5.98E+05 | 5.48E+05 | 2.42E+00 | 1.27E+00 | up |
| Kaempferol-3-O-(2''-p-Coumaroyl)glucoside | Flavonoid | 8.35E+04 | 8.18E+04 | 7.93E+04 | 2.02E+05 | 1.90E+05 | 1.98E+05 | 2.41E+00 | 1.27E+00 | up |
| Kaempferol-3-O-(3''-O-p-Coumaroyl)rhamnoside | Flavonoid | 7.78E+06 | 1.03E+07 | 7.04E+06 | 1.91E+07 | 2.05E+07 | 2.06E+07 | 2.40E+00 | 1.26E+00 | up |
| Sieboldin | Chalcones | 5.58E+04 | 3.65E+04 | 6.46E+04 | 1.08E+05 | 9.53E+04 | 1.71E+05 | 2.39E+00 | 1.26E+00 | up |
| Taxifolin-3-O-rhamnoside (Astilbin) | Dihydroflavonol | 2.21E+05 | 2.31E+05 | 1.89E+05 | 5.43E+05 | 6.13E+05 | 3.68E+05 | 2.37E+00 | 1.25E+00 | up |
| Luteolin-7-O-rutinoside* | Flavonoid | 2.01E+04 | 2.69E+04 | 1.89E+04 | 5.29E+04 | 4.86E+04 | 5.28E+04 | 2.34E+00 | 1.23E+00 | up |
| Quercetin-3-O-xyloside (Reynoutrin)* | Flavonols | 3.29E+06 | 3.73E+06 | 2.90E+06 | 7.51E+06 | 7.27E+06 | 8.12E+06 | 2.31E+00 | 1.21E+00 | up |
| Kaempferol-3-O-rutinoside(Nicotiflorin)* | Flavonoid | 7.11E+04 | 9.00E+04 | 7.93E+04 | 1.70E+05 | 2.41E+05 | 1.38E+05 | 2.29E+00 | 1.19E+00 | up |
| Avicularin(Quercetin-3-O-α-L-arabinofuranoside) | Flavonols | 2.82E+06 | 3.18E+06 | 2.65E+06 | 6.72E+06 | 6.22E+06 | 6.80E+06 | 2.28E+00 | 1.19E+00 | up |
| Quercetin-3-O-(6''-p-Coumaroyl)galactoside | Flavonols | 4.18E+04 | 6.54E+04 | 5.32E+04 | 1.16E+05 | 1.25E+05 | 1.22E+05 | 2.26E+00 | 1.18E+00 | up |
| Luteolin (5,7,3',4'-Tetrahydroxyflavone) | Flavonoid | 8.53E+04 | 8.93E+04 | 8.70E+04 | 1.83E+05 | 2.08E+05 | 1.87E+05 | 2.21E+00 | 1.14E+00 | up |
| Luteolin-7-O-glucoside (Cynaroside) | Flavonoid | 2.05E+06 | 2.00E+06 | 2.83E+06 | 5.28E+06 | 5.70E+06 | 3.81E+06 | 2.15E+00 | 1.10E+00 | up |
| Luteolin-7-O-neohesperidoside (Lonicerin)* | Flavonoid | 1.49E+04 | 1.11E+04 | 1.75E+04 | 3.12E+04 | 3.08E+04 | 3.15E+04 | 2.15E+00 | 1.10E+00 | up |
| Luteolin-7-O-(6''-caffeoyl)rhamnoside | Flavonoid | 5.86E+04 | 5.58E+04 | 5.43E+04 | 1.19E+05 | 1.32E+05 | 1.08E+05 | 2.13E+00 | 1.09E+00 | up |
| 6,7,8-Tetrahydroxy-5-methoxyflavone | Flavonoid | 7.66E+04 | 5.44E+04 | 5.34E+04 | 1.34E+05 | 1.27E+05 | 1.26E+05 | 2.10E+00 | 1.07E+00 | up |
| Isorhamnetin-3,7-O-diglucoside | Flavonols | 2.40E+04 | 3.17E+04 | 3.64E+04 | 5.95E+04 | 5.52E+04 | 7.36E+04 | 2.04E+00 | 1.03E+00 | up |
| 7-O-Methyleriodictyol | Flavanols | 2.21E+06 | 1.35E+06 | 1.78E+06 | 3.59E+06 | 3.91E+06 | 3.41E+06 | 2.04E+00 | 1.03E+00 | up |
| Aureusidin | Sinensetin | 5.26E+04 | 6.31E+04 | 6.12E+04 | 9.71E+04 | 1.37E+05 | 1.27E+05 | 2.04E+00 | 1.03E+00 | up |
| Limocitrin-3-O-galactoside | Flavonoid | 1.99E+06 | 2.14E+06 | 1.54E+06 | 7.53E+05 | 6.53E+05 | 8.35E+05 | 3.95E-01 | -1.34E+00 | down |
| Laricitrin-3-O-glucoside | Flavonoid | 8.13E+05 | 7.84E+05 | 6.64E+05 | 2.05E+05 | 1.47E+05 | 2.20E+05 | 2.53E-01 | -1.98E+00 | down |
| 5,4′-Dihydroxy-3,6,7,3′-tetramethoxyflavone | Flavonoid | 1.15E+06 | 1.19E+06 | 1.11E+06 | 2.20E+05 | 1.96E+05 | 2.43E+05 | 1.91E-01 | -2.39E+00 | down |

**Table S5 The differental flavonoid metabolites of 10M-ML_vs_11M-ML**

| Compounds | Class II | 10M-ML1 | 10M-ML2 | 10M-ML3 | 11M-ML1 | 11M-ML2 | 11M-ML3 | Fold_Change | Log2FC | Type |
| --- | --- | --- | --- | --- | --- | --- | --- | --- | --- | --- |
| Catechin-(7,8-bc)-4α-(3,4-dihydroxyphenyl)-dihydro-2-(3H)-one | Flavanols | 7.74E+06 | 6.21E+06 | 1.07E+07 | 2.99E+07 | 4.58E+07 | 1.36E+07 | 3.62E+00 | 1.86E+00 | up |
| Myricetin-3-O-glucuronide | Flavonols | 3.90E+06 | 4.36E+06 | 2.27E+07 | 5.04E+07 | 4.92E+07 | 1.89E+07 | 3.83E+00 | 1.94E+00 | up |
| Kaempferol-3-O-(6''-malonyl)galactoside* | Flavonols | 2.84E+06 | 2.76E+06 | 1.97E+06 | 1.18E+07 | 1.13E+07 | 8.54E+06 | 4.17E+00 | 2.06E+00 | up |
| Kaempferol-3-O-(4''-O-acetyl)rhamnoside | Flavonoid | 1.60E+06 | 1.70E+06 | 2.22E+06 | 2.07E+07 | 1.87E+07 | 8.47E+06 | 8.68E+00 | 3.12E+00 | up |
| Quercetin-7-O-(6''-malonyl)glucoside | Flavonols | 3.97E+05 | 2.98E+05 | 3.97E+05 | 2.59E+06 | 3.50E+06 | 1.13E+06 | 6.62E+00 | 2.73E+00 | up |
| Quercetin-3-O-(6''-malonyl)galactoside | Flavonols | 3.43E+05 | 2.25E+05 | 2.26E+05 | 2.86E+06 | 2.99E+06 | 1.04E+06 | 8.69E+00 | 3.12E+00 | up |
| Myricetin-3-O-glucoside | Flavonols | 3.19E+05 | 2.89E+05 | 2.22E+06 | 1.45E+07 | 1.53E+07 | 2.92E+06 | 1.15E+01 | 3.53E+00 | up |
| Myricetin-3-O-rhamnoside (Myricitrin) | Flavonols | 2.36E+05 | 2.76E+05 | 7.20E+05 | 1.32E+06 | 8.73E+05 | 2.12E+06 | 3.50E+00 | 1.81E+00 | up |
| Fisetin | Flavonols | 2.31E+05 | 2.14E+05 | 2.77E+05 | 8.37E+05 | 6.83E+05 | 4.39E+05 | 2.71E+00 | 1.44E+00 | up |
| Kaempferol-3-O-arabinoside | Flavonoid | 2.06E+05 | 2.25E+05 | 3.19E+05 | 1.35E+06 | 1.18E+06 | 6.24E+05 | 4.21E+00 | 2.08E+00 | up |
| Kaempferol-3-O-rhamnoside (Afzelin)(Kaempferin) | Flavonols | 1.81E+05 | 2.16E+05 | 1.89E+05 | 8.05E+05 | 7.41E+05 | 5.03E+05 | 3.49E+00 | 1.80E+00 | up |
| Quercetin-3-O-(6''-acetyl)galactoside | Flavonols | 1.75E+05 | 5.51E+04 | 1.23E+05 | 1.61E+06 | 1.70E+06 | 3.45E+05 | 1.04E+01 | 3.38E+00 | up |
| Phloretin-2'-O-glucoside (Phlorizin) | Chalcones | 1.59E+05 | 1.21E+05 | 2.10E+05 | 1.71E+06 | 2.23E+06 | 5.48E+05 | 9.16E+00 | 3.20E+00 | up |
| Kaempferol-3-O-(6''-malonyl)glucoside* | Flavonols | 1.35E+05 | 1.48E+05 | 1.03E+05 | 3.29E+05 | 3.40E+05 | 3.06E+05 | 2.53E+00 | 1.34E+00 | up |
| Luteolin-7-O-gentiobioside | Flavonoid | 1.29E+05 | 1.26E+05 | 8.86E+04 | 3.35E+05 | 2.58E+05 | 6.11E+05 | 3.51E+00 | 1.81E+00 | up |
| Phloretin | Chalcones | 1.29E+05 | 1.64E+05 | 3.17E+05 | 1.47E+06 | 2.24E+06 | 7.58E+05 | 7.33E+00 | 2.87E+00 | up |
| Sieboldin | Chalcones | 1.08E+05 | 9.53E+04 | 1.71E+05 | 5.52E+05 | 9.04E+05 | 1.83E+05 | 4.38E+00 | 2.13E+00 | up |
| Dihydroquercetin(Taxifolin) | Dihydroflavonol | 1.02E+05 | 7.53E+04 | 1.68E+05 | 1.30E+06 | 1.59E+06 | 1.82E+05 | 8.91E+00 | 3.16E+00 | up |
| Quercetin-5,4ʹ-di-O-glucoside | Flavonoid | 1.01E+05 | 9.78E+04 | 9.74E+04 | 2.95E+05 | 1.97E+05 | 1.64E+05 | 2.21E+00 | 1.14E+00 | up |
| Gallocatechin | Flavanols | 9.47E+04 | 9.36E+04 | 1.67E+06 | 3.17E+06 | 6.57E+06 | 5.08E+06 | 7.99E+00 | 3.00E+00 | up |
| Quercetin-3-O-(2''-acetyl)rhamnoside | Flavonols | 9.28E+04 | 8.51E+04 | 1.15E+05 | 2.38E+06 | 2.74E+06 | 1.25E+06 | 2.18E+01 | 4.44E+00 | up |
| Phloretin-4'-O-glucoside (Trilobatin) | Chalcones | 7.26E+04 | 1.30E+05 | 2.08E+05 | 3.17E+05 | 4.01E+05 | 5.35E+05 | 3.05E+00 | 1.61E+00 | up |
| Kaempferol-3-O-arabinoside (Juglanin) | Flavonols | 5.97E+04 | 8.39E+04 | 8.86E+04 | 3.51E+05 | 3.15E+05 | 2.03E+05 | 3.74E+00 | 1.90E+00 | up |
| Kaempferol-3,7-di-O-glucoside | Flavonoid | 5.78E+04 | 3.73E+04 | 4.09E+04 | 1.58E+05 | 1.43E+05 | 2.24E+05 | 3.86E+00 | 1.95E+00 | up |
| Luteolin-7-O-rutinoside* | Flavonoid | 5.29E+04 | 4.86E+04 | 5.28E+04 | 8.07E+05 | 5.68E+05 | 8.94E+05 | 1.47E+01 | 3.88E+00 | up |
| Myricetin-3-O-xyloside | Flavonols | 3.21E+04 | 3.73E+04 | 1.73E+05 | 5.31E+05 | 8.74E+05 | 1.57E+05 | 6.44E+00 | 2.69E+00 | up |
| Luteolin-7-O-neohesperidoside (Lonicerin)* | Flavonoid | 3.12E+04 | 3.08E+04 | 3.15E+04 | 4.48E+05 | 3.37E+05 | 5.60E+05 | 1.44E+01 | 3.85E+00 | up |
| Kaempferol-3-O-sambubioside | Flavonoid | 3.07E+04 | 2.34E+04 | 1.09E+04 | 6.23E+04 | 4.19E+04 | 2.09E+05 | 4.81E+00 | 2.27E+00 | up |
| 2'-Hydroxy-3,4,5,4',6'-pentamethoxychalcone | Chalcones | 2.80E+04 | 1.39E+04 | 2.39E+04 | 2.20E+05 | 1.75E+05 | 2.49E+05 | 9.78E+00 | 3.29E+00 | up |
| Luteolin-7-O-(6''-sinapoyl)glucoside | Flavonoid | 2.61E+04 | 2.80E+04 | 3.23E+04 | 3.73E+05 | 1.46E+05 | 1.01E+05 | 7.17E+00 | 2.84E+00 | up |
| Myricetin-3-O-arabinoside | Flavonols | 2.59E+04 | 3.10E+04 | 2.07E+05 | 5.46E+05 | 7.90E+05 | 1.98E+05 | 5.81E+00 | 2.54E+00 | up |
| Pinobanksin* | Dihydroflavonol | 1.58E+04 | 3.79E+04 | 4.36E+04 | 9.02E+04 | 1.24E+05 | 1.05E+05 | 3.28E+00 | 1.72E+00 | up |
| Luteolin-7-O-(6''-malonyl)glucoside | Flavonoid | 7.01E+03 | 5.13E+03 | 3.39E+03 | 1.67E+04 | 1.56E+04 | 1.21E+04 | 2.86E+00 | 1.52E+00 | up |
| Tricin (5,7,4'-Trihydroxy-3',5'-dimethoxyflavone) | Flavonoid | 2.62E+07 | 2.93E+07 | 2.41E+07 | 5.39E+06 | 5.75E+06 | 7.08E+06 | 2.29E-01 | -2.13E+00 | down |
| Cyanidin-3-O-glucoside (Kuromanin)* | Anthocyanins | 2.31E+07 | 4.92E+07 | 1.56E+07 | 1.09E+06 | 1.34E+06 | 2.01E+05 | 2.99E-02 | -5.06E+00 | down |
| Quercetin-3-O-neohesperidoside | Flavonols | 2.23E+07 | 2.64E+07 | 2.03E+07 | 4.76E+05 | 3.97E+05 | 4.11E+05 | 1.86E-02 | -5.75E+00 | down |
| 5,7,4'-Trihydroxy-6,8-dimethoxyisoflavone-7-O-galactoside | Isoflavones | 8.19E+06 | 8.16E+06 | 9.86E+06 | 2.14E+06 | 2.10E+06 | 3.81E+06 | 3.07E-01 | -1.70E+00 | down |
| Quercetin-3-O-robinobioside | Flavonols | 5.98E+06 | 6.39E+06 | 4.79E+06 | 9.16E+04 | 1.09E+05 | 1.20E+05 | 1.87E-02 | -5.74E+00 | down |
| Quercetin-3-O-rhamnoside(Quercitrin) | Flavonols | 5.39E+06 | 6.07E+06 | 5.20E+06 | 1.61E+06 | 8.06E+05 | 1.27E+06 | 2.21E-01 | -2.18E+00 | down |
| Quercetin-3-O-rutinoside (Rutin) | Flavonols | 4.36E+06 | 6.55E+06 | 4.42E+06 | 6.38E+04 | 7.96E+04 | 8.80E+04 | 1.51E-02 | -6.05E+00 | down |
| Apigenin-6-C-glucoside (Isovitexin) | Flavonoid carbonoside | 3.80E+06 | 3.82E+06 | 4.28E+06 | 5.55E+05 | 5.17E+05 | 9.31E+05 | 1.68E-01 | -2.57E+00 | down |
| Kaempferol-3-O-neohesperidoside* | Flavonols | 2.99E+06 | 3.20E+06 | 2.57E+06 | 3.35E+04 | 3.78E+04 | 7.85E+04 | 1.71E-02 | -5.87E+00 | down |
| Tricin-7-O-Glucoside | Flavonoid | 2.60E+06 | 2.57E+06 | 2.92E+06 | 8.79E+04 | 7.05E+04 | 7.91E+04 | 2.93E-02 | -5.09E+00 | down |
| 7,8-Dihydroxy-5,6,4'-trimethoxyflavone | Flavonoid | 1.59E+06 | 1.34E+06 | 1.61E+06 | 9.69E+03 | 7.44E+03 | 5.58E+04 | 1.60E-02 | -5.96E+00 | down |
| 5,2'-Dihydroxy-7,8-dimethoxyflavone glycosides | Flavonoid | 1.48E+06 | 1.37E+06 | 1.63E+06 | 3.39E+04 | 2.97E+04 | 8.87E+04 | 3.40E-02 | -4.88E+00 | down |
| Quercetin-3-O-sambubioside | Flavonols | 1.37E+06 | 1.57E+06 | 1.02E+06 | 1.20E+05 | 1.11E+05 | 4.45E+05 | 1.70E-01 | -2.56E+00 | down |
| 5,7-Dihydroxy-3',4',5'-trimethoxyflavone | Flavonoid | 1.07E+06 | 1.10E+06 | 1.08E+06 | 7.20E+04 | 6.34E+04 | 5.29E+05 | 2.05E-01 | -2.29E+00 | down |
| Iristectorigenin B | Isoflavones | 7.20E+05 | 5.98E+05 | 5.48E+05 | 2.16E+05 | 2.44E+05 | 4.87E+04 | 2.72E-01 | -1.88E+00 | down |
| Tricetin (5,7,3',4',5'-Pentahydroxyflavone) | Flavonoid | 4.63E+05 | 5.24E+05 | 3.22E+05 | 9.00E+00 | 9.00E+00 | 9.00E+00 | 2.06E-05 | -1.56E+01 | down |
| Apigenin-5-O-glucoside | Flavonoid | 2.28E+05 | 2.36E+05 | 2.19E+05 | 5.69E+04 | 4.75E+04 | 5.47E+04 | 2.33E-01 | -2.10E+00 | down |
| Luteolin (5,7,3',4'-Tetrahydroxyflavone) | Flavonoid | 1.83E+05 | 2.08E+05 | 1.87E+05 | 7.00E+04 | 7.56E+04 | 3.35E+04 | 3.10E-01 | -1.69E+00 | down |
| Kaempferol-3-O-rutinoside(Nicotiflorin)* | Flavonoid | 1.70E+05 | 2.41E+05 | 1.38E+05 | 9.00E+00 | 9.00E+00 | 9.00E+00 | 4.91E-05 | -1.43E+01 | down |
| 3-Hydroxy-4',5,7-Trimethoxyflavanone | Flavonoid | 1.60E+05 | 2.04E+05 | 1.53E+05 | 3.29E+04 | 3.51E+04 | 5.07E+04 | 2.30E-01 | -2.12E+00 | down |
| Naringenin-7-O-Rutinoside(Narirutin)* | Dihydroflavone | 1.42E+05 | 1.62E+05 | 1.06E+05 | 9.00E+00 | 9.00E+00 | 1.46E+04 | 3.55E-02 | -4.82E+00 | down |
| Apigenin | Flavonoid | 1.41E+05 | 1.56E+05 | 1.65E+05 | 5.58E+04 | 4.33E+04 | 2.24E+04 | 2.63E-01 | -1.92E+00 | down |
| 6,7,8-Tetrahydroxy-5-methoxyflavone | Flavonoid | 1.34E+05 | 1.27E+05 | 1.26E+05 | 9.00E+00 | 9.00E+00 | 3.28E+04 | 8.47E-02 | -3.56E+00 | down |
| Naringenin-7-O-Neohesperidoside(Naringin)* | Dihydroflavone | 1.34E+05 | 1.64E+05 | 1.06E+05 | 9.00E+00 | 9.00E+00 | 9.00E+00 | 6.68E-05 | -1.39E+01 | down |
| 3-O-Methylquercetin | Flavonols | 6.02E+04 | 4.99E+04 | 6.17E+04 | 1.25E+04 | 1.04E+04 | 1.04E+04 | 1.94E-01 | -2.37E+00 | down |
| Tricin-7-O-(2''-Sinapoyl)glucoside | Flavonoid | 5.76E+04 | 5.74E+04 | 1.21E+05 | 2.42E+04 | 1.53E+04 | 2.00E+04 | 2.51E-01 | -1.99E+00 | down |
| Acacetin-7-O-glucoside (Tilianin)* | Flavonoid | 5.00E+04 | 4.90E+04 | 5.62E+04 | 1.04E+04 | 1.64E+04 | 2.84E+04 | 3.55E-01 | -1.49E+00 | down |
| 4',5-Dihydroxy-3',5'-dimethoxyflavone | Flavonoid | 2.96E+04 | 2.12E+04 | 2.28E+04 | 9.00E+00 | 9.00E+00 | 9.00E+00 | 3.67E-04 | -1.14E+01 | down |
| 3’,4’-Dihydroxy-7,5’-dimethoxyflavone | Flavonoid | 1.13E+04 | 1.44E+04 | 1.36E+04 | 9.00E+00 | 9.00E+00 | 3.35E+03 | 8.57E-02 | -3.54E+00 | down |

**Table S9 The expression of function genes related with flavonoid synthesis in *C. paliurus***

| **Pfam_annotation** | **8M Average** | **8M STDEV** | **9M Average** | **9M STDEV** | **10M Average** | **10M STDEV** | **11M Average** | **11M STDEV** |
| --- | --- | --- | --- | --- | --- | --- | --- | --- |
| 5-O-(4-coumaroyl)-D-quinate 3'-monooxygenase_1 | 3.31E-01 | 3.06E-01 | 0.00E+00 | 0.00E+00 | 3.65E-01 | 6.32E-01 | 2.27E-01 | 3.93E-01 |
| flavonol synthase_1 | 6.26E+01 | 1.84E+00 | 4.05E+01 | 3.69E+01 | 3.12E+01 | 3.11E+01 | 6.57E+01 | 1.02E+01 |
| anthocyanidin 3-O-glucosyltransferase_2 | 8.47E+01 | 6.50E+01 | 2.40E+02 | 2.60E+01 | 1.51E+02 | 8.37E+01 | 0.00E+00 | 0.00E+00 |
| 5-O-(4-coumaroyl)-D-quinate 3'-monooxygenase_2 | 2.18E+00 | 6.62E-01 | 8.54E-01 | 1.07E+00 | 2.24E-01 | 3.88E-01 | 5.99E-01 | 6.65E-01 |
| 5-O-(4-coumaroyl)-D-quinate 3'-monooxygenase_3 | 8.71E+00 | 1.35E+01 | 4.57E+00 | 7.58E+00 | 8.80E+00 | 1.52E+01 | 1.05E+00 | 5.17E-01 |
| Chalcone and stilbene synthases_1 | 4.60E+01 | 2.01E+00 | 8.90E-01 | 1.42E+00 | 2.75E+01 | 3.88E+01 | 2.14E+00 | 5.83E-01 |
| leucoanthocyanidin reductase | 1.76E-01 | 1.54E-01 | 0.00E+00 | 0.00E+00 | 1.37E-02 | 2.38E-02 | 3.05E-02 | 5.28E-02 |
| trans-cinnamate 4-monooxygenase_1 | 2.33E+01 | 1.21E+01 | 9.24E-01 | 1.33E+00 | 5.31E+00 | 9.19E+00 | 2.14E+01 | 6.60E+00 |
| chalcone isomerase_1 | 1.69E+01 | 4.39E-01 | 5.77E+00 | 4.63E+00 | 3.86E+00 | 6.69E+00 | 9.97E+00 | 7.60E+00 |
| flavonol synthase_2 | 5.88E+01 | 1.17E+01 | 4.53E+01 | 1.22E+01 | 4.55E+01 | 4.08E+01 | 4.77E+01 | 4.01E+00 |
| flavone synthase II | 9.64E+00 | 6.74E+00 | 4.52E+00 | 1.85E+00 | 1.81E+00 | 3.11E+00 | 9.59E+00 | 6.63E+00 |
| flavone synthase II | 4.62E+01 | 1.55E+00 | 4.64E+01 | 6.19E+00 | 6.40E+01 | 4.98E+00 | 5.36E+01 | 4.77E+00 |
| trans-cinnamate 4-monooxygenase_2 | 1.37E+01 | 3.81E-01 | 1.19E+01 | 1.25E+00 | 9.24E+00 | 1.16E+01 | 1.72E+01 | 8.69E-01 |
| flavanone 7-O-glucoside 2''-O-beta-L-rhamnosyltransferase | 2.57E+00 | 2.23E+00 | 7.95E-01 | 9.77E-01 | 1.11E+00 | 1.92E+00 | 4.13E-02 | 3.84E-02 |
| flavonoid 3',5'-hydroxylase | 3.36E+00 | 8.28E-01 | 3.55E+00 | 2.59E+00 | 9.64E-01 | 1.67E+00 | 6.64E+00 | 4.39E+00 |
| trans-cinnamate 4-monooxygenase_3 | 1.27E+01 | 2.47E+00 | 6.07E+00 | 1.74E+00 | 6.90E+00 | 1.19E+01 | 2.63E+01 | 3.61E+00 |
| anthocyanidin reductase_1 | 2.20E+01 | 3.41E+00 | 4.68E+00 | 6.23E+00 | 2.27E+01 | 3.94E+01 | 1.19E+00 | 5.90E-01 |
| Chalcone and stilbene synthases_2 | 1.35E+02 | 6.16E+00 | 0.00E+00 | 0.00E+00 | 1.22E+02 | 1.12E+02 | 1.20E+01 | 3.49E+00 |
| shikimate O-hydroxycinnamoyltransferase_1 | 2.47E+00 | 4.51E-01 | 1.51E+00 | 7.52E-01 | 4.67E-01 | 8.09E-01 | 1.37E+00 | 4.56E-01 |
| shikimate O-hydroxycinnamoyltransferase_2 | 3.02E+00 | 2.96E-01 | 3.68E+00 | 3.45E-01 | 8.49E-01 | 1.47E+00 | 2.93E+00 | 1.92E+00 |
| chalcone isomerase_2 | 8.10E-01 | 2.05E-01 | 1.85E+00 | 1.12E+00 | 5.92E-01 | 1.03E+00 | 9.54E+00 | 7.72E+00 |
| Chalcone and stilbene synthases_3 | 8.91E-01 | 1.25E+00 | 8.33E-01 | 1.18E+00 | 5.90E-01 | 1.02E+00 | 1.38E+00 | 4.33E-01 |
| naringenin 3-dioxygenase | 2.12E+01 | 6.59E+00 | 2.75E+01 | 2.65E+00 | 1.33E+01 | 2.31E+01 | 1.59E+01 | 1.23E+00 |
| shikimate O-hydroxycinnamoyltransferase_3 | 2.38E-02 | 4.13E-02 | 0.00E+00 | 0.00E+00 | 9.94E-03 | 1.72E-02 | 0.00E+00 | 0.00E+00 |
| shikimate O-hydroxycinnamoyltransferase_4 | 3.39E+00 | 3.09E+00 | 3.89E+00 | 5.02E+00 | 6.63E-01 | 1.15E+00 | 0.00E+00 | 0.00E+00 |
| flavanone 4-reductase_1 | 8.22E-01 | 6.38E-01 | 9.02E-01 | 5.60E-01 | 1.93E-01 | 3.35E-01 | 2.21E+00 | 9.85E-01 |
| flavanone 4-reductase_2 | 1.49E+00 | 1.31E+00 | 3.77E+00 | 1.82E+00 | 3.51E+00 | 3.93E+00 | 1.09E-01 | 1.89E-01 |
| chalcone isomerase_3 | 6.87E+00 | 4.73E-01 | 5.15E+00 | 1.58E+00 | 2.38E+00 | 4.13E+00 | 3.95E+00 | 1.37E+00 |
| Chalcone and stilbene synthases_4 | 5.34E+00 | 1.24E+00 | 6.25E+00 | 1.58E+00 | 1.79E+00 | 3.10E+00 | 1.29E+01 | 3.93E+00 |
| phlorizin synthase_1 | 1.40E+00 | 1.11E+00 | 1.05E+00 | 2.66E-01 | 5.90E-01 | 1.02E+00 | 1.49E+01 | 3.71E+00 |
| phlorizin synthase_2 | 4.78E+00 | 3.13E-01 | 3.09E+00 | 4.62E-01 | 7.84E-01 | 1.36E+00 | 0.00E+00 | 0.00E+00 |
| flavonol synthase_3 | 4.82E+00 | 5.55E+00 | 1.34E+00 | 1.59E+00 | 1.76E+00 | 1.87E+00 | 1.37E+01 | 7.31E+00 |
| anthocyanidin reductase_2 | 5.21E+00 | 6.35E-01 | 4.89E+00 | 1.58E+00 | 1.83E+00 | 3.17E+00 | 2.74E-01 | 2.42E-01 |
| anthocyanidin reductase_3 | 1.96E+00 | 2.95E+00 | 1.22E+00 | 3.03E-01 | 2.96E-01 | 5.12E-01 | 1.00E-02 | 1.73E-02 |
| anthocyanidin reductase_4 | 2.19E+00 | 2.91E+00 | 4.30E-01 | 5.62E-01 | 5.74E-01 | 9.95E-01 | 1.05E-01 | 1.83E-01 |
| flavanone 4-reductase_3 | 3.18E-01 | 1.07E-01 | 1.22E-01 | 2.10E-01 | 0.00E+00 | 0.00E+00 | 9.00E-03 | 1.56E-02 |
| Chalcone and stilbene synthases_5 | 0.00E+00 | 0.00E+00 | 2.33E-02 | 4.04E-02 | 0.00E+00 | 0.00E+00 | 0.00E+00 | 0.00E+00 |
| shikimate O-hydroxycinnamoyltransferase_5 | 5.00E+00 | 2.98E+00 | 5.83E+00 | 4.17E+00 | 1.51E+00 | 2.62E+00 | 7.13E+00 | 1.89E+00 |
| shikimate O-hydroxycinnamoyltransferase_6 | 8.38E+00 | 1.34E+00 | 7.58E+00 | 4.15E+00 | 4.26E+00 | 7.37E+00 | 1.41E+01 | 4.37E+00 |
| shikimate O-hydroxycinnamoyltransferase_7 | 5.69E+00 | 7.05E-01 | 3.49E+00 | 8.95E-01 | 2.42E+00 | 2.13E+00 | 5.52E+00 | 3.43E+00 |
| shikimate O-hydroxycinnamoyltransferase_8 | 4.27E-01 | 4.47E-01 | 3.22E-01 | 2.07E-01 | 3.07E-01 | 5.32E-01 | 0.00E+00 | 0.00E+00 |
| shikimate O-hydroxycinnamoyltransferase_9 | 0.00E+00 | 0.00E+00 | 0.00E+00 | 0.00E+00 | 0.00E+00 | 0.00E+00 | 5.16E+01 | 2.11E+01 |
| chalcone isomerase_4 | 5.19E-01 | 3.98E-01 | 1.25E+00 | 1.03E+00 | 1.82E+00 | 1.66E+00 | 7.73E-01 | 1.41E-01 |
| anthocyanidin synthase_1 | 0.00E+00 | 0.00E+00 | 0.00E+00 | 0.00E+00 | 0.00E+00 | 0.00E+00 | 1.95E-01 | 3.37E-01 |
| shikimate O-hydroxycinnamoyltransferase_10 | 1.38E-01 | 1.64E-02 | 4.38E-02 | 6.66E-02 | 2.43E-02 | 4.22E-02 | 2.49E-02 | 4.32E-02 |
| anthocyanidin 3-O-glucosyltransferase_1 | 6.51E-02 | 1.13E-01 | 1.68E-01 | 2.31E-01 | 2.77E-02 | 4.79E-02 | 0.00E+00 | 0.00E+00 |
| shikimate O-hydroxycinnamoyltransferase_11 | 2.82E+01 | 2.95E+00 | 4.47E+01 | 2.00E+01 | 1.24E+01 | 7.34E+00 | 5.04E+00 | 7.05E-01 |
| shikimate O-hydroxycinnamoyltransferase_12 | 2.97E+00 | 2.65E+00 | 1.92E+01 | 4.69E+00 | 0.00E+00 | 0.00E+00 | 3.24E+00 | 3.00E+00 |
| shikimate O-hydroxycinnamoyltransferase_13 | 2.79E+01 | 3.43E+00 | 1.53E+01 | 3.53E+00 | 8.23E+00 | 9.41E+00 | 0.00E+00 | 0.00E+00 |
| shikimate O-hydroxycinnamoyltransferase_14 | 2.55E+00 | 2.82E+00 | 2.01E+00 | 2.01E+00 | 1.78E+00 | 3.08E+00 | 1.70E-01 | 2.04E-02 |
| shikimate O-hydroxycinnamoyltransferase_15 | 7.38E+01 | 2.80E+01 | 8.52E+01 | 5.45E+00 | 2.51E+01 | 4.35E+01 | 6.64E+01 | 1.38E+00 |
| chalcone isomerase_5 | 1.14E+01 | 1.07E+00 | 1.39E+01 | 3.14E+00 | 1.31E+01 | 2.27E+01 | 8.76E+01 | 3.83E+01 |
| flavonol synthase_4 | 6.09E+00 | 2.05E+00 | 6.83E+00 | 6.46E+00 | 2.02E+00 | 3.50E+00 | 2.62E+00 | 3.36E+00 |
| phlorizin synthase_3 | 3.39E+00 | 3.09E+00 | 3.89E+00 | 5.02E+00 | 6.63E-01 | 1.15E+00 | 0.00E+00 | 0.00E+00 |
| flavonoid 3'-monooxygenase | 8.22E-01 | 6.38E-01 | 9.02E-01 | 5.60E-01 | 1.93E-01 | 3.35E-01 | 2.21E+00 | 9.85E-01 |
| flavonoid 3'-monooxygenase | 4.78E+01 | 1.88E+00 | 4.53E+01 | 3.73E+00 | 1.29E+01 | 2.22E+01 | 7.15E+00 | 1.54E+00 |
| shikimate O-hydroxycinnamoyltransferase_16 | 2.46E+01 | 1.30E+00 | 4.05E+01 | 9.98E+00 | 9.83E+00 | 1.70E+01 | 6.72E+00 | 5.86E+00 |
| anthocyanidin synthase_2 | 2.70E+00 | 9.06E-01 | 3.56E+00 | 5.35E-01 | 7.87E-01 | 1.36E+00 | 1.99E-01 | 3.80E-02 |
| chalcone isomerase_6 | 9.18E-01 | 1.24E-01 | 1.47E-01 | 2.55E-01 | 1.71E+00 | 2.95E+00 | 4.77E-01 | 1.84E-01 |
| anthocyanidin reductase_5 | 4.83E+00 | 7.52E-01 | 6.33E+00 | 7.30E-01 | 2.46E+00 | 2.71E+00 | 1.25E-01 | 2.25E-02 |
| chalcone isomerase_7 ( | 2.74E+00 | 6.88E-01 | 1.76E+00 | 1.64E+00 | 6.26E-01 | 1.08E+00 | 0.00E+00 | 0.00E+00 |
| anthocyanidin synthase_3 | 8.13E+00 | 4.82E-01 | 8.21E-01 | 1.63E-01 | 1.95E+00 | 3.39E+00 | 7.81E+00 | 3.03E+00 |
| shikimate O-hydroxycinnamoyltransferase_17 | 2.89E-01 | 2.69E-01 | 4.69E-02 | 8.12E-02 | 9.01E-02 | 1.56E-01 | 9.91E-01 | 5.84E-01 |
| shikimate O-hydroxycinnamoyltransferase_18 | 1.98E+01 | 2.35E+00 | 1.25E+01 | 8.02E+00 | 1.26E+01 | 2.18E+01 | 9.13E+00 | 1.48E+00 |
| chalcone isomerase_8 | 3.21E+00 | 4.24E-01 | 1.42E+00 | 1.24E+00 | 1.66E+00 | 2.87E+00 | 3.82E+00 | 2.14E+00 |
| shikimate O-hydroxycinnamoyltransferase_19 | 3.66E+00 | 1.81E+00 | 4.48E-01 | 4.59E-01 | 6.99E-01 | 1.21E+00 | 0.00E+00 | 0.00E+00 |

**Table S10 The expression of significantly different transcription factors in *DEGs***

| **Pfam_annotation** | **8M Average** | **8M STDEV** | **9M Average** | **9M STDEV** | **10M Average** | **10M STDEV** | **11M Average** | **11M STDEV** |
| --- | --- | --- | --- | --- | --- | --- | --- | --- |
| mTERF | 1.74E+00 | 2.77E-01 | 4.38E+00 | 1.96E+00 | 4.26E-01 | 7.39E-01 | 1.17E+00 | 6.47E-01 |
| KIX domain | 1.73E+00 | 6.45E-01 | 9.75E-01 | 2.16E-01 | 4.72E-01 | 8.17E-01 | 4.63E-01 | 2.47E-01 |
| SRF-type transcription factor | 2.84E+01 | 9.11E-01 | 2.81E+01 | 2.76E+00 | 1.02E+01 | 1.72E+01 | 1.75E+01 | 1.10E+00 |
| bHLH-MYC and R2R3-MYB transcription factors N-terminal | 8.52E+00 | 2.53E-01 | 0.00E+00 | 0.00E+00 | 0.00E+00 | 0.00E+00 | 0.00E+00 | 0.00E+00 |
| AP2-like factor | 1.47E+01 | 1.26E+00 | 1.34E+01 | 4.78E-01 | 4.58E+00 | 5.84E+00 | 2.42E+00 | 9.46E-01 |
| GATA zinc finger | 6.37E+00 | 6.98E-01 | 9.55E+00 | 1.11E+00 | 1.91E+00 | 3.32E+00 | 7.63E-01 | 5.78E-01 |
| WRKY DNA -binding domain | 1.70E+00 | 3.75E-01 | 1.93E+00 | 9.09E-01 | 1.22E+00 | 2.12E+00 | 7.52E+00 | 2.68E+00 |
| Plant protein of unknown function | 2.76E-01 | 2.48E-01 | 7.82E-02 | 7.38E-02 | 0.00E+00 | 0.00E+00 | 1.72E+00 | 9.38E-01 |
| Myb-like DNA-binding domain | 1.32E+02 | 8.55E+00 | 0.00E+00 | 0.00E+00 | 0.00E+00 | 0.00E+00 | 0.00E+00 | 0.00E+00 |
| Myb-like DNA-binding domain | 1.22E+00 | 4.70E-01 | 3.44E-01 | 2.95E-01 | 3.29E-01 | 5.70E-01 | 1.67E+00 | 2.50E-01 |
| Plectin/S10 domain | 3.83E+01 | 2.95E+00 | 7.08E+01 | 1.58E+01 | 1.93E+01 | 3.34E+01 | 2.67E+01 | 6.04E+00 |
| Smg-4/UPF3 family | 8.03E+00 | 3.82E-02 | 8.72E+00 | 6.40E-01 | 4.08E+00 | 4.44E+00 | 1.25E+01 | 2.76E+00 |
| Histone-like transcription factor (CBF/NF-Y) | 5.44E+00 | 1.13E+00 | 1.98E+00 | 4.75E-01 | 3.97E+00 | 3.68E+00 | 5.79E+00 | 1.50E-01 |
| Zinc finger, C2H2 type | 4.66E-01 | 2.33E-01 | 3.69E-01 | 7.10E-02 | 1.33E-02 | 2.31E-02 | 0.00E+00 | 0.00E+00 |
| Seed dormancy control | 1.90E+00 | 1.27E-01 | 8.03E-01 | 9.04E-02 | 1.04E+00 | 9.00E-01 | 4.83E+00 | 6.47E-01 |
| bZIP transcription factor | 5.84E-01 | 1.29E-01 | 3.07E+00 | 4.16E-01 | 8.27E-01 | 1.43E+00 | 0.00E+00 | 0.00E+00 |
| WRKY DNA -binding domain | 0.00E+00 | 0.00E+00 | 0.00E+00 | 0.00E+00 | 3.71E-02 | 6.42E-02 | 5.14E-01 | 9.83E-02 |
| Transcription factor S-II (TFIIS) | 5.09E+00 | 3.97E+00 | 1.36E+00 | 5.78E-01 | 6.51E-01 | 1.13E+00 | 1.20E+01 | 7.73E+00 |
| WD domain, G-beta repeat | 3.02E+01 | 1.77E+00 | 1.94E+01 | 1.28E+00 | 4.50E+01 | 2.79E+01 | 1.66E+01 | 3.03E-01 |
| Myb | 1.39E-02 | 2.41E-02 | 4.72E-01 | 2.63E-01 | 1.72E-01 | 2.98E-01 | 1.13E-01 | 8.85E-02 |
| AP2 domain | 1.69E+00 | 3.92E-01 | 0.00E+00 | 0.00E+00 | 4.09E-01 | 7.08E-01 | 0.00E+00 | 0.00E+00 |
| Helix-loop-helix DNA-binding domain | 1.58E+00 | 9.23E-02 | 2.91E+00 | 1.86E-01 | 4.07E-01 | 7.04E-01 | 1.21E+00 | 9.03E-01 |
| TFIIE alpha subunit | 5.78E+00 | 8.93E-01 | 5.04E+00 | 4.49E-01 | 1.90E+00 | 3.29E+00 | 7.52E+00 | 1.17E+00 |
| linker histone H1 and H5 family | 3.33E+01 | 1.91E+00 | 2.35E+01 | 4.69E+00 | 1.87E+01 | 9.89E+00 | 4.26E+01 | 8.17E+00 |
| AP2-like factor | 4.64E+00 | 1.34E+00 | 1.33E+00 | 6.17E-01 | 2.22E+00 | 2.22E+00 | 8.42E-02 | 8.19E-02 |
| BHLH | 1.99E+00 | 4.95E-01 | 3.10E+00 | 4.46E-01 | 3.15E+00 | 3.71E+00 | 5.63E-02 | 9.74E-02 |
| RecQ zinc-binding | 3.52E+00 | 4.74E-01 | 1.79E+00 | 5.01E-01 | 6.86E-01 | 1.19E+00 | 3.27E+00 | 4.30E-01 |
| Transcription initiation factor TFIID subunit A | 2.43E+00 | 1.26E+00 | 1.87E+00 | 6.77E-01 | 5.58E-01 | 9.66E-01 | 4.65E+00 | 6.81E-01 |
| Myb-like DNA-binding domain | 4.66E+00 | 5.50E-01 | 1.75E+00 | 1.00E-01 | 1.44E+00 | 2.49E+00 | 4.20E+00 | 1.89E+00 |
| Myb-like DNA-binding domain | 3.28E+00 | 2.56E+00 | 2.50E-01 | 2.25E-01 | 1.53E+00 | 2.65E+00 | 5.20E-02 | 5.38E-02 |
| Myb | 1.76E+01 | 8.16E-01 | 2.25E+01 | 8.39E+00 | 5.56E+00 | 7.62E+00 | 6.47E+00 | 5.28E-01 |
| Myb-like DNA-binding domain | 1.25E+00 | 4.22E-01 | 1.52E+00 | 6.93E-01 | 2.52E-02 | 4.36E-02 | 2.74E-02 | 4.74E-02 |
| KOW motif | 8.78E+00 | 2.01E-01 | 6.31E+00 | 8.63E-01 | 1.66E+00 | 2.88E+00 | 1.22E+01 | 2.24E+00 |
| PAX-interacting protein 1 | 5.33E-01 | 2.46E-01 | 3.32E-01 | 2.88E-01 | 2.35E-01 | 4.07E-01 | 0.00E+00 | 0.00E+00 |
| heat shock transcription factor | 6.85E+00 | 4.83E+00 | 7.48E-01 | 2.98E-01 | 3.98E+00 | 6.89E+00 | 6.01E+00 | 1.92E+00 |
| heat shock transcription factor | 4.23E+00 | 1.03E+00 | 2.24E+00 | 7.44E-01 | 1.69E+00 | 1.88E+00 | 4.56E+00 | 7.81E-01 |
| Myb | 1.39E+00 | 9.83E-01 | 1.59E+00 | 2.66E-01 | 5.73E-01 | 9.93E-01 | 2.79E-01 | 2.39E-01 |
| TFIIH subunit 1 | 1.23E+00 | 4.78E-01 | 6.30E-01 | 2.49E-01 | 5.27E-01 | 9.14E-01 | 8.40E-02 | 6.65E-02 |
| WRKY DNA -binding domain | 1.65E+00 | 9.93E-03 | 1.20E+00 | 2.74E-01 | 4.69E-01 | 8.12E-01 | 8.95E+00 | 1.17E+00 |
| AP2 domain | 7.91E+01 | 1.76E+00 | 2.97E+01 | 2.58E+01 | 2.05E+01 | 3.54E+01 | 1.06E+02 | 6.94E+00 |
| Targeting protein for Xklp2 (TPX2) domain | 5.64E+00 | 3.04E+00 | 1.02E+01 | 3.79E+00 | 7.16E+00 | 5.86E+00 | 1.97E-01 | 2.86E-01 |
| Helix-loop-helix DNA-binding domain | 1.03E+00 | 9.47E-01 | 3.84E-01 | 3.49E-01 | 2.50E-01 | 4.33E-01 | 2.26E+01 | 2.09E+00 |
| GATA zinc finger | 2.79E+01 | 3.17E+00 | 1.99E+01 | 5.53E+00 | 2.26E+01 | 2.25E+01 | 4.15E+01 | 1.31E+01 |
| GATA zinc finger | 4.16E-01 | 7.58E-02 | 4.98E-01 | 3.52E-01 | 1.42E-01 | 2.46E-01 | 0.00E+00 | 0.00E+00 |
| AP2 domain | 3.99E-01 | 7.63E-02 | 1.54E-03 | 2.67E-03 | 3.50E-01 | 6.07E-01 | 6.09E-01 | 7.83E-02 |
| Helix-loop-helix DNA-binding domain | 2.80E-01 | 2.67E-01 | 3.93E-01 | 1.55E-01 | 1.36E-01 | 2.36E-01 | 1.31E+00 | 1.77E-01 |
| WRKY DNA -binding domain | 1.47E+01 | 3.60E+00 | 1.53E+01 | 3.19E+00 | 1.87E+01 | 3.41E+00 | 1.88E+01 | 1.61E+00 |
| Seed dormancy control | 2.98E+00 | 1.25E+00 | 3.39E+00 | 1.58E+00 | 3.10E+01 | 3.12E+01 | 3.43E+00 | 1.67E+00 |
| GATA zinc finger | 4.01E+00 | 9.16E-01 | 2.27E+00 | 2.31E-01 | 2.08E+00 | 2.00E+00 | 1.15E+00 | 1.82E-01 |
| bZIP transcription factor | 5.95E+00 | 1.01E+00 | 7.56E+00 | 1.04E+00 | 4.01E+00 | 3.55E+00 | 5.38E-01 | 4.67E-01 |
| SRF-type transcription factor | 1.39E+01 | 2.59E+00 | 4.34E+00 | 1.37E+00 | 2.48E+01 | 6.20E+00 | 2.80E+01 | 4.43E+00 |
| Dos2-interacting transcription regulator of RNA-Pol-II | 4.11E+00 | 3.10E-01 | 3.20E+00 | 7.88E-01 | 2.26E+00 | 2.19E+00 | 1.98E+00 | 2.32E-02 |
| Brf1-like TBP-binding domain | 4.20E+00 | 3.15E-01 | 2.38E+00 | 2.00E+00 | 9.83E-01 | 1.70E+00 | 1.92E+00 | 5.95E-01 |
| bZIP transcription factor | 3.26E+00 | 2.70E-01 | 1.73E-01 | 1.51E-01 | 4.94E-01 | 8.56E-01 | 9.80E+00 | 2.10E+00 |
| Helix-loop-helix DNA-binding domain | 3.53E+00 | 1.26E+00 | 3.81E+00 | 2.39E+00 | 3.03E+00 | 2.94E+00 | 0.00E+00 | 0.00E+00 |
| NF-X1 type zinc finger | 1.25E+01 | 8.49E-01 | 7.91E+00 | 1.77E+00 | 2.59E+00 | 4.48E+00 | 2.13E+01 | 1.91E+00 |
| Helix-loop-helix DNA-binding domain | 1.04E+01 | 7.26E-01 | 6.54E+00 | 4.65E+00 | 1.15E+01 | 1.18E+01 | 1.86E+00 | 7.58E-01 |
| AP2 domain | 3.96E-01 | 3.71E-01 | 2.70E+01 | 2.57E+01 | 8.25E+01 | 1.38E+02 | 0.00E+00 | 0.00E+00 |
| AP2 domain | 2.54E+00 | 3.51E-01 | 0.00E+00 | 0.00E+00 | 0.00E+00 | 0.00E+00 | 2.13E+00 | 5.09E-01 |
| TFIID subunit 8 | 3.07E+01 | 4.24E+00 | 2.46E+01 | 1.60E+01 | 7.12E+00 | 1.23E+01 | 1.60E+01 | 3.54E+00 |
| Myb-like DNA-binding domain | 1.20E+01 | 3.32E+00 | 4.92E+00 | 3.45E+00 | 2.10E+00 | 3.63E+00 | 0.00E+00 | 0.00E+00 |
| heat shock transcription factor | 4.08E+00 | 6.48E-01 | 4.39E+00 | 2.25E+00 | 2.09E+00 | 1.83E+00 | 1.19E+01 | 4.61E+00 |
| Myb | 6.44E+00 | 5.70E-01 | 6.04E+00 | 1.05E+00 | 1.54E+00 | 2.64E+00 | 6.86E-01 | 6.76E-01 |
| GATA zinc finger | 4.35E-01 | 1.38E-01 | 4.34E-02 | 7.52E-02 | 3.92E-02 | 6.79E-02 | 0.00E+00 | 0.00E+00 |
| WRKY DNA -binding domain | 7.78E+01 | 6.03E+00 | 5.96E+01 | 8.39E+00 | 2.56E+01 | 2.28E+01 | 9.41E+01 | 6.22E+00 |
| F-box domain | 1.48E+00 | 2.32E-01 | 9.34E-01 | 2.95E-01 | 6.77E-01 | 1.17E+00 | 2.22E+00 | 4.00E-01 |
| Myb | 8.38E-01 | 1.55E-01 | 0.00E+00 | 0.00E+00 | 3.49E-01 | 6.05E-01 | 3.09E-03 | 5.36E-03 |
| WD domain, G-beta repeat | 2.93E+01 | 4.60E+00 | 1.99E+01 | 1.19E+00 | 2.81E+01 | 4.72E+00 | 6.83E+00 | 9.97E-01 |
| AP2 domain | 3.43E+01 | 2.76E+00 | 1.93E+01 | 4.68E+00 | 2.20E+01 | 2.05E+01 | 1.30E+01 | 1.68E+00 |
| WRKY DNA -binding domain | 3.88E+00 | 2.79E+00 | 1.56E-01 | 2.70E-01 | 5.84E-01 | 1.01E+00 | 9.44E+00 | 4.41E+00 |
| K-box region | 1.19E+01 | 1.54E+00 | 1.16E+01 | 5.26E+00 | 5.54E+00 | 4.86E+00 | 4.06E+00 | 4.07E-01 |
| Myb-like DNA-binding domain | 1.42E+00 | 2.05E-01 | 6.00E-01 | 1.80E-01 | 3.13E-01 | 5.43E-01 | 2.83E+01 | 2.03E+00 |
| PB1 domain | 4.77E+00 | 9.27E-01 | 2.10E+00 | 3.16E-01 | 1.32E+00 | 2.25E+00 | 4.11E+00 | 5.89E-01 |
| WRKY DNA -binding domain | 1.24E+01 | 2.77E-01 | 6.90E+00 | 1.84E+00 | 9.80E+00 | 9.36E+00 | 3.60E+01 | 6.79E+00 |
| Myb-like DNA-binding domain | 1.58E+00 | 5.51E-01 | 5.07E-01 | 5.64E-01 | 7.64E-01 | 1.32E+00 | 8.16E+00 | 2.31E+00 |
| Seed dormancy control | 2.37E+00 | 4.99E-01 | 4.07E-01 | 2.99E-01 | 1.03E+00 | 1.79E+00 | 2.69E+00 | 3.10E-01 |
| Protein of unknown function (DUF760) | 1.23E+01 | 1.31E+01 | 3.30E+00 | 3.64E-01 | 1.32E+01 | 1.41E+01 | 2.00E+00 | 5.56E-01 |
| PB1 domain | 1.03E+01 | 1.42E+00 | 3.51E+00 | 9.28E-01 | 2.50E+00 | 2.27E+00 | 4.71E+01 | 1.25E+01 |
| AP2 domain | 2.65E+00 | 8.98E-01 | 1.73E+00 | 6.28E-01 | 6.18E-01 | 1.07E+00 | 0.00E+00 | 0.00E+00 |
| Seed dormancy control | 4.59E+00 | 6.54E-01 | 1.80E+00 | 1.48E+00 | 1.43E+00 | 2.47E+00 | 6.64E+00 | 1.35E+00 |
| Endonuclease/Exonuclease/phosphatase family | 7.61E+00 | 5.22E-01 | 2.82E+00 | 2.87E-01 | 1.44E+00 | 1.87E+00 | 7.48E+00 | 1.93E+00 |
| RNA polymerase II transcription subunit 14 | 1.44E+00 | 1.34E-01 | 2.34E+00 | 2.24E-01 | 1.78E+00 | 1.57E+00 | 1.30E+00 | 1.95E-01 |
| AP2 domain | 8.51E-01 | 1.62E-01 | 1.16E+00 | 8.49E-01 | 3.50E-01 | 6.06E-01 | 5.19E-02 | 5.50E-02 |
| Seed dormancy control | 1.18E+01 | 8.52E-01 | 7.02E+00 | 1.27E+00 | 2.12E+01 | 1.79E+01 | 9.75E+00 | 1.64E+00 |
| Myelodysplasia-myeloid leukemia factor 1-interacting protein | 2.39E+01 | 9.87E-01 | 2.23E+01 | 1.48E+00 | 3.87E+01 | 1.93E+01 | 4.03E+01 | 2.03E+00 |
| TFIIF, beta subunit HTH domain | 1.55E+00 | 2.00E-01 | 4.89E+00 | 4.00E+00 | 1.48E+00 | 2.56E+00 | 7.26E+00 | 3.36E+00 |
| Transcription factor S-II (TFIIS) | 1.56E+01 | 3.01E+00 | 1.70E+01 | 2.05E+00 | 1.59E+01 | 1.46E+01 | 4.63E+00 | 9.63E-01 |
| Myb | 5.10E-01 | 9.19E-02 | 3.03E-01 | 2.56E-01 | 1.12E-01 | 1.94E-01 | 2.25E+00 | 1.07E+00 |
| WRKY DNA -binding domain | 5.80E+00 | 1.55E+00 | 3.82E-01 | 3.31E-01 | 3.01E+00 | 5.22E+00 | 5.04E+00 | 1.56E+00 |
| Plant protein of unknown function | 7.39E-01 | 2.92E-01 | 1.64E-01 | 1.74E-01 | 2.30E-01 | 3.99E-01 | 0.00E+00 | 0.00E+00 |
| Helix-loop-helix DNA-binding domain | 4.03E+00 | 4.11E-01 | 3.71E+00 | 5.09E-01 | 2.26E+00 | 1.96E+00 | 1.33E+00 | 3.41E-01 |
| Myb-like DNA-binding domain | 2.20E+01 | 2.73E+00 | 3.04E+01 | 6.65E+00 | 7.68E+00 | 9.67E+00 | 0.00E+00 | 0.00E+00 |
| Myb-like DNA-binding domain | 3.97E+00 | 2.90E+00 | 3.13E+00 | 1.50E+00 | 1.35E+00 | 2.33E+00 | 0.00E+00 | 0.00E+00 |
| Transcription initiation factor IID, 31kD subunit | 3.92E+00 | 8.28E-01 | 1.35E+00 | 3.81E-01 | 2.56E+00 | 2.22E+00 | 4.16E+00 | 1.02E+00 |
| SRF-type transcription factor | 6.70E+00 | 6.51E-01 | 4.25E+00 | 9.30E-01 | 5.32E+00 | 4.73E+00 | 1.21E+01 | 4.95E-01 |
| Cell differentiation family, Rcd1-like | 8.93E+00 | 6.68E-01 | 4.00E+00 | 1.27E+00 | 1.67E+01 | 1.40E+01 | 3.93E+00 | 6.68E-01 |
| AP2 domain | 3.53E+00 | 1.36E+00 | 2.42E+00 | 8.86E-01 | 9.39E-01 | 1.63E+00 | 9.43E-01 | 1.73E-01 |
| Transcription factor IIA, alpha/beta subunit | 6.78E+00 | 2.32E+00 | 3.42E+00 | 3.88E-01 | 1.12E+00 | 1.94E+00 | 9.57E+00 | 9.12E-01 |
| bZIP transcription factor | 8.35E+00 | 1.17E+00 | 9.88E+00 | 4.04E+00 | 2.93E+00 | 5.08E+00 | 1.05E+00 | 5.22E-01 |
| Targeting protein for Xklp2 (TPX2) domain | 1.61E+01 | 4.68E-01 | 6.14E+00 | 4.07E+00 | 4.46E+00 | 7.73E+00 | 2.50E+01 | 3.53E+00 |
| Plant protein of unknown function | 2.54E+00 | 2.90E-01 | 2.71E+00 | 8.25E-01 | 1.65E+01 | 2.37E+01 | 4.03E+00 | 8.03E-01 |
| WD domain, G-beta repeat | 2.40E+00 | 6.71E-01 | 4.12E+00 | 9.75E-01 | 7.73E-01 | 1.34E+00 | 1.05E+01 | 5.56E-01 |
| mTERF | 1.54E+00 | 4.50E-01 | 1.23E+00 | 2.58E-01 | 9.23E-01 | 8.23E-01 | 4.75E-01 | 2.95E-02 |
| CG-1 domain | 1.67E+01 | 4.15E+00 | 1.15E+01 | 1.99E+00 | 7.93E+00 | 7.34E+00 | 7.16E+00 | 1.86E+00 |
| AP2 domain | 4.27E+01 | 9.36E-01 | 2.57E+01 | 4.71E+00 | 6.28E+01 | 6.11E+01 | 1.47E+01 | 5.99E+00 |
| Targeting protein for Xklp2 (TPX2) domain | 8.38E+00 | 1.82E+00 | 4.64E+00 | 5.00E-01 | 1.10E+01 | 9.26E+00 | 3.94E-02 | 6.83E-02 |
| Bromodomain | 4.49E+00 | 1.29E+00 | 1.32E+00 | 2.71E-01 | 4.75E-01 | 8.23E-01 | 3.73E+00 | 2.60E+00 |
| Myb-like DNA-binding domain | 8.70E-01 | 9.02E-02 | 2.25E-01 | 1.80E-01 | 2.01E-01 | 3.49E-01 | 3.56E-02 | 3.36E-02 |
| Protein of unknown function (DUF760) | 5.98E+01 | 1.34E+00 | 2.60E+01 | 2.81E+00 | 5.61E+01 | 1.34E+01 | 1.38E+02 | 6.67E+00 |
| bHLH-MYC and R2R3-MYB transcription factors N-terminal | 9.71E+00 | 1.57E+00 | 1.26E+01 | 2.28E+00 | 4.87E+00 | 8.43E+00 | 5.31E+01 | 1.15E+00 |
| bZIP transcription factor | 8.08E+00 | 1.37E+00 | 3.11E+00 | 2.18E+00 | 3.38E+00 | 5.84E+00 | 4.75E-01 | 2.20E-02 |
| Mediator complex subunit 25 von Willebrand factor type A | 7.82E+00 | 9.89E-01 | 7.43E+00 | 1.55E+00 | 3.14E+00 | 5.44E+00 | 9.96E+00 | 2.40E+00 |
| WRKY DNA -binding domain | 2.65E+00 | 1.67E+00 | 1.67E+00 | 6.24E-01 | 1.79E-01 | 3.10E-01 | 0.00E+00 | 0.00E+00 |
| mTERF | 7.70E+00 | 2.67E-01 | 9.84E+00 | 4.25E-01 | 4.05E+00 | 3.59E+00 | 1.82E+00 | 3.18E-01 |
| AP2 domain | 1.43E+02 | 1.61E+01 | 1.37E+02 | 2.65E+01 | 1.17E+02 | 1.02E+02 | 2.37E+02 | 3.60E+01 |
| WRKY DNA -binding domain | 1.78E+02 | 5.80E+00 | 2.19E+02 | 2.46E+01 | 1.20E+02 | 1.16E+02 | 2.38E+02 | 1.92E+01 |
| WRKY DNA -binding domain | 1.03E+01 | 7.10E-01 | 6.91E+00 | 9.31E-01 | 5.61E+00 | 5.50E+00 | 4.97E+00 | 1.00E+00 |
| AP2 domain | 0.00E+00 | 0.00E+00 | 0.00E+00 | 0.00E+00 | 0.00E+00 | 0.00E+00 | 3.29E+01 | 3.30E+00 |
| Myb | 3.51E+00 | 1.35E+00 | 8.67E-01 | 7.99E-01 | 1.42E+00 | 2.46E+00 | 3.18E-01 | 4.07E-01 |
| WRKY DNA -binding domain | 1.85E-01 | 8.07E-02 | 1.49E-01 | 2.43E-01 | 7.60E-01 | 1.32E+00 | 9.11E+00 | 4.08E+00 |
| Myb-like DNA-binding domain | 4.72E+00 | 1.44E+00 | 4.60E+00 | 2.15E+00 | 1.82E+01 | 1.35E+01 | 1.21E+01 | 7.14E-01 |
| Myb-like DNA-binding domain | 2.58E+01 | 2.32E+00 | 1.40E+01 | 1.65E+00 | 1.02E+01 | 9.97E+00 | 1.08E+01 | 8.54E-01 |
| Transcription factor subunit Med10 of Mediator complex | 5.06E+00 | 2.37E+00 | 4.12E+00 | 2.33E+00 | 4.32E+00 | 3.78E+00 | 1.21E+00 | 7.35E-02 |
| Myb-like DNA-binding domain | 5.22E-02 | 9.04E-02 | 1.04E-01 | 1.01E-01 | 2.93E-01 | 5.08E-01 | 4.72E+00 | 1.41E+00 |
| E2F/DP family winged-helix DNA-binding domain | 1.90E+00 | 2.09E-01 | 7.56E-01 | 6.55E-01 | 5.97E-01 | 1.03E+00 | 0.00E+00 | 0.00E+00 |
| AP2-like factor | 4.31E-02 | 7.46E-02 | 0.00E+00 | 0.00E+00 | 1.36E-02 | 2.36E-02 | 7.71E-01 | 2.02E-02 |
| WRKY DNA -binding domain | 5.68E-01 | 2.66E-01 | 5.59E-01 | 5.15E-01 | 3.36E-01 | 5.81E-01 | 1.98E+00 | 2.80E-01 |
| K-box region | 5.02E+00 | 1.29E+00 | 5.19E+00 | 9.86E-01 | 9.33E+00 | 9.95E+00 | 1.25E+01 | 2.30E+00 |
| WRKY DNA -binding domain | 7.16E-01 | 2.72E-01 | 4.68E-02 | 8.10E-02 | 4.26E-01 | 7.37E-01 | 2.25E+00 | 1.44E+00 |
| RPAP1-like, N-terminal | 4.84E+00 | 1.03E+00 | 1.63E+00 | 6.31E-01 | 3.45E+00 | 3.42E+00 | 3.66E+00 | 7.09E-01 |
| CAF1 family ribonuclease | 3.20E+00 | 5.72E-01 | 2.35E+00 | 1.24E+00 | 2.03E+00 | 1.86E+00 | 6.57E+00 | 1.41E+00 |
| MIF4G domain | 1.10E+01 | 1.82E+00 | 1.18E+01 | 1.20E+00 | 9.14E+00 | 2.98E+00 | 7.57E+00 | 5.96E-01 |
| Myb-like DNA-binding domain | 4.67E+00 | 8.39E-01 | 4.66E+00 | 3.69E-01 | 2.01E+00 | 3.48E+00 | 8.96E+00 | 3.98E-01 |
| CCAAT-binding transcription factor (CBF-B/NF-YA) subunit B | 7.35E-01 | 2.50E-01 | 2.19E-01 | 6.36E-02 | 1.37E-01 | 2.37E-01 | 6.36E-01 | 1.34E-01 |
| Helix-loop-helix DNA-binding domain | 7.59E+00 | 4.01E-01 | 2.83E+00 | 2.23E+00 | 1.70E+00 | 2.94E+00 | 6.87E-01 | 2.78E-01 |
| Transcription factor subunit Med10 of Mediator complex | 8.02E+00 | 5.41E-01 | 9.35E+00 | 2.62E+00 | 2.70E+00 | 4.67E+00 | 1.61E+01 | 1.45E+00 |
| Plant protein of unknown function | 1.88E-01 | 1.42E-01 | 3.89E-02 | 6.29E-02 | 1.54E-01 | 2.66E-01 | 7.52E+00 | 1.53E+00 |
| MADS-box transcription factor | 1.73E+00 | 1.27E+00 | 5.42E+00 | 1.08E+00 | 6.72E-01 | 1.15E+00 | 5.05E-02 | 7.38E-02 |
| SRF-type transcription factor | 5.06E+00 | 8.53E-01 | 1.08E-01 | 1.88E-01 | 5.86E-01 | 1.02E+00 | 4.56E+00 | 1.78E+00 |
| WRKY DNA -binding domain | 4.76E-02 | 8.24E-02 | 0.00E+00 | 0.00E+00 | 0.00E+00 | 0.00E+00 | 2.13E+00 | 1.13E+00 |
| nuclear transcription factor Y | 3.61E+00 | 1.16E-01 | 1.88E+00 | 2.03E-01 | 1.80E+00 | 3.11E+00 | 7.46E+00 | 4.15E-01 |
| WRKY DNA -binding domain | 1.19E+02 | 5.02E+00 | 7.25E+01 | 1.81E+01 | 5.99E+01 | 7.43E+01 | 1.85E+02 | 4.42E+00 |
| Bromodomain extra-terminal - transcription regulation | 9.17E+00 | 1.20E+00 | 5.67E+00 | 7.38E-01 | 7.52E+00 | 6.76E+00 | 1.21E+01 | 1.82E+00 |
| Myb-like DNA-binding domain | 5.98E-01 | 4.58E-01 | 9.02E-01 | 3.12E-01 | 3.74E-01 | 6.48E-01 | 0.00E+00 | 0.00E+00 |
| AP2 domain | 7.96E-01 | 4.91E-01 | 1.43E+00 | 9.46E-01 | 3.31E-01 | 5.74E-01 | 0.00E+00 | 0.00E+00 |
| Histone-like transcription factor (CBF/NF-Y) | 1.57E+01 | 4.57E+00 | 8.19E+00 | 2.23E+00 | 8.58E+00 | 7.73E+00 | 7.33E+00 | 9.03E-02 |
| Helix-loop-helix DNA-binding domain | 3.88E-01 | 4.97E-02 | 6.11E-02 | 5.99E-02 | 1.80E-01 | 3.11E-01 | 5.85E+00 | 1.32E+00 |
| Myb-like DNA-binding domain | 4.97E-01 | 1.47E-01 | 1.43E-01 | 5.81E-02 | 1.74E-01 | 3.01E-01 | 1.04E-01 | 2.39E-02 |
| PB1 domain | 7.44E+00 | 3.47E-01 | 1.01E+01 | 2.36E+00 | 3.31E+00 | 5.73E+00 | 1.46E+01 | 2.27E+00 |
| bHLH-MYC and R2R3-MYB transcription factors N-terminal | 3.46E+00 | 4.71E-01 | 2.42E+00 | 4.00E-01 | 1.86E+00 | 1.64E+00 | 9.52E-01 | 4.20E-01 |
| Myb | 2.66E-01 | 1.93E-01 | 6.23E-01 | 5.40E-01 | 3.75E-01 | 6.50E-01 | 2.86E+01 | 7.52E-01 |
| Lysine methyltransferase | 4.42E+00 | 2.68E-01 | 1.10E-01 | 1.34E-01 | 5.76E-01 | 9.98E-01 | 9.97E-01 | 6.64E-01 |
| RNA polymerase II transcription subunit 13 | 6.92E-01 | 5.70E-02 | 1.98E-01 | 3.43E-01 | 1.81E-01 | 3.13E-01 | 0.00E+00 | 0.00E+00 |
| transcription factor HY5 | 6.81E+00 | 3.69E+00 | 6.13E+00 | 5.14E+00 | 8.12E+00 | 8.71E+00 | 1.58E+01 | 7.36E-01 |
| GATA zinc finger | 7.22E+00 | 1.72E+00 | 1.70E+01 | 9.14E+00 | 6.61E+00 | 6.83E+00 | 1.35E+00 | 3.36E-01 |
| AP2 domain | 9.34E+00 | 7.06E+00 | 2.36E+00 | 3.18E+00 | 2.66E-01 | 4.61E-01 | 9.56E-02 | 1.66E-01 |
| AP2 domain | 4.12E+00 | 1.18E+00 | 4.72E+00 | 3.28E-01 | 1.75E+00 | 3.04E+00 | 1.83E-01 | 5.99E-02 |
| Leucine rich repeat | 3.86E+00 | 2.37E+00 | 5.73E+00 | 2.95E+00 | 1.00E+01 | 1.54E+01 | 9.47E+00 | 3.84E-01 |
| Myb-like DNA-binding domain | 1.87E+00 | 6.86E-01 | 3.55E+00 | 8.68E-01 | 9.34E-01 | 1.62E+00 | 3.72E-02 | 6.44E-02 |
| HSF-type DNA-binding | 6.43E-01 | 6.74E-01 | 2.56E-01 | 4.93E-02 | 1.09E+00 | 1.89E+00 | 2.46E+00 | 3.25E-01 |
| bZIP transcription factor | 1.11E+00 | 1.75E-01 | 3.47E+00 | 1.04E+00 | 1.09E+00 | 1.16E+00 | 0.00E+00 | 0.00E+00 |
| Helix-loop-helix DNA-binding domain | 2.74E+00 | 2.64E-01 | 2.89E+00 | 2.40E-01 | 9.45E-01 | 9.07E-01 | 6.91E+00 | 3.94E-02 |
| AP2 domain | 1.01E+00 | 1.19E-01 | 3.55E-01 | 1.34E-01 | 3.51E-01 | 6.09E-01 | 0.00E+00 | 0.00E+00 |
| HSF-type DNA-binding | 0.00E+00 | 0.00E+00 | 0.00E+00 | 0.00E+00 | 1.41E-03 | 2.44E-03 | 2.37E+00 | 2.15E+00 |
| Histone-like transcription factor (CBF/NF-Y) | 5.93E+00 | 1.05E+00 | 2.75E+00 | 3.59E+00 | 3.24E-01 | 5.61E-01 | 1.65E+00 | 8.01E-01 |
| HSF-type DNA-binding | 2.87E+01 | 3.57E+00 | 1.00E+01 | 6.30E+00 | 9.51E+00 | 1.65E+01 | 2.31E+01 | 4.13E+00 |
| Early transcription elongation factor of RNA pol II, NGN section | 3.68E+00 | 1.10E+00 | 4.34E+00 | 1.36E+00 | 1.00E+00 | 1.73E+00 | 1.21E+00 | 2.86E-01 |
| WRKY DNA -binding domain | 2.45E+00 | 2.75E-01 | 2.40E+00 | 5.43E-01 | 3.24E+00 | 2.83E+00 | 4.74E+00 | 1.07E+00 |
| Transcription initiation factor IIF, alpha subunit (TFIIF-alpha) | 1.22E+01 | 5.82E-01 | 9.02E+00 | 8.15E-01 | 7.76E+00 | 7.23E+00 | 1.61E+01 | 1.87E+00 |
| Myb-like DNA-binding domain | 1.04E+01 | 1.97E+00 | 5.99E+00 | 3.98E+00 | 4.29E+00 | 7.44E+00 | 4.65E+00 | 5.31E-01 |
| Helix-loop-helix DNA-binding domain | 3.38E+00 | 4.62E-01 | 2.33E+00 | 2.04E+00 | 3.36E+01 | 3.20E+01 | 1.12E+01 | 4.47E-01 |
| bHLH-MYC and R2R3-MYB transcription factors N-terminal | 2.40E+01 | 3.47E+00 | 1.81E+01 | 1.99E+00 | 7.46E+00 | 1.29E+01 | 8.76E+00 | 1.19E+00 |
| CG-1 domain | 6.65E+01 | 5.21E+00 | 3.12E+01 | 2.12E+00 | 2.87E+01 | 3.49E+01 | 4.86E+01 | 3.44E+00 |
| MYB | 3.90E+00 | 1.57E+00 | 4.43E+00 | 1.05E+00 | 1.45E+00 | 2.52E+00 | 2.53E-01 | 2.53E-01 |
| RNA polymerase I specific transcription initiation factor RRN3 | 4.81E+00 | 5.18E-01 | 8.93E+00 | 2.44E+00 | 1.65E+00 | 2.86E+00 | 6.77E+00 | 8.90E-01 |
| SRF-type transcription factor | 1.68E-01 | 2.01E-01 | 2.72E-01 | 1.49E-01 | 1.19E-01 | 2.06E-01 | 6.97E-01 | 8.81E-02 |
| PB1 domain | 1.14E+01 | 3.07E-01 | 5.54E+00 | 8.49E-01 | 3.23E+00 | 5.53E+00 | 2.80E+01 | 3.56E+00 |
| CCR4-NOT transcription complex subunit 6 | 1.44E+01 | 4.65E-01 | 2.00E+01 | 1.33E+00 | 1.13E+01 | 1.03E+01 | 2.34E+01 | 5.24E+00 |
| Seed dormancy control | 1.68E+01 | 3.64E-01 | 1.15E+01 | 7.41E-01 | 9.64E+00 | 9.03E+00 | 2.74E+01 | 1.67E+00 |
| IQ calmodulin-binding motif | 5.43E+00 | 1.49E+00 | 5.00E+00 | 1.16E+00 | 9.70E+00 | 4.54E+00 | 2.63E+00 | 8.09E-01 |
| WRKY DNA -binding domain | 4.35E+00 | 6.40E-01 | 5.26E-02 | 5.82E-02 | 1.53E+00 | 2.65E+00 | 4.06E+00 | 1.21E+00 |
| Helix-loop-helix DNA-binding domain | 3.65E-01 | 1.47E-02 | 5.71E-01 | 2.86E-01 | 1.64E-01 | 2.85E-01 | 3.83E+00 | 1.51E+00 |
| WRKY DNA -binding domain | 2.19E-01 | 5.59E-02 | 6.40E-02 | 6.00E-02 | 3.42E-01 | 5.93E-01 | 7.22E-01 | 1.86E-01 |
| Core histone H2A/H2B/H3/H4 | 1.11E+00 | 7.09E-01 | 0.00E+00 | 0.00E+00 | 0.00E+00 | 0.00E+00 | 2.97E+00 | 9.04E-01 |
| Multiprotein bridging factor 1 | 3.36E+01 | 5.77E+00 | 1.79E+01 | 1.38E+01 | 8.44E+00 | 1.46E+01 | 1.13E+01 | 2.13E+00 |
| plant G-box-binding factor | 2.96E+01 | 1.48E+00 | 3.65E+01 | 8.58E+00 | 4.19E+01 | 3.79E+01 | 2.08E+01 | 8.74E+00 |
| Myb-like DNA-binding domain | 2.67E+00 | 6.12E-01 | 2.00E+00 | 1.08E+00 | 6.65E-01 | 1.15E+00 | 6.49E+00 | 1.42E+00 |
| MYB | 4.61E+00 | 1.10E+00 | 8.81E+00 | 1.27E+00 | 3.01E+00 | 5.21E+00 | 6.66E-01 | 2.98E-01 |
| WRKY DNA -binding domain | 5.99E+00 | 1.33E+00 | 7.98E+00 | 2.38E+00 | 4.78E+00 | 4.36E+00 | 1.34E+00 | 3.95E-01 |
| WRKY DNA -binding domain | 9.77E+00 | 3.50E+00 | 4.20E+00 | 1.40E+00 | 2.25E+00 | 3.91E+00 | 1.55E+01 | 8.05E-01 |
| Helix-loop-helix DNA-binding domain | 5.30E+00 | 8.25E-01 | 4.18E+00 | 1.72E+00 | 9.11E-01 | 1.58E+00 | 1.19E+00 | 5.79E-01 |
| heat shock transcription factor | 3.72E+00 | 2.60E-01 | 1.38E+00 | 1.01E+00 | 6.21E+00 | 4.98E+00 | 6.61E+00 | 4.20E-01 |
| HSF-type DNA-binding | 6.34E+00 | 6.18E-01 | 1.23E+00 | 2.13E+00 | 0.00E+00 | 0.00E+00 | 0.00E+00 | 0.00E+00 |
| TFIIF, beta subunit HTH domain | 2.18E+00 | 1.79E+00 | 1.25E+00 | 9.38E-01 | 4.05E-01 | 7.01E-01 | 1.23E+01 | 3.06E-01 |
| bHLH-MYC and R2R3-MYB transcription factors N-terminal | 5.11E+00 | 2.38E+00 | 1.28E+01 | 2.09E+00 | 2.32E+00 | 4.02E+00 | 3.73E+00 | 2.50E-01 |
| Endonuclease/Exonuclease/phosphatase family | 8.41E+00 | 1.14E+00 | 1.08E+01 | 5.96E-01 | 3.52E+00 | 6.10E+00 | 6.11E+00 | 8.92E-01 |
| AP2 domain | 5.74E-01 | 2.07E-01 | 3.03E-01 | 2.79E-01 | 0.00E+00 | 0.00E+00 | 0.00E+00 | 0.00E+00 |
| Myb-like DNA-binding domain | 2.03E+01 | 1.21E+00 | 1.41E+01 | 1.74E+00 | 7.16E+00 | 1.24E+01 | 2.98E+01 | 2.49E+00 |
| MID domain of medPIWI | 9.87E+00 | 1.71E+00 | 1.09E+01 | 1.02E+00 | 1.46E+01 | 8.11E+00 | 7.00E+00 | 5.79E-01 |
| WRKY DNA -binding domain | 1.31E+01 | 2.09E+00 | 9.35E+00 | 2.98E+00 | 5.10E+00 | 8.83E+00 | 2.15E+01 | 7.49E-01 |
| WD domain, G-beta repeat | 1.14E+01 | 3.71E-01 | 8.39E+00 | 5.37E+00 | 4.96E+00 | 8.59E+00 | 3.27E+01 | 1.33E+01 |
| Myb-like DNA-binding domain | 4.53E-01 | 2.15E-01 | 3.58E-01 | 4.10E-01 | 6.30E-02 | 1.09E-01 | 7.60E+00 | 5.08E-01 |
| WRKY DNA -binding domain | 6.58E+01 | 5.13E+00 | 1.62E+01 | 5.58E+00 | 3.89E+01 | 2.01E+01 | 3.51E+01 | 7.65E+00 |
| SBP domain | 1.28E+01 | 8.70E-01 | 1.15E+01 | 3.18E+00 | 7.64E+00 | 7.47E+00 | 1.62E+01 | 3.53E+00 |
| Helix-loop-helix DNA-binding domain | 1.09E+00 | 3.45E-01 | 8.76E-02 | 7.61E-02 | 3.56E-01 | 6.17E-01 | 1.46E+00 | 9.12E-01 |
| Endonuclease/Exonuclease/phosphatase family | 6.95E+00 | 1.05E+00 | 7.26E+00 | 3.98E-01 | 1.81E+00 | 3.13E+00 | 1.10E+01 | 6.09E-01 |
| GATA zinc finger | 2.49E+00 | 3.19E-01 | 2.25E+00 | 8.32E-02 | 6.78E-01 | 1.17E+00 | 0.00E+00 | 0.00E+00 |
| B3 DNA binding domain | 6.95E+00 | 1.34E+00 | 5.35E+00 | 9.86E-01 | 2.02E+00 | 3.50E+00 | 9.10E+00 | 1.10E+00 |
| GATA zinc finger | 1.27E+01 | 2.03E+00 | 7.48E+00 | 1.37E+00 | 9.31E+00 | 8.25E+00 | 3.15E+01 | 8.03E-01 |
| Myelodysplasia-myeloid leukemia factor 1-interacting protein | 2.77E+00 | 9.99E-01 | 1.41E+00 | 1.20E+00 | 8.90E-01 | 1.54E+00 | 9.24E+00 | 1.09E+00 |
| WRKY DNA -binding domain | 6.79E+00 | 1.55E+00 | 7.91E+00 | 5.03E+00 | 2.13E+00 | 3.69E+00 | 1.11E+01 | 7.40E-01 |
| mTERF | 7.84E+00 | 1.75E-01 | 9.98E+00 | 9.42E-01 | 4.57E+00 | 3.99E+00 | 2.11E+00 | 1.22E+00 |
| WRKY DNA -binding domain | 2.35E+01 | 2.82E+00 | 1.35E+01 | 4.15E+00 | 1.15E+01 | 1.10E+01 | 4.98E+01 | 4.70E+00 |
| Myb-like DNA-binding domain | 3.66E+00 | 2.20E+00 | 1.21E+00 | 7.97E-01 | 5.91E-01 | 1.02E+00 | 0.00E+00 | 0.00E+00 |
| GATA zinc finger | 3.71E+00 | 1.64E+00 | 2.15E+00 | 2.66E-01 | 1.43E+00 | 2.48E+00 | 1.21E+00 | 4.29E-01 |
| Helix-loop-helix DNA-binding domain | 2.81E+00 | 2.15E+00 | 9.04E+00 | 5.93E-01 | 1.72E+00 | 2.98E+00 | 1.34E-02 | 1.18E-02 |
| Helix-loop-helix DNA-binding domain | 1.32E+00 | 2.93E-01 | 7.69E-01 | 4.05E-01 | 4.20E-01 | 7.27E-01 | 3.89E-01 | 1.82E-01 |
| FAT domain | 1.56E+01 | 1.21E+00 | 1.87E+01 | 3.02E+00 | 5.44E+00 | 9.43E+00 | 1.26E+01 | 1.49E+00 |
| Transcription factor TFIIB repeat | 1.74E+01 | 3.49E+00 | 1.70E+01 | 9.24E-01 | 1.72E+01 | 1.95E+01 | 3.60E+01 | 7.76E-01 |
| HSF-type DNA-binding | 1.88E-01 | 5.68E-02 | 0.00E+00 | 0.00E+00 | 4.40E-02 | 7.62E-02 | 2.10E+01 | 2.04E+00 |
| Helix-loop-helix DNA-binding domain | 3.18E-01 | 3.30E-01 | 3.45E-01 | 1.06E-01 | 1.85E-01 | 3.20E-01 | 6.97E+00 | 4.77E+00 |
| Helix-loop-helix DNA-binding domain | 4.70E+00 | 5.89E-01 | 5.01E-01 | 3.36E-01 | 1.46E+00 | 2.52E+00 | 0.00E+00 | 0.00E+00 |
| Helix-loop-helix DNA-binding domain | 1.17E+01 | 7.19E-01 | 1.12E+01 | 7.33E+00 | 9.47E+00 | 8.21E+00 | 3.09E+00 | 4.55E-01 |
| Scavenger mRNA decapping enzyme C-term binding | 3.06E+00 | 2.20E+00 | 9.51E-01 | 4.07E-01 | 4.17E-01 | 7.23E-01 | 2.63E+00 | 3.69E-01 |
| SRF-type transcription factor | 2.89E+00 | 3.80E-01 | 1.43E+00 | 8.85E-01 | 1.23E+00 | 2.12E+00 | 5.80E+00 | 5.76E-01 |
| PB1 domain | 2.15E+00 | 1.19E+00 | 2.18E+00 | 2.56E+00 | 4.56E-02 | 7.89E-02 | 3.22E+00 | 2.05E+00 |
| Myb-like DNA-binding domain | 1.06E+00 | 2.27E-01 | 6.15E-01 | 4.69E-01 | 6.20E-01 | 1.07E+00 | 4.60E+00 | 1.56E-01 |
| HSF-type DNA-binding | 5.38E-02 | 9.32E-02 | 1.54E-02 | 2.67E-02 | 0.00E+00 | 0.00E+00 | 1.96E+00 | 1.00E+00 |
| AP2 domain | 1.52E+00 | 7.22E-01 | 1.05E+00 | 9.52E-02 | 5.75E-01 | 9.96E-01 | 0.00E+00 | 0.00E+00 |
| SOH1 | 7.02E-01 | 1.77E-01 | 1.20E-01 | 2.08E-01 | 5.37E-02 | 9.30E-02 | 0.00E+00 | 0.00E+00 |
| Targeting protein for Xklp2 (TPX2) domain | 2.31E+00 | 3.68E-01 | 8.21E-01 | 6.23E-01 | 7.28E-01 | 1.26E+00 | 0.00E+00 | 0.00E+00 |
| Myb-like DNA-binding domain | 1.09E+00 | 2.99E-01 | 9.41E-01 | 8.15E-01 | 3.51E-01 | 6.09E-01 | 7.88E-03 | 1.37E-02 |
| WRKY DNA -binding domain | 2.55E+00 | 4.21E-01 | 3.23E-01 | 2.85E-01 | 6.31E-01 | 5.83E-01 | 2.53E+01 | 2.69E+00 |
| ERCC3/RAD25/XPB C-terminal helicase | 6.84E+00 | 1.80E+00 | 3.15E+00 | 2.99E-01 | 2.52E+00 | 4.37E+00 | 1.01E+01 | 2.22E+00 |
| MYB | 2.58E+00 | 9.48E-01 | 6.15E-01 | 3.48E-01 | 2.79E-01 | 4.83E-01 | 1.51E+01 | 2.66E+00 |
| Myb-like DNA-binding domain | 7.77E+00 | 5.74E-01 | 5.22E+00 | 7.32E-01 | 1.03E+01 | 4.56E+00 | 2.75E+00 | 6.96E-01 |
| Protein of unknown function (DUF760) | 1.39E+01 | 1.12E+00 | 2.85E+01 | 2.64E+00 | 1.97E+01 | 2.38E+01 | 3.57E+01 | 6.65E+00 |
| WRKY DNA -binding domain | 1.14E+02 | 5.50E+00 | 7.48E+01 | 1.85E+01 | 1.46E+02 | 5.59E+01 | 1.43E+02 | 3.52E+01 |
| Helix-loop-helix DNA-binding domain | 4.66E+00 | 2.05E+00 | 5.60E+00 | 3.38E+00 | 8.30E+00 | 3.08E+00 | 1.94E+01 | 2.00E+00 |
| Myb-like DNA-binding domain | 2.57E+00 | 6.51E-01 | 1.44E+00 | 5.74E-01 | 1.55E+00 | 1.42E+00 | 3.74E-01 | 1.62E-01 |
| SH2 domain | 1.32E+01 | 1.04E+00 | 1.66E+01 | 1.34E+00 | 5.46E+00 | 8.29E+00 | 1.09E+01 | 5.53E-01 |
| Myb-like DNA-binding domain | 1.52E+00 | 6.30E-01 | 1.74E+00 | 1.74E+00 | 1.07E+00 | 1.86E+00 | 4.00E+00 | 6.47E-01 |
| bZIP transcription factor | 6.37E-01 | 2.79E-01 | 9.07E-01 | 6.48E-01 | 2.53E-01 | 4.39E-01 | 0.00E+00 | 0.00E+00 |
| AP2 domain | 7.90E+00 | 3.55E+00 | 1.95E+01 | 1.12E+01 | 3.33E+00 | 5.77E+00 | 3.27E+00 | 1.77E-01 |
| WRKY DNA -binding domain | 2.52E-01 | 5.19E-02 | 1.39E-01 | 1.43E-01 | 3.08E-01 | 5.34E-01 | 5.27E+00 | 9.01E-01 |
| HMG (high mobility group) box | 5.79E+00 | 1.05E+00 | 3.43E+00 | 5.18E-01 | 9.76E-01 | 1.69E+00 | 0.00E+00 | 0.00E+00 |
| Seed dormancy control | 5.98E-01 | 6.56E-01 | 4.64E-01 | 1.09E-01 | 0.00E+00 | 0.00E+00 | 0.00E+00 | 0.00E+00 |
| Myb-like DNA-binding domain | 3.95E+00 | 2.36E+00 | 3.65E+00 | 2.05E+00 | 3.95E+00 | 3.42E+00 | 7.80E-02 | 1.10E-01 |
| Targeting protein for Xklp2 (TPX2) domain | 3.18E+00 | 3.14E-01 | 7.79E+00 | 4.11E+00 | 3.87E+00 | 6.71E+00 | 1.78E+00 | 1.40E+00 |
| Helix-loop-helix DNA-binding domain | 6.71E-01 | 1.65E-01 | 7.53E-01 | 3.96E-01 | 2.84E-01 | 4.92E-01 | 2.99E-02 | 2.60E-02 |
| Helix-loop-helix DNA-binding domain | 1.84E+00 | 2.46E-01 | 1.06E+01 | 6.00E+00 | 5.05E-01 | 8.74E-01 | 0.00E+00 | 0.00E+00 |
| MYB-related transcription factor LHY | 5.93E-01 | 1.14E-01 | 7.04E-01 | 4.59E-01 | 2.95E-01 | 3.91E-01 | 3.11E+01 | 1.30E+00 |
| bZIP transcription factor | 5.92E+00 | 5.13E-01 | 2.64E+00 | 2.27E+00 | 1.20E+01 | 7.15E+00 | 7.22E-02 | 6.28E-02 |
| Transcription initiation factor IID, 31kD subunit | 3.20E+00 | 2.34E-01 | 3.92E+00 | 4.10E-01 | 8.79E-01 | 1.52E+00 | 4.87E+00 | 9.50E-01 |
| Myb-like DNA-binding domain | 7.42E-01 | 4.05E-01 | 3.26E+00 | 1.03E+00 | 5.07E-02 | 8.79E-02 | 0.00E+00 | 0.00E+00 |
| bHLH-MYC and R2R3-MYB transcription factors N-terminal | 1.11E+00 | 1.56E-01 | 8.31E-01 | 3.29E-01 | 5.79E-01 | 9.31E-01 | 1.12E-01 | 1.01E-01 |
| MAC/Perforin domain | 1.89E+01 | 7.25E+00 | 5.82E+00 | 6.09E-01 | 2.56E+00 | 4.43E+00 | 6.24E+00 | 2.85E+00 |
| Helix-loop-helix DNA-binding domain | 3.58E+00 | 5.38E-01 | 8.52E+00 | 1.44E+00 | 2.11E+00 | 3.65E+00 | 1.50E+00 | 3.83E-01 |
| GATA zinc finger | 5.29E+00 | 2.86E-01 | 8.02E+00 | 3.85E+00 | 6.61E-01 | 1.14E+00 | 2.15E+01 | 4.57E+00 |
| Myb-like DNA-binding domain | 1.04E+00 | 2.11E-01 | 7.83E-01 | 1.06E+00 | 5.51E-01 | 9.54E-01 | 0.00E+00 | 0.00E+00 |
| CAF1 family ribonuclease | 1.32E+02 | 1.05E+01 | 1.08E+02 | 9.51E+00 | 5.25E+01 | 9.09E+01 | 0.00E+00 | 0.00E+00 |
| HSF-type DNA-binding | 4.71E+01 | 1.12E+01 | 2.11E+00 | 6.74E-01 | 1.65E+01 | 1.90E+01 | 2.98E+01 | 6.89E+00 |
| TFIIS helical bundle-like domain | 1.16E+01 | 2.14E+00 | 1.03E+01 | 2.78E+00 | 1.95E+01 | 6.97E+00 | 1.70E+01 | 1.56E+00 |
| Myb-like DNA-binding domain | 1.25E+01 | 1.80E+00 | 6.26E+00 | 1.21E+00 | 1.99E+01 | 1.89E+01 | 1.69E+00 | 4.61E-01 |
| MYB | 5.53E+00 | 6.10E-01 | 6.47E+00 | 9.49E-01 | 3.04E+00 | 5.27E+00 | 2.94E+00 | 6.66E-01 |
| CCAAT-binding transcription factor (CBF-B/NF-YA) subunit B | 3.99E+00 | 3.62E-01 | 2.59E+00 | 7.18E-01 | 2.43E+00 | 2.30E+00 | 4.58E+00 | 1.27E+00 |
| Helix-loop-helix DNA-binding domain | 8.53E-01 | 1.49E-01 | 2.02E-01 | 1.80E-01 | 3.04E-01 | 5.26E-01 | 0.00E+00 | 0.00E+00 |
| Helix-loop-helix DNA-binding domain | 1.08E+00 | 2.75E-02 | 1.10E+00 | 3.53E-01 | 3.55E+00 | 3.44E+00 | 3.97E+00 | 7.41E-01 |
| Targeting protein for Xklp2 (TPX2) domain | 9.02E+00 | 2.50E+00 | 1.42E+01 | 1.29E+00 | 7.49E+00 | 7.61E+00 | 2.79E+00 | 1.74E-01 |
| AP2 domain | 2.32E+01 | 2.18E+00 | 2.20E+01 | 3.75E+00 | 3.45E+01 | 3.50E+01 | 4.86E+01 | 4.90E+00 |
| mTERF | 4.41E+00 | 5.56E-01 | 2.69E+00 | 2.83E-01 | 1.11E+00 | 1.93E+00 | 1.16E+00 | 1.02E-01 |
| Targeting protein for Xklp2 (TPX2) domain | 1.66E+01 | 5.78E-01 | 1.39E+01 | 1.29E+00 | 1.25E+01 | 2.61E+00 | 6.31E+00 | 6.62E-01 |
| Transcription factor IIA, alpha/beta subunit | 8.61E+00 | 2.56E+00 | 3.34E+00 | 1.83E+00 | 4.63E+00 | 4.30E+00 | 1.24E+01 | 4.54E+00 |
| bHLH-MYC and R2R3-MYB transcription factors N-terminal | 2.08E+01 | 3.61E+01 | 0.00E+00 | 0.00E+00 | 3.83E+02 | 6.63E+02 | 1.36E+02 | 1.58E+01 |
| HSF-type DNA-binding | 1.46E+01 | 3.08E+00 | 2.16E+00 | 1.78E+00 | 1.48E+01 | 1.70E+01 | 9.68E+00 | 3.13E+00 |
| K-box region | 1.70E+00 | 1.74E-01 | 1.05E+00 | 2.22E-01 | 6.04E-01 | 1.05E+00 | 7.27E+00 | 1.06E+00 |
| Myb-like DNA-binding domain | 4.88E-01 | 7.88E-01 | 0.00E+00 | 0.00E+00 | 3.71E-02 | 6.43E-02 | 3.55E+00 | 3.65E+00 |
